# Changes in American adolescents’ outdoor time over two decades

**DOI:** 10.1073/pnas.2605134123

**Published:** 2026-10-26

**Authors:** Ewa Jarosz, Vidar Ulset, Vegard Skirbekk

**Affiliations:** ^a^ Department of Psychology, University of Oslo, Oslo 0373, Norway; ^b^ Norwegian School of Sport Sciences, Department of Teacher Education and Outdoor Studies, Oslo 0863, Norway; ^c^ Norwegian Institute of Public Health, Centre for Fertility and Health, Oslo 0473, Norway

**Keywords:** nature, adolescence, outdoor, leisure behaviors, time use

## Abstract

Spending time outdoors provides opportunities for physical activity, exposure to natural light, and social contact. Although young people’s time use has changed substantially in recent decades, little is known about long-term trends in adolescents’ outdoor engagement. This question is especially important because adolescence is a period of major life transitions and the development of lasting behavioral habits. This study examines 20-y trends in time spent outdoors among American adolescents using time-diary data from 14,235 individuals aged 15 to 19 in the 2003–2023 American Time Use Survey, a nationally representative survey of the US population. We describe trends in outdoor exposure and examine associations between outdoor time and leisure behaviors, including computer use for leisure, indoor physical activity, and time spent with friends. We find that outdoor engagement among boys has declined sharply, erasing a long-standing male advantage and causing boys’ participation levels to converge with girls’ at similarly low rates. Greater time spent in computer-based leisure was associated with less outdoor time, particularly among boys, whereas time spent with friends was positively associated with outdoor engagement for both sexes. Indoor physical activity did not change over the observation period and did not offset declines in outdoor time. These findings suggest that broader changes in leisure activities may be reshaping how young people engage with outdoor environments. Further research is needed to better understand the factors underlying these trends and their potential long-term implications.

## Significance

This study uses detailed behavioral data to document changes in US adolescents’ outdoor time over the past two decades. Spending time outdoors supports physical activity and daylight exposure and shapes opportunities for social interaction and engagement with the outside environment. We observe a downward trend in outdoor time among boys, persistently low levels among girls, and a negative association between computer-based leisure and outdoor time. Because habits formed in adolescence often persist into adulthood, these behaviors may have long-term consequences. The implications of the observed declines in outdoor time may extend beyond recreation, contributing to population-level increases in sedentary behavior and greater disconnectedness from the natural world.

Over recent decades, rapid social and technological changes have reshaped young people’s leisure activities. Although studies suggest that both unstructured outdoor play (1) and independent outdoor mobility (2) (moving around without a parent) have declined among children compared with earlier generations, population-level evidence on the amount of time spent outdoors by young people remains limited. This study examines 20-y trends in outdoor time among American teenagers and identifies environmental, social, and behavioral factors associated with youths’ engagement in outdoor activities.

Time spent outdoors can provide a unique combination of benefits, including opportunities for movement and exposure to daylight, which is inherently specific to outdoor settings. Exposure to natural light regulates circadian rhythm (3), sleep patterns (4), and blood glucose levels (5). More time spent outdoors is also associated with a lower risk and slower progression of myopia during childhood (6). Daylight exposure has been proposed as one mechanism underlying this association, potentially through effects on axial eye growth (7). Childhood myopia rates are increasing globally, with decreased outdoor time implicated as a key behavioral risk factor (8, 9).

Outdoor time is also a major source of daily physical activity (PA). Among youth, while just about 13% of total time is spent outdoors, nearly 30% of overall physical activity occurs in outdoor settings (10). During outdoor leisure time, adolescents also engage in significantly higher levels of moderate-to-vigorous physical activity (MVPA) than during indoor leisure time (11). Consistent with these behavioral patterns, outdoor time is also associated with greater cardiorespiratory fitness (12), and better mental health, with PA mediating many of these benefits (13). Self-reported benefits of spending time outdoors among adolescents also include improved mood and feeling physically healthier (14, 15).

Among American adults, time spent outdoors is associated with lower prevalence of chronic diseases, with only part of this association being accounted for by PA levels (16) suggesting that outdoor exposure may influence morbidity in adulthood through multiple pathways. For example, reduced psychological stress may represent another mechanism linking time spent outdoors to health. Cumulative impact of repeated stress is reflected in allostatic load (17), a measure linked with increased all-cause mortality (18). A systematic review of 43 experimental studies using adult populations found some evidence for reductions in stress markers such as heart rate, blood pressure, and self-reported stress following outdoor exposure (19). However, the authors noted substantial variation in the types of outdoor settings, which likely contributed to heterogeneity in findings. Consistent with this concern, a meta-analysis of 31 studies found that exposure to green environments resulted in marked reductions in stress markers compared with exposure to built environments (20).

However, access to green spaces may not be equally distributed or easily accessible. In cities, green areas such as parks and gardens are relatively scarce in more socially disadvantaged areas compared to more affluent neighborhoods (21). Emerging evidence from a high-income society indicate that adolescent boys frequently report a shortage of accessible “real-world” spaces, such as parks, for recreation, potentially contributing to a greater reliance on digital environments for social engagement and leisure (22). There is also concern that outdoor spaces in America are becoming less accessible as they are deliberately designed to discourage teenagers’ “hanging out” (23).

In developed countries, most of the outdoor exposure comes from leisure time activities (24) so trends in adolescents’ outdoor use should be analyzed in the context of their rapidly changing leisure behaviors. In particular, online leisure has increased substantially over a relatively short period, with time spent online by American teenagers doubling between 2006 and 2016 (25). During the same period, the consumption of printed media and TV declined suggesting that online recreation might be replacing previously popular leisure options (25).

Another major social trend is a documented decline in time spent with friends and family in the American population (26). Among youth, time spent outdoors has been positively associated with time spent on social interactions. The relationship appears to be bidirectional: greater opportunities for social interaction are associated with increased time outdoors (27), and time spent outdoors, in turn, facilitates both planned and spontaneous social interactions (28). The ease of getting one’s friends to “hang out” could thus be another factor linked with outdoor recreation, and vice versa—being outside could increase the opportunity for social exposure.

Getting friends to join in may also have become more challenging, as over the past decade young people have faced increasing time pressures related to schoolwork (29). Perceived time pressure is more common among adolescent girls, who experience greater declines in their free time with age than boys (30). Alongside increasing time pressure, individuals may shift the settings in which they engage in physical activity away from outdoor environments toward alternative locations. There is evidence that youth have decreased their time in outdoor-based recreation while increasing their use of fitness centers (31). This trend has been attributed to the convenience of indoor gyms amid competing demands on adolescents’ time and increasing focus on fitness gains rather than general recreation.

The above trends in adolescents’ behaviors are unfolding during a developmental period characterized by major physical and emotional transitions (32). While some important changes, such as increasing digital engagement and decline in social interaction, have been documented in earlier studies, similar knowledge is lacking for outdoor time. Outdoor exposure in early life—in particular during middle adolescence (15 to 17 y old)—shapes the patterns of outdoor use in adulthood (33). Adolescence is therefore a critical period during which outdoor activity habits become consolidated, and low outdoor engagement during this time may have long-term implications.

This study uses multiyear microdata from the 2003–2023 American Time Use Survey (US Bureau of Labor Statistics, 2003–2023) (34). We describe long-term trends in outdoor time among American adolescents aged 15 to 19 and examine how outdoor time is associated with sociodemographic characteristics and selected leisure behaviors, including computer-based leisure, time spent with friends, and indoor physical activity. By placing outdoor time within broader shifts in adolescent leisure, we aim to investigate whether engagement with outdoor environments has changed over time, and which behavioral patterns may be linked to these changes.

## Results

**Fig. 1.**
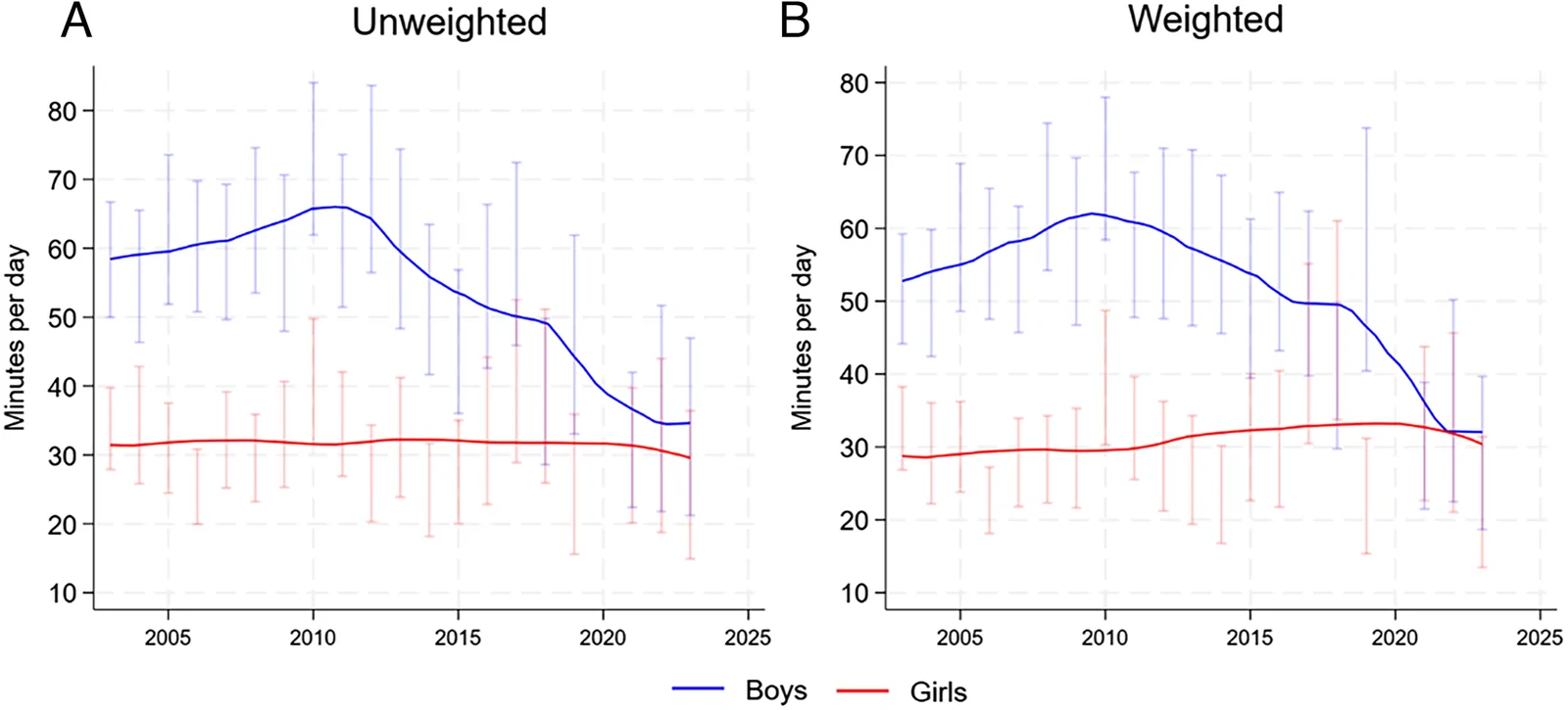
Mean time (in mins per day) spent outdoors among ATUS respondents aged 15 to 19, by sex and year, smoothed and (*A*) unweighted, (*B*) weighted using ATUS population weights. Notes: Annual averages across 2003–2023; year 2020 is zero-weighted/not included in the trend due to COVID-19-related data distortions.

The overall time spent outdoors by all adolescents (boys and girls) declined between 2003 and 2023. Analyses by sex and year show that the decline started after around 2011 and is entirely attributable to the trend among boys (Fig. 1 and *SI Appendix*, Fig. S1 shows nonsmoothed and nonweighted trend lines). Around 2010, American boys spent over 60 min outdoors, on an average day. This number dropped to under 40 min in 2023. American girls spent significantly less time outdoors than boys back in 2010 but the differences between sexes became nonsignificant in the more recent years.

This downward trend among boys shows also in adjusted models (Table 1 and *SI Appendix*, Table S4 shows the coefficients for all variables included in the model; *SI Appendix*, Table S5 shows the analyses narrowed down to years 2010–2023), with the most marked change in the period following the pandemic when both incidence and duration of outdoor activity decline. There are no significant period differences in outdoor time incidence among girls, but the duration shows minor decline over time. The associations between social and environmental covariates of outdoor time do not differ much by sex. Both adolescent boys and girls spend more time outdoors when they are younger (under 16), live in Northeast or West, and live in households with higher incomes. They are more likely to be outside during weekdays, but those who go out spend more time outdoors on weekends. Black boys and girls are more likely to go out but they do so for shorter durations. We also see expectable seasonal activity patterns with more outdoor time occurring in the summer. Finally, girls living in metropolitan areas are more likely to go outside compared to girls living in nonmetropolitan areas, with no similar effects observed among boys.

**Table 1.** Hurdle model of outdoor time (incidence and duration)

	Logit: Incidence (any time outside)			
	Boys		Girls	
	OR [CI 95%]	*P*	OR [CI 95%]	*P*
Period (ref. 2003–2010)				
2011–2019	**0.702 [0.551,0.895]**	**0.004**	0.935 [0.728,1.201]	0.597
2020	**0.437 [0.302,0.633]**	**0.000**	0.866 [0.579,1.294]	0.482
2021–2023	**0.508 [0.375,0.688]**	**0.000**	0.886 [0.651,1.207]	0.443
Age (ref. 15)				
16–17	**0.645 [0.573,0.726]**	**0.000**	**0.684 [0.605,0.773]**	**0.000**
18–19	**0.497 [0.435,0.569]**	**0.000**	**0.627 [0.546,0.719]**	**0.000**
Region (ref. Northeast)				
Midwest	**0.767 [0.659,0.893]**	**0.001**	**0.729 [0.623,0.854]**	**0.000**
South	**0.756 [0.656,0.872]**	**0.000**	**0.630 [0.543,0.731]**	**0.000**
West	1.042 [0.896,1.211]	0.593	0.876 [0.749,1.025]	0.099
Area (ref. metropolitan)				
Nonmetropolitan	0.932 [0.809,1.073]	0.328	**0.762 [0.654,0.889]**	**0.001**
Not identified	0.964 [0.828,1.123]	0.637	0.958 [0.818,1.122]	0.592
Weekend (ref. weekday)	**0.830 [0.756,0.913]**	**0.000**	**0.675 [0.611,0.746]**	**0.000**
**N**	**7,270**		**6,965**	

	OLS: Duration of time outside (if above 0 min)			
	b [CI 95%]	*P*	b [CI 95%]	*P*
Period (ref. 2003–2010)				
2011–2019	−0.177 [−0.393,−0.038]	0.107	**−0.272 [−0.536,−0.008]**	**0.044**
2020	−0.186 [-0.558,0.187]	0.329	**−0.536 [−0.971,−0.101]**	**0.016**
2021–2023	**−0.375 [−0.668,−0.083]**	**0.012**	**−0.357 [−0.688,−0.026]**	**0.035**
Age (ref. 15)				
16–17	0.053 [−0.057,0.162]	0.345	−0.021 [−0.149,0.108]	0.753
18–19	0.006 [−0.125,0.136]	0.930	−0.097 [−0.243,0.050]	0.197
Region (ref. Northeast)				
Midwest	0.121 [−0.027,0.269]	0.109	0.121 [−0.045,0.288]	0.154
South	**0.158 [0.021,0.296]**	**0.024**	−0.028 [−0.184,0.128]	0.728
West	**0.142 [−0.000,0.285]**	**0.050**	0.103 [−0.060,0.265]	0.215
Area (ref. metropolitan)				
Nonmetropolitan	**0.231 [0.090,0.373]**	**0.001**	0.080 [−0.092,0.252]	0.363
Not identified	−0.035 [−0.182,0.113]	0.647	0.049 [−0.121,0.219]	0.572
Weekend (ref. weekday)	**0.373 [0.281,0.466]**	**0.000**	**0.334 [0.226,0.442]**	**0.000**
**N**	**3,467**		**2,541**	

Notes: Full model results are available in the *SI Appendix*, Table S4.

**Fig. 2.**
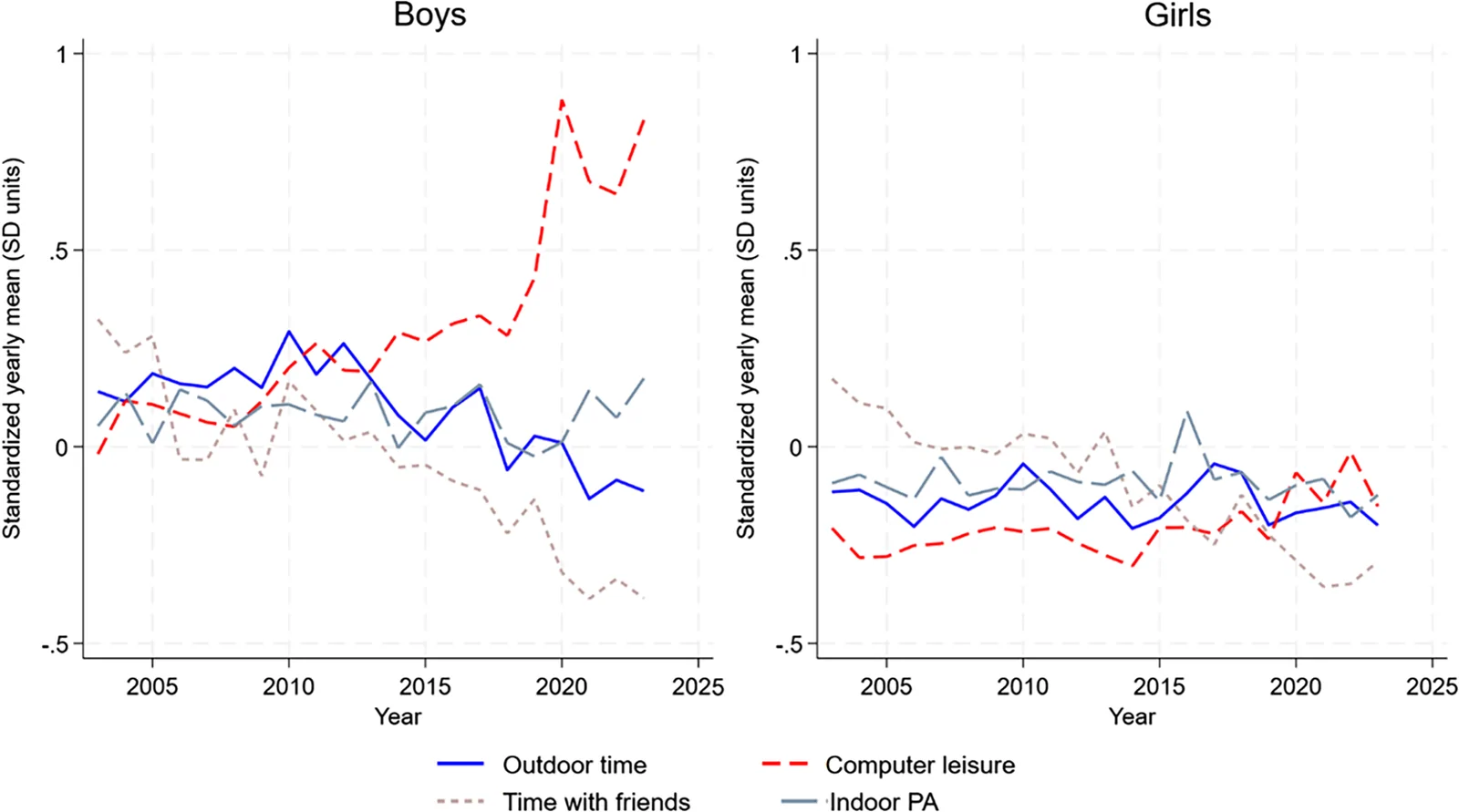
Regression-based estimated yearly means of time spent in selected leisure activities, standardized units (reference year the model: 2003).

Next, we investigate the association between outdoor time and other leisure behaviors, beginning with an analysis of trajectories in leisure activities over time. Fig. 2 shows relative changes in each of the four leisure behaviors. Lines represent predicted yearly means in standardized units from year-fixed-effects models. Throughout the observation period, outdoor time declined for boys, with this decline being accompanied by a substantial increase in computer-based leisure, particularly in 2020 and afterward. There was also a marked reduction in time with a friend for both boys and girls. Indoor physical activity showed limited variability over time and little alignment with outdoor time for either sex.

**Fig. 3.**
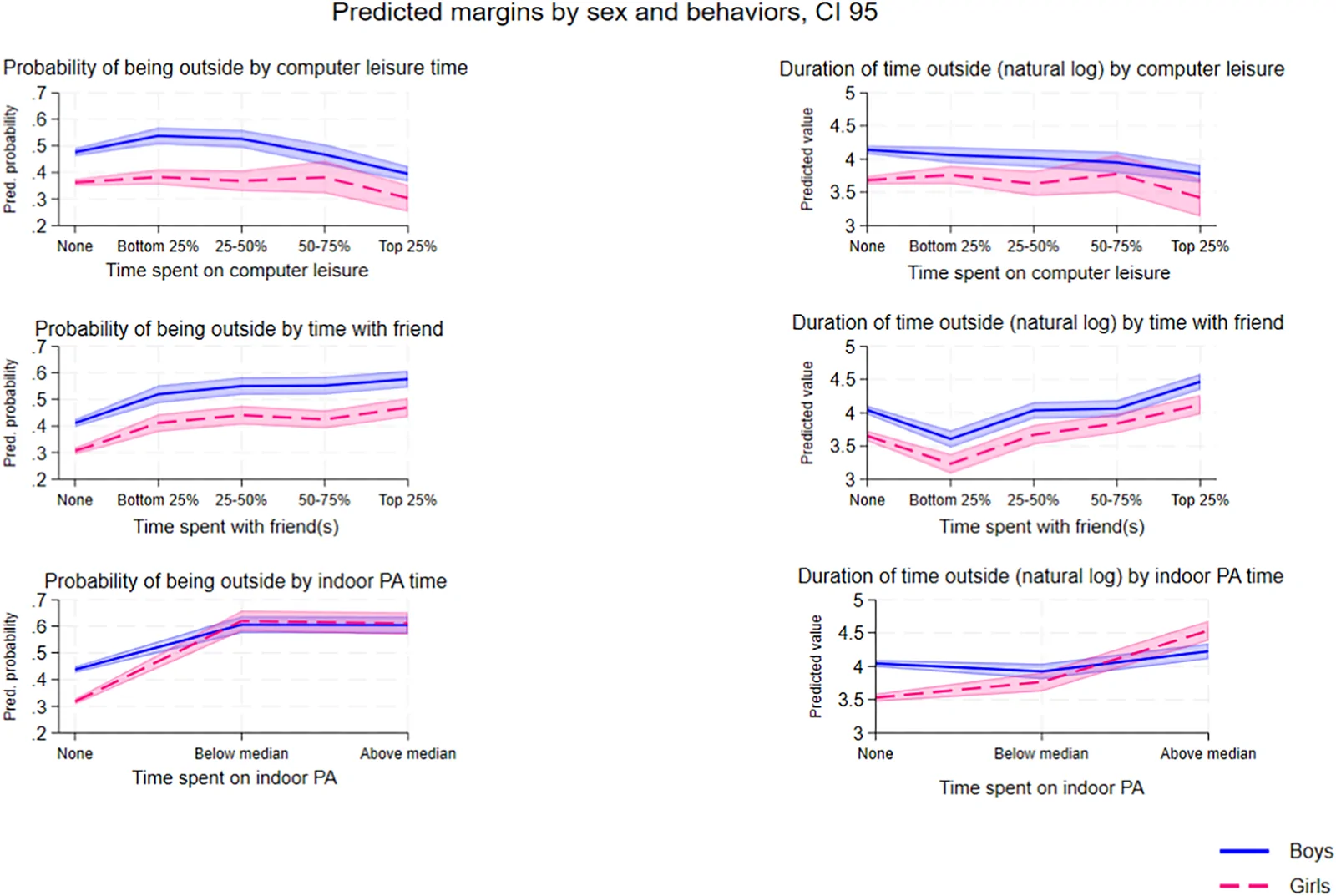
Time spent outdoors (incidence, left panels; duration, right panels) by time spent on computer-based leisure (top), with friends (middle), and on indoor physical activity (bottom), for boys and girls separately; CI 95%.

Adjusted models using individual-level data provide more information on the relationship between different leisure behaviors and outdoor time (Fig. 3). Longer duration of computer-based leisure (top quartile) is associated with a significantly lower probability of going outside and shorter time spent outdoors for boys. The direction of the association is same for girls, but it is not significant. Conversely, more time spent with friends is associated with a higher probability of going out and longer duration of outdoor time for both boys and girls. Adolescents who reported spending more time with a friend than the sample median were more likely to report being outside, and those in the top quartile spent more time outside than peers reporting below-median time with a friend. Indoor physical activity seems complementary rather than a substitute for outdoor time: adolescents reporting any indoor PA were also more likely to engage in outdoor activities. There also seemed to be a particular group of young females who engaged in high levels of indoor PA and spent more time outdoors than other girls but also boys in the sample, suggesting selection.

## Discussion

Over the past 20 y, American adolescent boys have experienced a substantial decline in time spent outdoors, whereas girls have shown little change over the same period. Although girls’ outdoor engagement remained substantially lower than boys’ for most of the study period, this gap gradually narrowed. By the end of the observation period, outdoor time had converged between the sexes, with the difference eventually becoming statistically nonsignificant.

This sex convergence is concerning for several reasons. First, it is not driven by improvements among girls but by marked reductions among boys. Second, males require higher levels of PA to achieve similar cardiovascular benefits as females (35), and derive less mortality reduction from equivalent doses of leisure-time physical activity than women (36). Among adolescents specifically, in both objectively measured and self-reported moderate-to-vigorous physical activity, males show inflection points for cardiorespiratory benefits at higher durations of PA than females (150 min per week of objectively measured PA compared with 90 min per week for females) (37). Given that a large share of daily PA occurs outdoors, reductions in outdoor time may contribute to lower overall PA levels among adolescent boys.

This is a concern because the decrease in outdoor time among American boys does not appear to be offset by changes in indoor PA. First, the duration of indoor PA has not increased over the observation period. Second, it is a selective form of activity, with costs being a frequently reported barrier to access (38). Third, a recent meta-analysis found that indoor PA brings less enjoyment than outdoor activity, possibly reducing long-term adherence (39). Failing to offset reduced outdoor time with sufficient intensity or volume of indoor activity may place boys at elevated risk for suboptimal cardiorespiratory fitness, obesity, and long-term cardiovascular burdens.

Last, childhood engagement with outdoor nature-based activities has been associated with stronger pro-environmental attitudes in adulthood and, for some types of nature experiences, also greater engagement in pro-environmental behaviors (40). The negative consequences of declines in outdoor time may thus extend beyond public health leading also to a reduced support for environmental conservation (41).

Trends in outdoor time reflect broader social, technological, and behavioral changes. The simultaneous rise in computer-based leisure and decline in time spent with a friend likely contribute to the downward trend in outdoor engagement we observe. Among boys in particular, greater time spent on computer use is associated with both a lower likelihood of going outside and shorter outdoor durations. Male heavy-users of computer-based leisure have same levels of outdoor exposure as females. In contrast, spending time with friends is positively associated with outdoor time for both boys and girls. Promoting in-person social interactions may therefore go hand in hand with increasing outdoor engagement, a combination that could yield substantial benefits for young people’s health.

This study has important limitations. First, because of the cross-sectional design, we are not able to monitor how individuals’ behaviors evolve over time. Next, our models do not include any measures of the quality of youth’s outdoor environments. In particular, we are not able to differentiate between being in natural areas versus being in other types of outdoor settings. Furthermore, area profile of the place of residence, such as safety, greenery, and walkability likely influence outdoor time. We do not have information on the characteristics of the area, but we observe an income gradient in the duration of outdoor time. This pattern may reflect previously reported socioeconomic differences in access to and quality of neighborhood outdoor environments, differences in lifestyle, or both. Finally, our measures of outdoor time are imperfect and some of the outdoor time, such as that happening during school hours, might have been missed or misclassified.

Notwithstanding the limitations, our analyses indicate that shifts in digital media use and peer interaction have coincided with and may have contributed to the long-term reduction in outdoor time among American youth. Despite evidence that spending time outdoors may support health, contemporary lifestyle appears to be drawing adolescents away from outdoor settings. The observed decline in outdoor engagement raises important questions about its potential long-term implications, underscoring the need for continued monitoring and targeted interventions to promote equitable access to outdoor environments.

## Materials and Methods

ATUS has a repeated cross-sectional design and provides observational data on individuals’ behaviors in the format of a time-use diary recording all activities reported by an individual for a 24-h period. Diaries are available for sampled individuals in the households included in the Current Population Survey (CPS). Additional background information for ATUS respondents and their household are derived from CPS. We run our analyses on the sample of 14,235 ATUS respondents aged 15 to 19.

Outdoor exposure is operationalized based on location codes indicating where the person is situated. Those include being outside away from home, walking, and cycling (as forms of travel). Some important location codes are ambiguous as regards whether a person is outside or indoors. In particular, the location code for home includes “home and yard” and for school it does not specify whether it is classroom or yard, which does not allow distinguishing between indoor and outdoor time. For this reason, in addition to the location codes, we also use activity codes that indicate being outside, such as lawn and garden maintenance or exterior repairs. We also add any other activities that are explicitly outdoor activities, such as hunting and practicing other outdoor sports, as well as activities that plausibly place a person outdoors such as watching outdoor sports. Some school-based outdoor activities may be underreported, for example if the location is “school” and the activity is not explicitly outdoors (e.g., “talking with friends”), it is not included in outdoor time. However, as exactly same operationalization of outdoor activities is used across all harmonized waves, those omissions should not contribute to systemic differences between individuals or periods. The list of location and activity codes used to calculate outdoor time is provided in the *SI Appendix*, Table S1.

We start analyses by examining the trend in crude mean time spent outdoors by adolescents in the United States. We stratify all analyses by sex because boys and girls differ systematically in their physical activity patterns and use of outdoor environments. Boys typically engage in more moderate-to-vigorous physical activity and exhibit greater variability in activity levels than girls (42, 43). They also consistently spend more time outdoors, including in outdoor sports (44–46), tend to prefer movement patterns that are better suited to outdoor settings (47), and use outdoor spaces differently than girls (33).

For trend analyses we use ATUS population weights for descriptive analyses along with providing nonweighted estimates. We exclude observations from 2020 because of disruptions to data collection as well as distortions to individual’s time use that were due to COVID-19. However, 2020 is retained in inferential analyses as a separate time period.

Next, as the distribution of outdoor time is skewed with a high number of zeros, we use a hurdle model approach. We first model the incidence (whether a person was outdoors) using logistic regression. Next, we model duration of total time outside among those who reported any time outdoors, using a linear regression and natural log of duration time to correct for the nonnormal distribution. In the models, we pool years into periods to boost sample size: 2003–2010, 2011–2019, 2020, and 2021–2023. The periods reflect the characteristics of each decade, with the first period 2003–2010 being mostly pre–social media era, the time between 2011–2019 is the period of major increase in Internet use, 2020 represent the time of the COVID-19 related disruption to usual routines, and 2021–2023 reflects the “post pandemic” reality. We also include detailed age groups (15–19), intended to capture differences related to mobility and independence from parents (e.g., obtaining a driving license is possible from age 16 and allows for greater transport independence), as well as differences across levels of schooling. All models are stratified by sex.

The models are adjusted for sociodemographic and environmental factors. Environmental conditions are represented by the US region: Northeast, Midwest, South, West (the list of states in each region is available in the *SI Appendix*, Table S2), type of municipality (metropolitan, nonmetropolitan), and season for which the diary is available (spring, summer, autumn, winter), all of which may influence how much time is spent outdoors. We also include a dummy for whether it is a weekday or a weekend. Sociodemographic conditions include race and income groups. Individuals of different races and income groups may have access to different types of outdoor environments, in particular in the terms of access to and use of natural areas (48, 49). In ATUS, monthly income is given per family, so we also account for household size, which is also a proxy for household composition. Household income is available starting from 2010, we therefore retain missing values for income as separate category in models using years between 2003–2023 and provide additional models only for the period 2010–2023 in the *SI Appendix*, as income effects are more robust for this period. Last, we include respondents’ disability status (binary), as having a disability may limit opportunities for outdoor recreation.

In the second step, we investigate the associations between time spent outdoors and young people’s leisure behaviors potentially linked with trends in outdoor use: computer use for gaming and other leisure, indoor physical activity, and time spent with friend(s). We estimate time spent on those activities based on the behaviors reported in the diaries. Indoor physical activity is any physical activity which is not explicitly outdoors (e.g., skiing, hiking) and for which the location code is not “outdoors.” The list of activities is given in the *SI Appendix*, Table S3. Computer-based leisure and gaming include two activity codes of exactly these names. We do not look at smartphone use because it was not consistently measured across all waves and because smartphone use, unlike computer use, is usually not mutually exclusive with being outside. We also do not examine television viewing, as it appears to have become less central in young people’s time-use patterns, following a decline in television consumption alongside the expansion of online leisure activities (25, 50). Finally, total time spent with a friend is operationalized as the sum of all instances for which a copresence of a friend was reported. The data does not indicate whether one or more friends were present during an episode of activity, so time with a friend is an indicator of the duration of social (nonsolitary) time, not the extensiveness of one’s social contacts.

First, to jointly analyze the trends for selected leisure behaviors, we standardize measures of duration of each behavior across both sexes for comparability between boys and girls, and use a set of linear regression models with cluster SE and year fixed effects (with 2003 as reference year) to obtain predicted annual means of each type of leisure activity. We visualize the joint trends by plotting the predicted yearly means stratified by sex.

Next, to understand the relationship between different leisure behaviors and outdoor time at an individual level, we use logit (for incidence of outdoor time) and linear (for duration of outdoor time among those who go out) models, fully adjusted for the same set of confounders as in the earlier hurdle models. For individual-level analyses, we collapse leisure behaviors into categorical measures to facilitate interpretation and allow for nonlinear effects. All behaviors have high shares of zeros (nonparticipation), so nonparticipation is included as a separate category for each of them. Durations greater than zero are recoded into quartiles based on the distribution of each type of activity in the sample, because the relationship between different leisure activities and outdoor time may be nonlinear. For indoor PA, because of its overall low incidence, we recode the values into below-median and above-median categories to preserve cell size. In these models, instead of splitting the sample, we pool data for boys and girls and add interaction term between sex and each type of leisure behavior to examine sex differences in the effects of each behavior.

## Supplementary Material

Appendix 01 (PDF)

## Data Availability

The American Time Use Survey (ATUS) microdata are publicly available for download from the Bureau of Labor Statistics (BLS) website (https://www.bls.gov/tus/data.htm) (34). The aggregated day-level data and the syntax used in this study are available at https://github.com/EwaJarosz/ATUS-2003-2023 (51).
