# Supplementary material for: Changes in American adolescents’ outdoor time over two decades
Source: Proc Natl Acad Sci U S A. 2026 Oct 26;123(44):e2605134123. doi: 10.1073/pnas.2605134123 (PMC13732205; doi:10.1073/pnas.2605134123)
Supplement: Supplementary file 1 [file pnas.2605134123.sapp.pdf]

PNAS

Supporting Information for

Changes in American Adolescents' Outdoor Time Over Two Decades

Ewa Jarosz*[1], Vidar Ulset[2], Vegard Skirbekk[1,3]
1 Department of Psychology, University of Oslo, Oslo, Norway
2 Norwegian School of Sport Sciences, Oslo, Norway
3 Norwegian Institute of Public Health, Centre for Fertility and Health, Oslo, Norway

Corresponding author: Ewa Jarosz
Email: ewa.jarosz@psykologi.uio.no

This PDF file includes:

Figures S1
Tables S1 to S5
Bureau of Labor Statistics, ATUS data: Interview data dictionary 2003-2023
Bureau of Labor Statistics, ATUS data: Activity Lexicon 2003-2023

Supporting Information

Figures

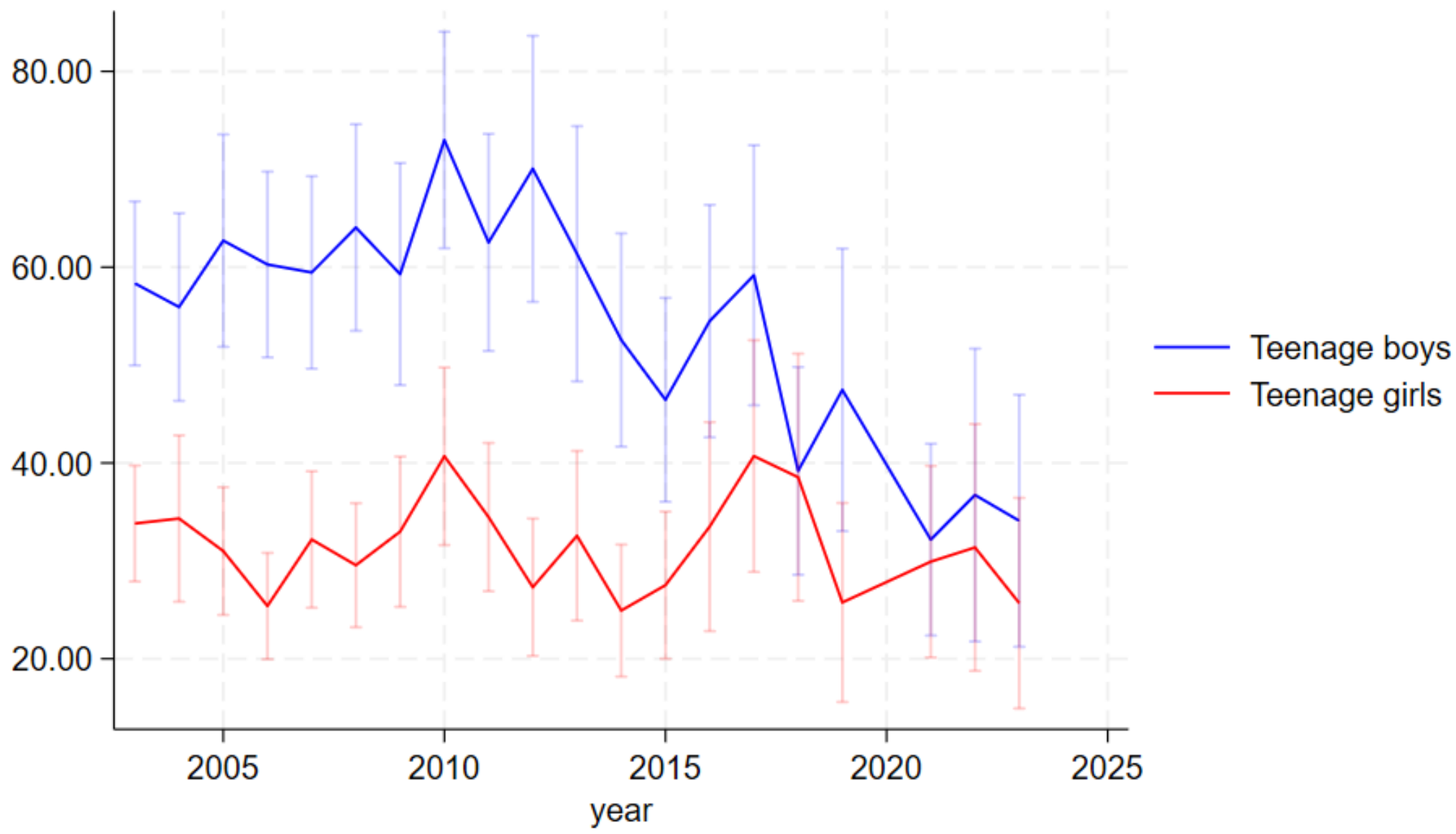

Fig. S1. Non-smoothed and unweighted trend: mean time spent outdoors by sex and year, CI 95%.

Tables

Table S1. List of ATUS location and activity codes used to compute amount of time spent outdoors.

Location codes (ATUS original codes and labels)	Activity codes (ATUS original codes and labels)
9 Outdoors away from home<br>14 Walking (as means of transport)<br>17 Bicycle (as means of transport)	020401 Exterior cleaning<br>020402 Exterior repair, improvements, & decoration<br>020499 Exterior maintenance, repair & decoration, n.e.c.<br>020501 Lawn, garden, and houseplant care<br>020502 Ponds, pools, and hot tubs<br>020599 Lawn and garden, n.e.c.*<br>130102 Playing baseball<br>130104 Biking<br>130106 Boating<br>130108 Climbing, spelunking, caving<br>130112 Fishing<br>130113 Playing football<br>130114 Golfing<br>130110 Participating in equestrian sports<br>130116 Hiking<br>130118 Hunting<br>130120 Playing racquet sports<br>130121 Participating in rodeo competitions<br>130122 Rollerblading<br>130123 Playing rugby<br>130124 Running<br>130125 Skiing, ice skating, snowboarding<br>130126 Playing soccer<br>130129 Vehicle touring/racing<br>130131 Walking<br>130132 Participating in water sports<br>130204 Watching biking<br>130206 Watching boating<br>130208 Watching climbing, spelunking, caving<br>130210 Watching equestrian sports<br>130212 Watching fishing<br>130213 Watching football<br>130214 Watching golfing<br>130218 Watching racquet sports<br>130219 Watching rodeo competitions<br>130220 Watching rollerblading<br>130221 Watching rugby<br>130222 Watching running<br>130223 Watching skiing, ice skating, snowboarding<br>130224 Watching soccer<br>130225 Watching softball<br>130226 Watching vehicle touring/racing<br>130228 Watching walking<br>130229 Watching water sports<br>150301 Building houses, wildlife sites, & other structures

Table S2. States included within each geographic region.

Northeast	Midwest (North Central)	South	West
Connecticut	Illinois	Alabama	Alaska
Maine	Indiana	Arkansas	Arizona
Massachusetts	Iowa	Delaware	California
New Hampshire	Kansas	District of Columbia	Colorado
New Jersey	Michigan	Florida	Hawaii
New York	Minnesota	Georgia	Idaho
Pennsylvania	Missouri	Kentucky	Montana
Rhode Island	Nebraska	Louisiana	Nevada
Vermont	North Dakota	Maryland	New Mexico
	Ohio	Mississippi	Oregon
	South Dakota	North Carolina	Utah
	Wisconsin	Oklahoma	Washington
		South Carolina	Wyoming
		Tennessee	
		Texas	
		Virginia	
		West Virginia	

Table S3. List of ATUS location and activity codes used to compute amount of time spent doing indoor physical activity.

Location codes (ATUS original codes and labels)	Activity codes (ATUS original codes and labels)
Location NOT equal to:<br>9 Outdoors away from home	130101 Doing aerobics<br>130103 Playing basketball<br>130105 Playing billiards<br>130107 Bowling<br>130109 Dancing<br>130111 Fencing<br>130115 Doing gymnastics<br>130117 Playing hockey<br>130119 Participating in martial arts<br>130120 Playing racquet sports<br>130124 Running<br>130127 Softball<br>130128 Using cardiovascular equipment<br>130129 Vehicle touring/racing<br>130130 Playing volleyball<br>130131 Walking<br>130132 Participating in water sports<br>130133 Weightlifting/strength training<br>130134 Working out, unspecified<br>130135 Wrestling<br>130136 Doing yoga<br>130199 Playing sports n.e.c.*<br>139999 Sports, exercise, & recreation, n.e.c.*<br>050203 Sports and exercise as part of job

Note: Some activities may occur both indoors and outdoors and therefore appear in both Tables S1 and S3. In these cases, they are coded as outdoor activity if the location code is “outdoors” and as indoor physical activity if the location code is NOT “outdoors” (e.g., walking can be performed both outdoors and indoors, including “mall walking” or walking on a treadmill in a fitness center).

Table S4. Hurdle model of outdoor time (incidence and duration).

	Logit: Incidence (any time outside)				OLS: Duration of time outside (if above 0 mins)			
	Boys		Girls		Boys		Girls	
	OR [CI 95%]	p	OR [CI 95%]	p	b [CI 95%]	p	b [CI 95%]	p
Period (ref. 2003-2010)								
2011-2019	**0.702 [0.551,0.895]**	**0.004**	0.935 [0.728,1.201]	0.597	-0.177 [-0.393,-0.038]	0.107	**-0.272 [-0.536,-0.008]**	**0.044**
2020	**0.437 [0.302,0.633]**	**0.000**	0.866 [0.579,1.294]	0.482	-0.186 [-0.558,0.187]	0.329	**-0.536 [-0.971,-0.101]**	**0.016**
2021-2023	**0.508 [0.375,0.688]**	**0.000**	0.886 [0.651,1.207]	0.443	**-0.375 [-0.668,-0.083]**	**0.012**	**-0.357 [-0.688,-0.026]**	**0.035**
Age (ref. 15)								
16-17	**0.645 [0.573,0.726]**	**0.000**	**0.684 [0.605,0.773]**	**0.000**	0.053 [-0.057,0.162]	0.345	-0.021 [-0.149,0.108]	0.753
18-19	**0.497 [0.435,0.569]**	**0.000**	**0.627 [0.546,0.719]**	**0.000**	0.006 [-0.125,0.136]	0.930	-0.097 [-0.243,0.050]	0.197
Season (ref. spring)								
Summer	**1.186 [1.037,1.356]**	**0.013**	**1.290 [1.124,1.480]**	**0.000**	**0.349 [0.223,0.475]**	**0.000**	**0.392 [0.247,-0.537]**	**0.000**
Fall	0.918 [0.802,1.050]	0.210	0.946 [0.818,1.093]	0.448	0.001 [-0.129,0.131]	0.990	-0.077 [-0.233,-0.079]	0.332
Winter	**0.620 [0.543,0.707]**	**0.000**	**0.673 [0.584,0.775]**	**0.000**	**-0.337 [-0.471,-0.203]**	**0.000**	**-0.259 [-0.417,-0.102]**	**0.001**
Race (ref. white)								
Black	**1.220 [1.050,1.417]**	**0.009**	**1.212 [1.037,1.416]**	**0.016**	**-0.548 [-0.692,-0.404]**	**0.000**	**-0.546 [-0.711,-0.381]**	**0.000**
Native American	**0.585 [0.349,0.979]**	**0.041**	1.141 [0.707,1.841]	0.589	-0.132 [-0.693,0.428]	0.644	-0.262 [-0.777,0.253]	0.318
Asian	**0.756 [0.597,0.959]**	**0.021**	1.119 [0.874,1.432]	0.372	**-0.496 [-0.739,-0.253]**	**0.000**	**-0.275 [-0.533,-0.017]**	**0.037**
Other	0.988 [0.754,1.295]	0.930	1.142 [0.867,1.504]	0.344	-0.007 [-0.269,0.255]	0.958	-0.164 [-0.454,0.126]	0.268
Region (ref. Northeast)								
Midwest	**0.767 [0.659,0.893]**	**0.001**	**0.729 [0.623,0.854]**	**0.000**	0.121 [-0.027,0.269]	0.109	0.121 [-0.045,0.288]	0.154
South	**0.756 [0.656,0.872]**	**0.000**	**0.630 [0.543,0.731]**	**0.000**	**0.158 [0.021,0.296]**	**0.024**	-0.028 [-0.184,0.128]	0.728
West	1.042 [0.896,1.211]	0.593	0.876 [0.749,1.025]	0.099	0.142 [-0.000,0.285]	0.050	0.103 [-0.060,0.265]	0.215
Area (ref. metropolitan)								
Non-metropolitan	0.932 [0.809,1.073]	0.328	**0.762 [0.654,0.889]**	**0.001**	**0.231 [0.090,0.373]**	**0.001**	0.080 [-0.092,0.252]	0.363
Not identified	0.964 [0.828,1.123]	0.637	0.958 [0.818,1.122]	0.592	-0.035 [-0.182,0.113]	0.647	0.049 [-0.121,0.219]	0.572

Family income quartile (ref. bottom 25%)								
25-50%	0.982 [0.808,1.194]	0.855	0.998 [0.810,1.230]	0.987	0.064 [-0.129,0.257]	0.518	-0.060 [-0.287,0.168]	0.608
50-75%	0.971 [0.795,1.186]	0.774	1.013 [0.818,1.255]	0.907	**0.228 [0.029,0.428]**	**0.025**	0.190 [-0.042,0.421]	0.109
Top 25%	1.000 [0.822,1.216]	0.999	1.024 [0.835,1.256]	0.817	**0.217 [0.022,0.413]**	**0.030**	**0.236 [0.015,0.457]**	**0.036**
Missing	0.702 [0.521,0.947]	0.020	1.144 [0.836,1.565]	0.401	0.058 [-0.220,0.336]	0.681	-0.415 [-0.746,-0.084]	0.014
Household size (ref. 3 persons)								
1 person (respondent)	1.331 [0.785,2.257]	0.289	1.551 [0.837,2.876]	0.163	0.133 [-0.389,0.655]	0.618	-0.433 [-1.069,0.204]	0.183
2 persons	1.099 [0.919,1.316]	0.301	0.940 [0.774,1.142]	0.533	0.014 [-0.163,0.191]	0.874	0.082 [-0.131,0.294]	0.450
4 persons	1.056 [0.929,1.201]	0.401	1.094 [0.955,1.254]	0.195	0.087 [-0.040,0.214]	0.178	0.012 [-0.136,0.160]	0.877
5 or more persons	1.111 [0.977,1.262]	0.108	1.141 [0.995,1.307]	0.058	0.106 [-0.020,0.232]	0.098	0.004 [-0.143,0.151]	0.958
Disability (ref. no)								
Yes	0.853 [0.627,1.161]	0.312	0.906 [0.625,1.312]	0.600	-0.228 [-0.540,0.084]	0.151	-0.138 [-0.545,0.269]	0.506
Missing information	1.057 [0.886,1.261]	0.536	**0.827 [0.686,0.998]**	**0.047**	-0.135 [-0.309,0.038]	0.126	0.088 [-0.111,0.288]	0.385
Type of day (ref. weekday)								
Weekend	**0.830 [0.756,0.913]**	**0.000**	**0.675 [0.611,0.746]**	**0.000**	**0.373 [0.281,0.466]**	**0.000**	**0.334 [0.226,0.442]**	**0.000**
Intercept	2.633 [1.910,3.631]	0.000	1.276 [0.919,1.786]	0.155	3.756 [3.462,4.049]	0.000	3.811 [3.460,4.163]	0.000
**N**	**7,270**		**6,965**		**3,467**		**2,541**	

Table S5. Model 1 for years 2010-2023 (the period for which the information on income was available)

	Incidence (logit) of outdoor time				Duration (OLS) of outdoor time			
	BOYS		GIRLS		BOYS		GIRLS	
	OR [CI]	p	OR [CI]	p	b [CI]	p	b [CI]	p
Period (ref. 2010-2012)								
2013-2015	0.853 [0.713,1.021]	0.084	0.865 [0.714,1.048]	0.140	-0.186 [-0.358,-0.013]	0.035	-0.138 [-0.343,0.067]	0.187
2016-2019	0.757 [0.632,0.905]	0.002	1.015 [0.839,1.229]	0.878	-0.213 [-0.387,-0.038]	0.017	-0.074 [-0.274,0.127]	0.470
2020	0.516 [0.377,0.706]	0.000	0.864 [0.609,1.226]	0.414	-0.077 [-0.408,0.255]	0.651	-0.313 [-0.689,0.063]	0.102
2021-2023	0.612 [0.485,0.773]	0.000	0.912 [0.719,1.156]	0.447	-0.355 [-0.596,-0.114]	0.004	-0.207 [-0.461,0.047]	0.110
Age (ref. 15)								
16-17	0.683 [0.577,0.809]	0.000	0.758 [0.635,0.905]	0.002	0.042 [-0.117,0.200]	0.606	-0.096 [-0.280,0.088]	0.307
18-19	0.589 [0.489,0.710]	0.000	0.727 [0.599,0.883]	0.001	-0.055 [-0.235,0.125]	0.548	-0.051 [-0.255,0.153]	0.627
Season (ref. spring)								
Summer	1.128 [0.936,1.360]	0.205	1.202 [0.991,1.459]	0.062	0.391 [0.213,0.569]	0.000	0.431 [0.230,0.633]	0.000
Autumn	0.932 [0.769,1.129]	0.470	0.897 [0.731,1.100]	0.297	0.028 [-0.157,0.213]	0.764	-0.011 [-0.229,0.206]	0.919
Winter	0.589 [0.490,0.708]	0.000	0.691 [0.566,0.843]	0.000	-0.167 [-0.354,0.020]	0.081	-0.254 [-0.471,-0.038]	0.021
Race (ref. white)								
Black	1.125 [0.908,1.395]	0.280	1.023 [0.820,1.276]	0.843	-0.468 [-0.677,-0.258]	0.000	-0.440 [-0.676,-0.203]	0.000
Native American	0.719 [0.342,1.515]	0.386	0.950 [0.491,1.837]	0.879	-0.519 [-1.310,0.272]	0.198	-0.161 [-0.873,0.551]	0.658
Asian	0.690 [0.504,0.945]	0.021	1.080 [0.791,1.474]	0.628	-0.446 [-0.776,-0.117]	0.008	-0.394 [-0.717,-0.071]	0.017
Other	1.114 [0.774,1.602]	0.562	1.075 [0.729,1.586]	0.715	-0.260 [-0.609,0.089]	0.144	0.074 [-0.336,0.484]	0.723
Region (ref. Northeast)								
Midwest	0.687 [0.552,0.855]	0.001	0.702 [0.560,0.882]	0.002	0.022 [-0.190,0.234]	0.836	0.031 [-0.203,0.265]	0.796
South	0.713 [0.583,0.873]	0.001	0.577 [0.467,0.714]	0.000	0.087 [-0.107,0.281	0.378	-0.035 [-0.254,0.183]	0.753
West	0.951 [0.768,1.178]	0.646	0.791 [0.631,0.990]	0.040	0.091 [-0.110,0.292]	0.376	0.118 [-0.109,0.344]	0.309
Area (ref. metropolitan)								
Non-metropolitan	0.993 [0.821,1.200]	0.940	0.777 [0.632,0.955]	0.016	0.244 [0.056,0.432]	0.011	-0.019 [-0.246,0.209]	0.873
Not identified	0.973 [0.478,1.981]	0.940	0.882 [0.437,1.780]	0.727	-0.184 [-0.888,0.520]	0.608	0.249 [-0.519,1.016]	0.525
Family income (ref. bottom 25%)								
25-50%	0.962 [0.791,1.171]	0.703	0.977 [0.793,1.204]	0.829	0.094 [-0.100,0.288]	0.341	-0.049 [-0.274,0.176]	0.669
50-75%	0.952 [0.778,1.166]	0.634	0.993 [0.801,1.233]	0.952	0.268 [0.066,0.470]	0.009	0.191 [-0.040,0.422]	0.105
Top 25%	0.980 [0.801,1.199]	0.842	0.979 [0.794,1.207]	0.842	0.272 [0.070,0.473]	0.008	0.241 [0.017,0.465]	0.035
Missing	1.104 [0.742,1.643]	0.626	1.056 [0.674,1.655]	0.811	0.195 [-0.189,0.579]	0.319	0.068 [-0.415,0.550]	0.784
Number of people living in respondent's household (ref. 3)								
1	0.824 [0.363,1.868]	0.643	0.888[0.354,2.226]	0.800	0.589 [-0.289,1.466]	0.188	-0.737 [-1.765,0.292]	0.160
2	1.016 [0.795,1.300]	0.897	0.813 [0.615,1.074]	0.145	0.073 [-0.174,0.321]	0.561	0.203 [-0.103,0.509]	0.194
4	1.102 [0.920,1.321]	0.293	1.063 [0.877,1.289]	0.534	0.076 [-0.103,0.256]	0.405	0.060 [-0.147,0.268]	0.568

5 or more	1.120 [0.934,1.343]	0.220	1.136 [0.939,1.374]	0.191	0.090 [-0.089,0.269]	0.325	0.004 [-0.199,0.208]	0.966
Disability status (ref. no)								
Yes	0.826 [0.597,1.144]	0.251	0.789 [0.523,1.191]	0.260	-0.164 [-0.491,0.163]	0.325	0.010 [-0.447,0.467]	0.966
Intercept	2.054[1.496,2.818]	0.000	1.119 [0.804, 1.558]	0.505	3.892 [3.594,4.190]	0.000	3.757 [3.415,4.099]	0.000
**N**	**3,731**		**3,508**		**1,732**		**1,263**	

American Time Use Survey (ATUS) Data Dictionary:

2003-23 Interview Data

Variables collected in ATUS

June 2024

The American Time Use Survey (ATUS) is sponsored by the Bureau of Labor Statistics
and conducted by the U.S. Census Bureau.

Important Notice about 2020 ATUS data collection

Data represent only 10 months of 2020

ATUS data collected in 2020 are not representative of the full year because they were collected for only 10 months. ATUS data were not collected about the days March 18, 2020 to May 9, 2020, which coincided with a time when most residents of the United States were encouraged to stay home to prevent the spread of COVID-19. The ATUS does not have information on how people spent their time during this period when data were not collected.

Researchers can use the variable TUDIARYDATE to identify the date about which respondents reported their use of time. The data files contain time diary data collected before and after the temporary shutdown in ATUS operations, but there are no values of TUDIARYDATE for March 18, 2020 to May 9, 2020.

The method used to develop statistical weights for the ATUS data was modified. Time-use estimates produced with the 2020 statistical weights represent only the days before and after the pandemic-related shutdown in data collection. That is, they will represent the periods January 1st to March 17th and May 10th to December 31st.

For more information about the effects of the pandemic on the collection and processing of ATUS data, see https://www.bls.gov/tus/notices/2021/covid19.htm

Important Information about the ATUS Data Dictionary

Introduction

The American Time Use Survey (ATUS) is sponsored by the Bureau of Labor Statistics and conducted by the U.S. Census Bureau. The purpose of this document is to provide information about the variables available on six multi-year ATUS data files: the Respondent file, the Roster file, the Activity file, the Who file, the Eldercare Respondent file, and the Activity Summary file. These files contain information collected and assigned in the 2003 to 2023 ATUS interviews. This data dictionary differs from individual year data dictionaries because it describes files that have data from multiple years.

This data dictionary lists all the variables available on these multi-year files and their valid values. It also provides directions on how to read the data dictionary.

Three additional data dictionaries describe other ATUS data files:

- ATUS-CPS Data Dictionary: Describes the variables available on the ATUS-CPS file as well as some variables on the Activity Summary file. The ATUS-CPS file contains data from the Current Population Survey (CPS) for persons selected to be surveyed for the ATUS and for members of their households. (The information on the ATUS-CPS file was collected two to five months before the ATUS interview and in some cases was out of date at the time the ATUS was conducted.) This dictionary is available for both multi-year and single-year ATUS-CPS data files.
- Survey Methodology Data Dictionary: Describes the variables available on the Case History file and the Call History file. This data dictionary is available for single-year Case History and Call History files and there is a multi-year Survey Methods Data Dictionary describing the variables available on the 2005-23 Case History file. There is no multi-year file for the Call History files.
- Trips Data Dictionary: Starting in 2011, the trips questions were replaced by the eldercare questions. The data dictionary describes the variables on the Trips files, which are available for the years, 2005-10.This data dictionary is only available for single-year Trips files because there are no multi-year Trips files.

These additional single- and multi-year data dictionaries are available on the ATUS Web site at www.bls.gov/tus/dictionaries.htm.

ATUS Interview Data Files

The following six multi-year data files include data available from the ATUS interviews.

1. 2003-23 ATUS Respondent File

This file contains case-specific variables collected in all currently available years of the ATUS (that is, variables for which there is one value for each respondent). These include, for example, labor force and earnings information, total time providing secondary childcare, and ATUS statistical weights. The ATUS statistical weights variable (TUFNWGTP) was developed using the 2006 weighting method. For more information about ATUS statistical weights, why researchers should use them, and details about the ATUS weighting methods, see the ATUS User's Guide (www.bls.gov/tus/atususersguide.pdf).

There is one record for each ATUS respondent.

Below is a simplified example. The TUCASEID identifies each household, and TULINENO identifies each individual within the household. The example contains responses from five individuals; note that the respondent always has TULINENO=1. In the example, each respondent has corresponding values denoting school enrollment (TESCHENR), labor force status (TELFS), and total nonwork-related time spent alone (TRTALONE). The actual ATUS Respondent file contains many more variables as well as many more lines.

TUCASEID	TULINENO	TESCHENR	TELFS	TRTALONE
20230101020230	1	1	1	40
20230101020231	1	1	1	350
20230101020232	1	1	5	0
20230101020233	1	2	5	556
20230101020234	1	1	4	100

2. 2003-23 ATUS Roster File

This file contains information on the age, sex, and each household member's relationship to the ATUS respondent. The same information is also included for the respondent's own nonhousehold children under 18.

There is one record for each individual in the respondent's household (including the respondent's own nonhousehold children under 18).

A simplified example appears below. The TUCASEID identifies each household, and the TULINENO identifies each individual in the household. In the example below, TUCASEID 20230101020230 has three persons residing in the household, and TUCASEID 20230101020231 and TUCASEID 20230101020232 each have one person. The actual ATUS Roster file contains more variables and many additional lines.

TUCASEID	TULINENO	TERRP	TESEX	TEAGE
20230101020230	1	18	2	42
20230101020230	2	20	1	45
20230101020230	3	22	1	11
20230101020231	1	18	1	65
20230101020232	1	18	2	21

3. 2003-23 ATUS Activity File

This file includes activity-level information collected in ATUS, including activity code, location, duration, activity start and stop times, and whether respondents had a child under 13 in their care during the activity. Location (or "where") information is not collected for some selected activities (such as sleeping and grooming); a value that indicates the activity was "out of universe" for the "where" question (-1) is filled in these situations.
There is one record for each activity.

A simplified example of the ATUS Activity file appears below. This is an illustration of part of one respondent's day. Because only one person is interviewed per household, each TUCASEID on the Activity file identifies a respondent. Each activity is identified by an activity number (TUACTIVITY_N). The ATUS Activity file contains more variables describing each activity as well as many more lines than does the example below. Due to changes made to the activity lexicons in the early years of the ATUS, activity codes appear on the 2003-23 Activity file that do not appear on the single-year Activity files. A new 2003-23 Activity Coding lexicon is available at https://www.bls.gov/tus/lexicons/lexiconnoex0323.pdf with codes that appear on the 2003-23 Activity file.

TUCASEID	TUACTIVITY_N	TUSTARTTIM	TUSTOPTIME
20230101020230	1	04:00:00	07:00:00
20230101020230	2	07:00:00	07:30:00
20230101020230	3	07:30:00	08:00:00
20230101020230	4	08:00:00	12:00:00
20230101020230	5	12:00:00	13:30:00
20230101020230	6	13:30:00	17:30:00
20230101020230	7	17:30:00	18:00:00
20230101020230	8	18:00:00	19:00:00
20230101020230	9	19:00:00	21:00:00
20230101020230	10	21:00:00	04:00:00

4. 2003-23 ATUS Who File

This file includes codes that indicate who was present during each activity.

There is one record for each "who" code reported. Therefore, there will be one record for activities done alone and multiple records for activities with multiple people present. For some activities, no "who" codes are collected (such as sleeping and grooming in all years, and working in 2003-09); a value that indicates the activity was "out of universe" for the "who" question (-1) is filled in these situations.

A simplified example appears below. In the first activity (TUACTIVITY_N = 1), no "who" code information was collected because of the associated activity code. Only one person was with the respondent during the second activity, so there is one line for TUACTIVITY_N = 2. Three people were with the respondent during the third activity, so there are three lines for TUACTIVITY_N = 3. Two of those (TUWHO_CODE = 20 and 22) are members of the respondent's household and can be linked to the Roster file using TUCASEID and TULINENO. The third (TUWHO_CODE = 51) is not a member of the respondent's household and thus does not have a positive value for TULINENO. The actual ATUS Who file contains more variables for each line as well as many additional lines than the example below.

TUCASEID	TUACTIVITY_N	TUWHO_CODE	TULINENO
20230101020230	1	-1	-1
20230101020230	2	22	3
20230101020230	3	20	2
20230101020230	3	22	3
20230101020230	3	51	-1

5. 2011-23 ATUS Eldercare Roster File (introduced in 2011)

The ATUS Eldercare Roster file contains information about people for whom the respondent provided care. If the respondent indicated that she had provided eldercare more than once during the past 3 to 4 months, additional information about each eldercare recipient is collected. (The time frame varied slightly by respondent because the question asked about care provided between the 1st of a reference month and the interview day.) There is one record for each recipient, up to a maximum of 5 records for each respondent. Information about the relationship of the recipient to the respondent, the age of the recipient, and the duration that care had been provided appear on the file.

A simplified example of the ATUS Eldercare Roster file appears below. The TUCASEID identifies each respondent providing eldercare, and the TULINENO identifies recipients in the household. A value of -1 for TULINENO indicates that the eldercare recipient does not live in the household. In the example below, TUCASEID 20230101020230 provided care to two persons not living in the household, TUCASEID 20230101020231 provided care to one person, who does live in the household, and TUCASEID 20230101020235 and TUCASEID 20230101020238 each provided care to one person. The actual ATUS Eldercare Roster file contains more variables and many additional lines.

TUCASEID	TULINENO	TEELWHO	TEAGE_EC	TEELDUR
20230101020230	-1	33	76	4
20230101020230	-1	34	80	4
20230101020231	2	20	72	4
20230101020235	-1	46	88	3
20230101020238	-1	55	65	2

6. 2003-23 ATUS Activity Summary File

The ATUS Activity Summary file contains information about the total number of minutes each respondent spent doing each activity. The file also includes selected variables from the ATUS Respondent, ATUS Roster, and ATUS-CPS files. **The Activity Summary file contains variables not described in this data dictionary. Variables beginning with a lower-case "t" correspond to specific activity codes; definitions for each activity code can be found in the 2003-23 Activity Lexicon (**https://www.bls.gov/tus/lexicons/lexiconnoex0323.pdf)

There is one record for each ATUS respondent.

A simplified example of the ATUS Activity Summary file appears below. The variable TUCASEID is the unique identifier for each respondent and the variable TEAGE, which also appears on the ATUS Roster file, shows each respondent's age. The variable t010101 contains the total number of minutes each respondent spent doing activity 010101, "sleeping"; the variable t010102 contains the total number of minutes each respondent spent doing activity 010102, "sleeplessness."

The ATUS Activity Summary file contains more variables describing each activity as well as many more lines than the example below.

TUCASEID	TEAGE	t010101	t010102
20230101020230	26	480	0
20230101020231	53	430	30
20230101020232	76	457	0
20230101020233	16	600	0

Valid Values

Each variable has a number of valid values or a range of valid values. For example, the variable TESEX has two valid values: 1 for male and 2 for female. The variable TEAGE, on the other hand, has a range of valid values – any entry between 0 and 85 (except 81 through 84) is considered valid. Individual valid values or a range of valid values are listed under each variable in the data dictionary. A few variables have so many valid values that they are not included in the data dictionary; instead, they are provided in an appendix or a separate document. (References to these are included as a "Note" under the relevant variables in the data dictionary.) One example of such a variable is TEIO1ICD, which identifies the industry code of the respondent's main job.

Many ATUS variables have the following possible valid values:

Value	Description
-1	Blank
-2	Don't know
-3	Refused

Because so many variables have these possible values, they are not shown as valid entries for each variable.

TUCASEID, the primary identification number for ATUS, does not have either a list of valid values or a range of valid values.

ATUS Naming Conventions and Definitions

ATUS variables are named according to specified rules. Variables with a first character of “T” (for time use) were collected or created through the ATUS interview. Variables with any other first character (most often “P”, “G”, or “H”) were collected or created through the final CPS interview (conducted two to five months prior to the ATUS interview). All of the variables on the ATUS interview data files described in this dictionary begin with “T.”

The second and third characters of the name identify the type of variable, and the remaining characters consist of a descriptive name. The rules regarding the first two or three characters are described in the table below (note that the variables on the Activity Summary file that start with a lowercase “t” do not follow these rules):

Abbreviation	Variable Type	Definition
U	Unedited Variable	An unedited variable generally is produced by the Computer Assisted Telephone Interview (CATI) instrument, either collected or assigned during the interview. There are a few unedited variables that are computed by the processing system, such as the ATUS final weight (TUFNWGTP).
E	Edited Variable	An edited variable is one that has gone through an editing process (a process checking for consistency). Values of edited variables are almost always equal to values of the corresponding unedited variables. Data differ when a value is allocated or imputed by the processing system based on allocation rules specified in CPS or ATUS processing. Allocations are typically performed when the unedited variable contains a value of blank, "don't know," or "refused."<br><br>An edited version of a variable exists only if that variable goes through an editing process. If there are no edits for a variable, then only an unedited version of that variable exists.
R	Recode	A recode is a variable calculated by the processing system from a combination of other variables on the file. For example, TRMJOCC1 is the major occupation code for the respondent’s main job; this is not a response to a question but rather a variable that summarizes (or “groups”) the more finely detailed occupation variable TEIO1OCD. (Note that variables with second and third characters of “RT” are summary variables.)
RT	Summary Variable	These variables summarize the amount of time respondents spent with other people or did selected activities. For example, TRTALONE gives the total amount of nonwork-related time the respondent spent alone on the diary day. Variables that summarize the amount of time respondents spent with other people rely on “who” code information and therefore do not include activities for which no “who” code information was collected, such as sleeping.
T	Topcode Flag	These variables indicate whether another variable has been topcoded, or given a maximum value. The three topcode variables on the ATUS interview data files all relate to earnings.

Using these rules, variables can be more readily understood based on their names. For example, the variable TEAGE can be broken down as follows:

- The first character “T” indicates that this variable was collected or created through the ATUS interviews
- The second character “E” indicates that this variable went through an editing process
- The final part of the variable name, “AGE,” is descriptive

Some questions asked in the ATUS interview allow for more than one response. For such multiple entry questions, there is a separate variable for each possible response. Each variable has the same descriptive name but a different (sequential) number. For example, respondents can provide up to six answers to the question “You said you have been trying to find work – how did you go about looking?” The variable names are TULKDK1, TULKDK2, TULKDK3, etc.

Not all ATUS variables are on the files. When there is an edited variable, the corresponding unedited variable is usually omitted from the files. This is typically done to protect the confidentiality of ATUS respondents as required by law. If an unedited variable is included on the files, then an edited version does not exist and the unedited version cannot be used

to identify individual respondents. In addition, some variables available on the single-year data files are not available on the 2003-23 files as a way to keep file sizes manageable. These variables are listed in Appendices B, C, D, E and F.

Allocation Flags

The 2003-23 ATUS Interview files contain two allocation flags: TRHERNAL and TRWERNAL. These allocation flags were included because of the frequency with which earnings variables are allocated in the ATUS. All other allocation flags appear only on the single-year ATUS Interview files and *not* on the multi-year files. For more information on these allocation flags, please see the single-year Interview data dictionaries, available online at www.bls.gov/tus/dictionaries.htm.

Edited Universe

Edited variables and recodes are defined for certain universes, and these are listed in the data dictionary. For example, TEIO1OCD (occupation code) is only defined when the respondent is employed. Therefore, the universe for TEIO1OCD is TELFS = 1 or 2 (TELFS is the labor force status of the respondent, and values of 1 or 2 indicate that the respondent is employed).

Certain variables might initially appear to be the same because their descriptions are very similar. These variables are different in that they were asked of different groups of survey respondents. For example, the variables TEERNH1O and TEERNH2 both have the same question text of “Excluding overtime pay, tips, and commissions, what is your hourly rate of pay on your main job?” The difference in these two variables has to do with which respondents were asked each question. This can be determined by looking at the edited universes. TEERNH1O was asked of respondents with TEERNPER = 1, or those who said it was easiest to report their earnings hourly. TEERNH2, on the other hand, was asked of respondents with TEERNRT = 1, or those who said they were paid hourly but reported their earnings another way

Organization of the Data Dictionary

Variables are listed in the data dictionary in alphabetical order.

Below is a sample entry from the ATUS interview data dictionary:

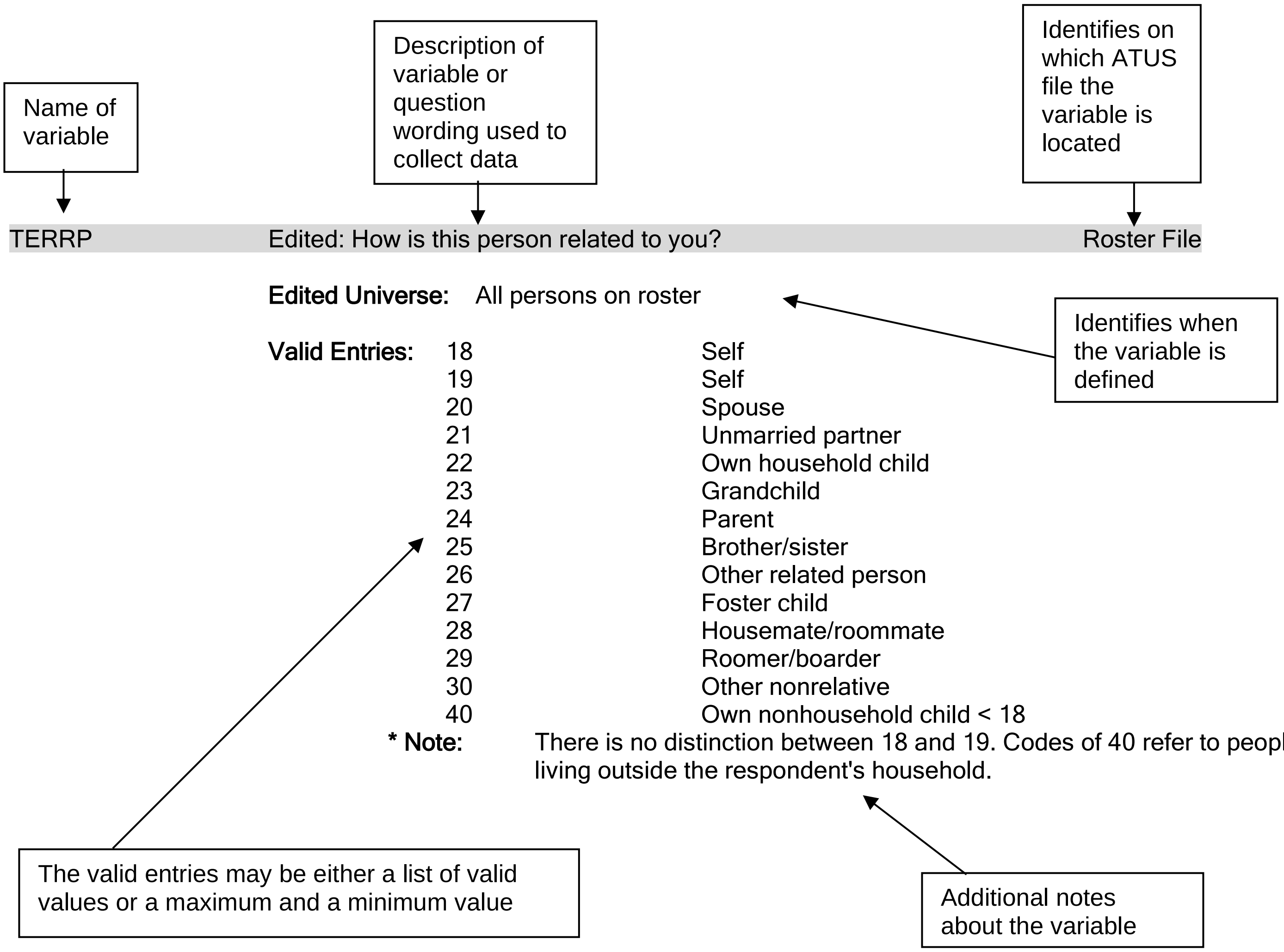

Frequently Used Variables

The ATUS files have many variables and users may sometimes have difficulty determining which variables to use. A list of the most commonly used ATUS variables is available at https://www.bls.gov/tus/other-documentation/freqvariables.pdf.

Changes between years of ATUS data

Those using the multi-year files should be aware of changes to ATUS survey methods between years—such as new, discontinued, and changed variables—as well as differences in activity codes between years. For a list of these changes, see the document describing ATUS changes (https://www.bls.gov/tus/other-documentation/changes.pdf). Note that the multi-year files have their own Activity Coding Lexicon (https://www.bls.gov/tus/lexicons/lexiconnoex0323.pdf) that differs somewhat from the single-year lexicons. The multi-year coding lexicon accounts for the differences in activity codes between years.

Combining multiple years of ATUS Data

The method used to generate statistical weights (the variable TUFINLWGT) on the ATUS files changed each year from 2003 to 2006. There were no changes to the method used to generate TUFINLWGT in 2007-2019 and 2021 and later years. For ease of use, this multi-year data set provides statistical weights developed using the 2006 weighting method. The weighting variable has been named TUFNWGTP and it is available for the years 2003-2019 and 2021 and beyond. All other weighting variables (e.g. TU04FWGT and TU06FWGT) were removed from the multi-year files.

The method used to develop statistical weights for the 2020 ATUS data was modified. Time-use estimates produced with the 2020 statistical weights represent only the days before and after the pandemic-related shutdown in data collection. That is, they will represent the periods January 1st to March 17th and May 10th to December 31st. Because data were not collected about March 18th to May 9th -- a period during which people were encouraged to stay home and physically distance themselves from those with whom they did not live, and a period in which one would expect people's use of time to be greatly impacted – the data that were collected in 2020 are not representative of the full year. While it's possible to combine the 2020 data with other years, researchers should be careful using multiple years of ATUS data to produce estimates. Annual estimates with the 2020 data are not possible, but researchers can produce partial-year estimates using data collected before or after the pandemic-related shutdown. When using the 2020 ATUS data, researchers need to use the weighting variable TU20FWGT instead of TUFNWGTP.

For more information about ATUS statistical weights, why researchers should use them, and details about how the ATUS weighting method changed, see the ATUS User's Guide (www.bls.gov/tus/atususersguide.pdf).

2003-2023 ATUS Data Dictionary: Public ATUS Interview Data

**Name**	**Description**			**File**
TEABSRSN	Edited: what was the main reason you were absent from your job last week?			Respondent File
	**Edited Universe:**	TELFS = 2		
	**Valid Entries:**	1	On layoff (temporary or indefinite)	
		2	Slack work/business conditions	
		3	Waiting for a new job to begin	
		4	Vacation/personal days	
		5	Own illness/injury/medical problems	
		6	Childcare problems	
		7	Other family/personal obligation	
		8	Maternity/paternity leave	
		9	Labor dispute	
		10	Weather affected job	
		11	School/training	
		12	Civic/military duty	
		13	Does not work in the business	
		14	Other	
**Name**	**Description**			**File**
TEAGE	Edited: age			Roster File,<br>Activity Summary File
	**Edited Universe:**	All persons on roster		
	**Valid Entries:**	0<br>85	Min Value<br>Max Value	
	***Note**	For TUYEAR < = 2004, TEAGE is topcoded to 80. All those age 80 and above have TEAGE=80.<br><br>For TUYEAR > = 2005, TEAGE is topcoded to 85. All those age 80 through 84 have TEAGE = 80. Those age 85 or above have TEAGE = 85.		
**Name**	**Description**			**File**
TEAGE_EC	Edited: age of eldercare recipient			EC Roster File
	**Edited Universe:**	All eldercare recipients		
	**Valid Entries:**	0<br>85	Min Value<br>Max Value	
	***Note**	Questions about eldercare were introduced in January 2011. For household members, this is the age on the diary day; for nonhousehold members it's the person's age on the first of the month for the month corresponding to 3 months before the interview.<br><br>TEAGE_EC is topcoded to 85. All those age 80 through 84 have TEAGE_EC = 80. Those age 85 or above have TEAGE_EC = 85. TXAGE_EC indicates topcoding.		

2003-2023 ATUS Data Dictionary: Public ATUS Interview Data

Name	Description			File
TEELDUR	Edited: how long have you provided care to [NAME]?			EC Roster File
	**Edited Universe:**	All eldercare recipients		
	**Valid Entries:**	1	0 to 5 months	
		2	6 to 11 months	
		3	1 year	
		4	More than a year	
	***Note**	Questions about eldercare were introduced in January 2011. The name is filled with the information collected from the TUELWHO question.		

2003-2023 ATUS Data Dictionary: Public ATUS Interview Data

Name	Description			File
TEELWHO	Edited: who did you give this care to?			EC Roster File
	**Edited Universe:**	All eldercare recipients		
	**Valid Entries:**	20	Spouse	
		21	Unmarried partner	
		22	Own household child	
		23	Grandchild	
		24	Parent	
		25	Brother/sister	
		26	Other related person	
		27	Foster child	
		28	Housemate/roommate	
		29	Roomer/boarder	
		30	Other nonrelative	
		33	Mother	
		34	Father	
		35	Spouse	
		36	Partner	
		37	Brother	
		38	Sister	
		39	Mother-in-law	
		40	Father-in-law	
		41	Aunt	
		42	Uncle	
		43	Friend	
		44	Neighbor	
		46	Grandparent (see note)	
		47	Grandmother/Great-grandmother (see note)	
		48	Grandfather/Great-grandfather (see note)	
		49	Other related person (see note)	
		55	Other (see note)	
		56	Other non-relative (see note)	
	***Note**	Questions about eldercare were introduced in 2011. All codes of 30 or less refer to people living inside of the respondent's household.<br><br>Beginning in January 2013, values 47, 48, 49, and 56 were added; values 46 (grandparent/great-grandparent) and 55 (other) are no longer valid.		

2003-2023 ATUS Data Dictionary: Public ATUS Interview Data

Name	Description			File
TEELYRS	Edited: how many years have you provided care (to this person)?			EC Roster File
	**Edited Universe:**	TEELDUR=4		
	**Valid Entries:**	1<br>99	Min Value<br>Max Value	
	***Note**	Questions about eldercare were introduced in January 2011.		
**Name**	**Description**			**File**
TEERN	Edited: total weekly overtime earnings (2 implied decimals)			Respondent File
	**Edited Universe:**	TEERNUOT = 1 and TEERNPER = 1		
	**Valid Entries:**	0<br>288461	Min Value<br>Max Value	
**Name**	**Description**			**File**
TEERNH1O	Edited: excluding overtime pay, tips, and commissions, what is your hourly rate of pay on your main job? (2 implied decimals)			Respondent File
	**Edited Universe:**	TEERNPER = 1		
	**Valid Entries:**	0<br>9999	Min Value<br>Max Value	
**Name**	**Description**			**File**
TEERNH2	Edited: excluding overtime pay, tips, and commissions, what is your hourly rate of pay on your main job? (2 implied decimals)			Respondent File
	**Edited Universe:**	TEERNRT = 1		
	**Valid Entries:**	0<br>9999	Min Value<br>Max Value	
**Name**	**Description**			**File**
TEERNHRO	Edited: how many hours do you usually work per week at this rate?			Respondent File
	**Edited Universe:**	TEERNH1O >= 0		
	**Valid Entries:**	1<br>99	Min Value<br>Max Value	
**Name**	**Description**			**File**
TEERNHRY	Edited: hourly/non-hourly status			Respondent File
	**Edited Universe:**	TELFS = 1 or 2 and TEIO1COW = 1 - 5		
	**Valid Entries:**	1	Paid hourly	
		2	Not paid hourly	

2003-2023 ATUS Data Dictionary: Public ATUS Interview Data

Name	Description			File
TEERNPER	Edited: for your main job, what is the easiest way for you to report your total earnings before taxes or other deductions: hourly, weekly, annually, or some other way?			Respondent File
	**Edited Universe:**	TELFS = 1 or 2 and TEIO1COW = 1 - 5		
	**Valid Entries:**	1	Hourly	
		2	Weekly	
		3	Bi-weekly	
		4	Twice monthly	
		5	Monthly	
		6	Annually	
		7	Other	
**Name**	**Description**			**File**
TEERNRT	Edited: even though you told me it is easier to report your earnings another way, are you paid at an hourly rate on this job?			Respondent File
	**Edited Universe:**	TEERNPER = 2 - 7		
	**Valid Entries:**	1	Yes	
		2	No	
**Name**	**Description**			**File**
TEERNUOT	Edited: do you usually receive overtime pay, tips, or commissions at your main job?			Respondent File
	**Edited Universe:**	TELFS = 1 or 2 and TEIO1COW = 1 - 5		
	**Valid Entries:**	1	Yes	
		2	No	
**Name**	**Description**			**File**
TEERNWKP	Edited: how many weeks a year do you get paid?			Respondent File
	**Edited Universe:**	TEERNPER = 6		
	**Valid Entries:**	1<br>52	Min Value<br>Max Value	
**Name**	**Description**			**File**
TEHRFTPT	Edited: do you usually work more than 35 hours per week at your job(s)/family business?			Respondent File
	**Edited Universe:**	TEHRUSL1 = -4 or TEHRUSL2 = -4		
	**Valid Entries:**	1	Yes	
		2	No	
		3	Hours vary	

2003-2023 ATUS Data Dictionary: Public ATUS Interview Data

Name	Description		File
TEHRUSL1	Edited: how many hours per week do you usually work at your main job?		Respondent File
	**Edited Universe:**	TELFS = 1 or 2	
	**Valid Entries:**	0<br>99	Min Value<br>Max Value
	***Note**	-4 (Hours vary) is also valid for TEHRUSL1	

Name	Description		File
TEHRUSL2	Edited: how many hours per week do you usually work at your other job(s)?		Respondent File
	**Edited Universe:**	TELFS = 1 or 2 and TEMJOT = 1	
	**Valid Entries:**	0<br>99	Min Value<br>Max Value
	***Note**	-4 (Hours vary) is also valid for TEHRUSL2	

Name	Description		File
TEHRUSLT	Edited: total hours usually worked per week (sum of TEHRUSL1 and TEHRUSL2)		Respondent File,<br>Activity Summary File
	**Edited Universe:**	TELFS = 1 or 2	
	**Valid Entries:**	0<br>999	Min Value<br>Max Value
	***Note**	-4 (Hours vary) is also valid for TEHRUSLT	

Name	Description		File
TEIO1COW	Edited: individual class of worker code (main job)		Respondent File
	**Edited Universe:**	TELFS = 1 or 2	
	**Valid Entries:**	1	Government, federal
		2	Government, state
		3	Government, local
		4	Private, for profit
		5	Private, nonprofit
		6	Self-employed, incorporated
		7	Self-employed, unincorporated
		8	Without pay

2003-2023 ATUS Data Dictionary: Public ATUS Interview Data

Name	Description			File
TEIO1ICD	Edited: industry code (main job)			Respondent File
	**Edited Universe:**	TELFS = 1 or 2		
	**Valid Entries:**	0<br>9999	Min Value<br>Max Value	
	***Note**	Beginning with the 2020 ATUS, industry data were classified using the 2017 Census Industry Classification System. This system replaced the 2012 Census Industry Classification System.<br><br>The 2012 Census Industry Classification System was incorporated beginning in the 2014 ATUS; it replaced the 2007 Census Industry Classification System, which was used by ATUS from 2010 until the 2012 classification scheme was implemented.<br><br>The 2007 Census Industry Classification System was incorporated beginning in the 2010 ATUS; it replaced the 2002 Census Industry Classification System, which was used by ATUS from 2003 until the 2007 classification scheme was implemented.<br><br>Refer to Appendix A for a list of industry codes using the 2002, 2007, 2012, and 2017 Census Industry Classification systems.		
**Name**	**Description**			**File**
TEIO1OCD	Edited: occupation code (main job)			Respondent File
	**Edited Universe:**	TELFS = 1 or 2		
	**Valid Entries:**	0<br>9999	Min Value<br>Max Value	
	***Note**	Beginning with the 2020 ATUS, occupation data were classified using the 2018 Census Occupation Classification System. This system replaced the 2010 Census Occupation Classification System.<br><br>The 2010 Census Occupation Classification System was incorporated beginning in the 2011 ATUS; it replaced the 2002 Census Occupation Classification System, which was used by ATUS from 2003 until the 2010 classification scheme was implemented. The ATUS occupation data are not strictly comparable across all the years.<br><br>Refer to Appendix A for a list of occupation codes using the 2002, 2010, and 2018 Census Occupation Classification systems.		
**Name**	**Description**			**File**
TELAYAVL	Edited: could you have returned to work in the last seven days if you had been recalled?			Respondent File
	**Edited Universe:**	TELFS = 3		
	**Valid Entries:**	1	Yes	
		2	No	
**Name**	**Description**			**File**
TELAYLK	Edited: even though you expect to be called back to work, have you been looking for work during the last four weeks?			Respondent File
	**Edited Universe:**	TELAYAVL = 1 or 2		
	**Valid Entries:**	1	Yes	
		2	No	

2003-2023 ATUS Data Dictionary: Public ATUS Interview Data

Name	Description			File
TELFS	Edited: labor force status			Respondent File, Activity Summary File
	**Edited Universe:**	All respondents		
	**Valid Entries:**	1	Employed - at work	
		2	Employed - absent	
		3	Unemployed - on layoff	
		4	Unemployed - looking	
		5	Not in labor force	

Name	Description			File
TELKAVL	Edited: could you have started a job in the last seven days if one had been offered?			Respondent File
	**Edited Universe:**	TELKM1 = 1 - 13		
	**Valid Entries:**	1	Yes	
		2	No	

Name	Description			File
TELKM1	Edited: what are all of the things you have done to find work during the last 4 weeks? (first method)			Respondent File
	**Edited Universe:**	TELFS = 4		
	**Valid Entries:**	1	Contacted employer directly/interview	
		2	Contacted public employment agency	
		3	Contacted private employment agency	
		4	Contacted friends or relatives	
		5	Contacted school/university employment center	
		6	Sent out resumes/filled out applications	
		7	Checked union/professional registers	
		8	Placed or answered ads	
		9	Other active	
		10	Looked at ads	
		11	Attended job training programs/courses	
		12	Nothing	
		13	Other passive	
	***Note**	In order to research job search methods, users must combine all fields TELKM1, TULKM2 - TULKM6, TULKDK1 - TULKDK6, and TULKPS1 - TULKPS6		

2003-2023 ATUS Data Dictionary: Public ATUS Interview Data

Name	Description			File
TEMJOT	Edited: in the last seven days did you have more than one job?			Respondent File,<br>Activity Summary File
	**Edited Universe:**	TELFS = 1 or 2		
	**Valid Entries:**	1	Yes	
		2	No	
**Name**	**Description**			**File**
TERET1	Edited: do you currently want a job, either full or part time?			Respondent File
	**Edited Universe:**	TELFS = 5 and (TURETOT = 1 or TUFABS = 3 or TUFWK = 3 or TULAY = 3) and TEAGE >= 50		
	**Valid Entries:**	1	Yes or maybe/it depends	
		2	No	
		3	Has a job	
**Name**	**Description**			**File**
TERRP	Edited: how is this person related to you?			Roster File
	**Edited Universe:**	All persons on roster		
	**Valid Entries:**	18	Self	
		19	Self	
		20	Spouse	
		21	Unmarried partner	
		22	Own household child	
		23	Grandchild	
		24	Parent	
		25	Brother/sister	
		26	Other relative	
		27	Foster child	
		28	Housemate/roommate	
		29	Roomer/boarder	
		30	Other nonrelative	
		40	Own nonhousehold child < 18	
	***Note**	There is no distinction between 18 and 19. Codes of 40 refer to people living outside the respondent's household.		

2003-2023 ATUS Data Dictionary: Public ATUS Interview Data

Name	Description			File
TESCHENR	Edited: are you enrolled in high school, college, or university?			Respondent File, Activity Summary File
	**Edited Universe:**	Respondents aged 15 to 49		
	**Valid Entries:**	1	Yes	
		2	No	
**Name**	**Description**			**File**
TESCHFT	Edited: are you enrolled as a full-time or part-time student?			Respondent File
	**Edited Universe:**	TESCHENR = 1		
	**Valid Entries:**	1	Full time	
		2	Part time	
**Name**	**Description**			**File**
TESCHLVL	Edited: would that be high school, college, or university?			Respondent File, Activity Summary File
	**Edited Universe:**	TESCHENR = 1		
	**Valid Entries:**	1	High school	
		2	College or university	
**Name**	**Description**			**File**
TESEX	Edited: sex			Roster File, Activity Summary File
	**Edited Universe:**	All persons on roster		
	**Valid Entries:**	1	Male	
		2	Female	
**Name**	**Description**			**File**
TESPEMPNOT	Edited: employment status of spouse or unmarried partner			Respondent File, Activity Summary File
	**Edited Universe:**	TRSPPRES = 1 or 2		
	**Valid Entries:**	1	Employed	
		2	Not employed	
**Name**	**Description**			**File**
TESPUHRS	Edited: usual hours of work per week of spouse or unmarried partner			Respondent File
	**Edited Universe:**	TESPEMPNOT = 1		
	**Valid Entries:**	0<br>99	Min Value<br>Max Value	
	***Note**	-4 (Hours vary) is also valid for TESPUHRS		

2003-2023 ATUS Data Dictionary: Public ATUS Interview Data

Name	Description			File
TEWHERE	Edited: where were you during the activity?			Activity File
	**Edited Universe:**	All activities (except those noted below)		
	**Valid Entries:**	1	Respondent's home or yard	
		2	Respondent's workplace	
		3	Someone else's home	
		4	Restaurant or bar	
		5	Place of worship	
		6	Grocery store	
		7	Other store/mall	
		8	School	
		9	Outdoors away from home	
		10	Library	
		11	Other place	
		12	Car, truck, or motorcycle (driver)	
		13	Car, truck, or motorcycle (passenger)	
		14	Walking	
		15	Bus	
		16	Subway/train	
		17	Bicycle	
		18	Boat/ferry	
		19	Taxi/limousine service	
		20	Airplane	
		21	Other mode of transportation	
		30	Bank	
		31	Gym/health club	
		32	Post Office	
		89	Unspecified place	
		99	Unspecified mode of transportation	
	***Note**	Values of 30, 31 and 32 for TEWHERE were introduced in October 2004 ATUS. Therefore, for (TUMONTH < 10 AND TUYEAR = 2004) these values will not appear.<br><br>TEWHERE is not collected for activities with activity codes of 0101xx, 0102xx, 0104xx, 500105, or 500106.		

2003-2023 ATUS Data Dictionary: Public ATUS Interview Data

Name	Description		File
TR_03CC57	Was a household or own non-household child < 13 in your care during this activity?		Activity File
	**Edited Universe:**	All activities	
	**Valid Entries:**	1	At least one child < 13 was in respondent's care during activity
	***Note**	This variable was discontinued in 2004 ATUS. Therefore, for [TUYEAR > 2003], there will be missing values of TR_03CC57. It was replaced by the component variables TUCC5, TUCC5B, and TUCC7.	

Name	Description		File
TRCHILDNUM	Number of household children < 18		Respondent File,<br>Activity Summary File
	**Edited Universe:**	All respondents	
	**Valid Entries:**	0<br>30	Min Value<br>Max Value

Name	Description		File
TRCODEP	Pooled six-digit activity code		Activity File
	***Note**	See the 2003-23 Activity Coding Lexicon for activity definitions	

Name	Description		File
TRDPFTPT	Full time or part time employment status of respondent		Respondent File,<br>Activity Summary File
	**Edited Universe:**	TELFS = 1 or 2	
	**Valid Entries:**	1	Full time
		2	Part time

Name	Description		File
TRDTIND1	Detailed industry recode (main job)		Respondent File
	**Edited Universe:**	TELFS = 1 or 2	
	**Valid Entries:**	1<br>51	Min Value<br>Max Value
	***Note**	Beginning with the 2020 ATUS, industry data were classified using the 2017 Census Industry Classification System. This system replaced the 2012 Census Industry Classification System.<br><br>The 2012 Census Industry Classification System was incorporated beginning in the 2014 ATUS; it replaced the 2007 Census Industry Classification System, which was used by ATUS from 2010 until the 2012 classification scheme was implemented.<br><br>The 2007 Census Industry Classification System was incorporated beginning in the 2010 ATUS; it replaced the 2002 Census Industry Classification System, which was used by ATUS from 2003 until the 2007 classification scheme was implemented.<br><br>Refer to Appendix A for a list of industry codes using the 2002, 2007, 2012, and 2017 Census Industry Classification systems.	

2003-2023 ATUS Data Dictionary: Public ATUS Interview Data

Name	Description			File
TRDTOCC1	Detailed occupation recode (main job)			Respondent File
	**Edited Universe:**	TELFS = 1 or 2		
	**Valid Entries:**	1	Management occupations	
		2	Business and financial operations occupations	
		3	Computer and mathematical science occupations	
		4	Architecture and engineering occupations	
		5	Life, physical, and social science occupations	
		6	Community and social service occupations	
		7	Legal occupations	
		8	Education, training, and library occupations	
		9	Arts, design, entertainment, sports, and media occupations	
		10	Healthcare practitioner and technical occupations	
		11	Healthcare support occupations	
		12	Protective service occupations	
		13	Food preparation and serving related occupations	
		14	Building and grounds cleaning and maintenance occupations	
		15	Personal care and service occupations	
		16	Sales and related occupations	
		17	Office and administrative support occupations	
		18	Farming, fishing, and forestry occupations	
		19	Construction and extraction occupations	
		20	Installation, maintenance, and repair occupations	
		21	Production occupations	
		22	Transportation and material moving occupations	
	***Note**	Beginning with the 2020 ATUS, occupation data were classified using the 2018 Census Occupation Classification System. This system replaced the 2010 Census Occupation Classification System.<br><br>The 2010 Census Occupation Classification System was incorporated beginning in the 2011 ATUS; it replaced the 2002 Census Occupation Classification System, which was used by ATUS from 2003 until the 2010 classification scheme was implemented. The ATUS occupation data are not strictly comparable across all the years.<br><br>Refer to Appendix A for a list of occupation codes using the 2002, 2010, and 2018 Census Occupation Classification systems.		

2003-2023 ATUS Data Dictionary: Public ATUS Interview Data

Name	Description		File
TRELHH	Eldercare recipient is a household member		EC Roster File
	**Edited Universe:**	All Eldercare recipients	
	**Valid Entries:**	0	Recipient is not a household member
		1	Recipient is a household member
	***Note**	Questions about eldercare were introduced in January 2011.	
**Name**	**Description**		**File**
TREMODR	Eating and Health Module respondent		Respondent File
	**Edited Universe:**	All respondents	
	**Valid Entries:**	0	Did not respond to Eating and Health Module
		1	Responded to Eating and Health Module
	***Note**	The Eating and Health Module interview was conducted for TUYEAR = 2006, 2007, 2008, 2014, 2015, 2016, 2022, and 2023. For these years all respondents were selected to be interviewed for the Eating and Health Module. Values of 0 indicate that the individual did not complete an Eating and Health Module interview.	
**Name**	**Description**		**File**
TRERNHLY	Hourly earnings at main job (2 implied decimals)		Respondent File
	**Edited Universe:**	TEERNHRY = 1	
	**Valid Entries:**	0<br>9999	Min Value<br>Max Value
	***Note**	This is the most-frequently used hourly earnings variable in ATUS and is only defined for employed persons who say they work hourly and are not self-employed or without pay. The allocation flag for this variable is TRHERNAL. Subject to topcoding based on the entry in TEERNHRO such that TEERNHRO x TRERNHLY <= 2884.61; topcoding is indicated in TTHR.	
**Name**	**Description**		**File**
TRERNUPD	Earnings update flag		Respondent File
	**Edited Universe:**	TELFS = 1 or 2 and TEIO1COW = 1 - 5	
	**Valid Entries:**	0	Earnings carried forward from final CPS interview
		1	Earnings updated in ATUS
**Name**	**Description**		**File**
TRERNWA	Weekly earnings at main job (2 implied decimals)		Respondent File,<br>Activity Summary File
	**Edited Universe:**	TELFS = 1 or 2 and TEIO1COW = 1 - 5	
	**Valid Entries:**	0<br>288461	Min Value<br>Max Value
	***Note**	This is the most-frequently used earnings variable in ATUS and is defined for all employed persons who are not self-employed or without pay. The allocation flag for this variable is TRWERNAL. Subject to topcoding (the maximum value cannot be greater than 2884.61); topcoding is indicated in TTOT, TTWK, and TTHR.	

2003-2023 ATUS Data Dictionary: Public ATUS Interview Data

Name	Description			File
TRHERNAL	TRERNHLY: allocation flag			Respondent File
	**Edited Universe:**	TEERNHRY = 1		
	**Valid Entries:**	0	TRERNHLY does not contain allocated information	
		1	TRERNHLY contains allocated information	
**Name**	**Description**			**File**
TRHHCHILD	Presence of household children < 18			Respondent File
	**Edited Universe:**	All respondents		
	**Valid Entries:**	1	Yes	
		2	No	
**Name**	**Description**			**File**
TRHOLIDAY	Flag to indicate if diary day was a holiday			Respondent File,<br>Activity Summary File
	**Edited Universe:**	All respondents		
	**Valid Entries:**	0	Diary day was not a holiday	
		1	Diary day was a holiday	
	***Note**	New Year's Day, Easter, Memorial Day, the Fourth of July, Labor Day, Thanksgiving Day, and Christmas Day are identified as holidays.<br>If the interviewers did not work on the day following the holiday, data about that holiday were not collected.		

2003-2023 ATUS Data Dictionary: Public ATUS Interview Data

Name	Description		File
TRIMIND1	Intermediate industry recode (main job)		Respondent File
	**Edited Universe:**	TELFS = 1 or 2	
	**Valid Entries:**	1	Agriculture, forestry, fishing, and hunting
		2	Mining
		3	Construction
		4	Manufacturing - durable goods
		5	Manufacturing - non-durable goods
		6	Wholesale trade
		7	Retail trade
		8	Transportation and warehousing
		9	Utilities
		10	Information
		11	Finance and insurance
		12	Real estate and rental and leasing
		13	Professional and technical services
		14	Management, administrative and waste management services
		15	Educational services
		16	Health care and social services
		17	Arts, entertainment, and recreation
		18	Accommodation and food services
		19	Private households
		20	Other services, except private households
		21	Public administration

Name	Description		File
TRLVMODR	Leave module respondent		Respondent File
	**Edited Universe:**	TEIO1COW = 1 - 6 (2011)<br>TEIO1COW = 1 - 5 (2017-2018)	
	**Valid Entries:**	0	Respondent was eligible for the Leave module, but did not complete it.
		1	Respondent completed Leave module.
	***Note**	The Leave Module was conducted in 2011, 2017, and 2018. For TUYEAR = 2011, wage and salary workers and self-employed incorporated workers were selected to be interviewed for the Leave Module. For TUYEAR = 2017 and 2018 only wage and salary workers were selected to be interviewed. For TUYEAR = 2011, 2017, and 2018, values of -1 indicate the respondent was not eligible for the module.	

2003-2023 ATUS Data Dictionary: Public ATUS Interview Data

Name	Description			File
TRMJIND1	Major industry recode (main job)			Respondent File
	**Edited Universe:**	TELFS = 1 or 2		
	**Valid Entries:**	1	Agriculture, forestry, fishing, and hunting	
		2	Mining	
		3	Construction	
		4	Manufacturing	
		5	Wholesale and retail trade	
		6	Transportation and utilities	
		7	Information	
		8	Financial activities	
		9	Professional and business services	
		10	Educational and health services	
		11	Leisure and hospitality	
		12	Other services	
		13	Public administration	

Name	Description			File
TRMJOCC1	Major occupation recode (main job)			Respondent File
	**Edited Universe:**	TELFS = 1 or 2		
	**Valid Entries:**	1	Management, business, and financial occupations	
		2	Professional and related occupations	
		3	Service occupations	
		4	Sales and related occupations	
		5	Office and administrative support occupations	
		6	Farming, fishing, and forestry occupations	
		7	Construction and extraction occupations	
		8	Installation, maintenance, and repair occupations	
		9	Production occupations	
		10	Transportation and material moving occupations	
	***Note**	Beginning with the 2020 ATUS, occupation data were classified using the 2018 Census Occupation Classification System. This system replaced the 2010 Census Occupation Classification System.<br><br>The 2010 Census Occupation Classification System was incorporated beginning in the 2011 ATUS; it replaced the 2002 Census Occupation Classification System, which was used by ATUS from 2003 until the 2010 classification scheme was implemented. The ATUS occupation data are not strictly comparable across all the years.		

2003-2023 ATUS Data Dictionary: Public ATUS Interview Data

Name	Description			File
TRMJOCGR	Major occupation category (main job)			Respondent File
	**Edited Universe:**	TELFS = 1 or 2		
	**Valid Entries:**	1	Management, professional, and related occupations	
		2	Service occupations	
		3	Sales and office occupations	
		4	Farming, fishing, and forestry occupations	
		5	Construction and maintenance occupations	
		6	Production, transportation, and material moving occupations	
	***Note**	Beginning with the 2020 ATUS, occupation data were classified using the 2018 Census Occupation Classification System. This system replaced the 2010 Census Occupation Classification System.<br><br>The 2010 Census Occupation Classification System was incorporated beginning in the 2011 ATUS; it replaced the 2002 Census Occupation Classification System, which was used by ATUS from 2003 until the 2010 classification scheme was implemented. The ATUS occupation data are not strictly comparable across all the years.		
**Name**	**Description**			**File**
TRNHHCHILD	Presence of own non-household child < 18			Respondent File
	**Edited Universe:**	All respondents		
	**Valid Entries:**	1	Yes	
		2	No	
**Name**	**Description**			**File**
TRNUMHOU	Number of people living in respondent's household			Respondent File
	**Edited Universe:**	All respondents		
	**Valid Entries:**	1<br>30	Min Value<br>Max Value	
**Name**	**Description**			**File**
TROHHCHILD	Presence of own household children < 18			Respondent File
	**Edited Universe:**	All respondents		
	**Valid Entries:**	1	Yes	
		2	No	

2003-2023 ATUS Data Dictionary: Public ATUS Interview Data

Name	Description		File
TRSPFTPT	Full time or part time employment status of spouse or unmarried partner		Respondent File, Activity Summary File
	**Edited Universe:**	TESPEMPNOT = 1	
	**Valid Entries:**	1	Full time
		2	Part time
		3	Hours vary

Name	Description		File
TRSPPRES	Presence of the respondent's spouse or unmarried partner in the household		Respondent File, Activity Summary File
	**Edited Universe:**	All respondents	
	**Valid Entries:**	1	Spouse present
		2	Unmarried partner present
		3	No spouse or unmarried partner present

Name	Description		File
TRTALONE	Total nonwork-related time respondent spent alone (in minutes)		Respondent File
	**Edited Universe:**	All respondents	
	**Valid Entries:**	0<br>1440	Min Value<br>Max Value
	***Note**	This variable is computed using TUWHO_CODE information; time spent working and all activities for which who information is not collected, such as sleeping, are omitted from the calculation	

Name	Description		File
TRTALONE_WK	Total work- and nonwork-related time respondent spent alone (in minutes)		Respondent File
	**Edited Universe:**	All respondents	
	**Valid Entries:**	0<br>1440	Min Value<br>Max Value
	***Note**	This variable is computed using TUWHO_CODE information; all activities for which who information is not collected, such as sleeping, are excluded from the calculation<br><br>Starting in January 2010, TUWHO_CODE information was collected for work activities (0501xx)<br><br>TRTALONE_WK was introduced in 2010. Therefore, cases with [TUYEAR < 2010] will have missing values.	

2003-2023 ATUS Data Dictionary: Public ATUS Interview Data

**Name**	**Description**		**File**
TRTCC	Total time spent during diary day providing secondary childcare for household and own nonhousehold children < 13 (in minutes)		Respondent File
	**Edited Universe:**	All respondents	
	**Valid Entries:**	0<br>1440	Min Value<br>Max Value
	***Note**	TRTCC is the sum of all values of TRTCC_LN for each TUCASEID	

**Name**	**Description**		**File**
TRTCC_LN	Total time spent during activity providing secondary child care for household and own nonhousehold children < 13 (in minutes)		Activity File
	**Edited Universe:**	All activities for respondents who have at least one household or own nonhousehold child < 13	
	**Valid Entries:**	0<br>1440	Min Value<br>Max Value
	***Note**	For TUYEAR > = 2004, TRTCC_LN is the maximum for the activity of the following variables: TRTOHH_LN, TRTNOHH_LN, and TRTONHH_LN.<br><br>TRTCC_LN is not calculated for activities when no household child < 13 was awake. It is also not calculated for households that do not have at least one household or nonhousehold child < 13.	

**Name**	**Description**		**File**
TRTCCC	Total nonwork-related time respondent spent with customers, clients, and co-workers (in minutes)		Respondent File
	**Edited Universe:**	All respondents	
	**Valid Entries:**	0<br>1440	Min Value<br>Max Value
	***Note**	This variable is computed using TUWHO_CODE information; time spent working and all activities for which who information is not collected, such as sleeping, are omitted from the calculation.<br><br>For cases with [TUYEAR > 2009], TUWHO_CODE = (59, 60, 61, or 62) is included in this calculation (others may be present)<br><br>For cases with [TUYEAR < 2010], TUWHO_CODE = 55 is included in this calculation (others may be present)	

2003-2023 ATUS Data Dictionary: Public ATUS Interview Data

Name	Description		File
TRTCCC_WK	Total work- and nonwork-related time respondent spent with customers, clients, and coworkers (in minutes)		Respondent File
	**Edited Universe:**	All respondents	
	**Valid Entries:**	0 Min Value<br>1440 Max Value	
	***Note**	This variable is computed using TUWHO_CODE information; all activities for which who information is not collected are omitted from the calculation. TUWHO_CODE = (59, 60, 61, or 62) is included in this calculation (others may be present)<br><br>Starting in January 2010, TUWHO_CODE information was collected for work activities (0501xx)<br><br>TRTCCC_WK was introduced in 2010. Therefore, cases with [TUYEAR < 2010] will have missing values.	

Name	Description		File
TRTCCTOT	Total time spent during diary day providing secondary childcare for all children < 13 (in minutes)		Respondent File
	**Edited Universe:**	All respondents	
	**Valid Entries:**	0 Min Value<br>1440 Max Value	
	***Note**	For TUYEAR > = 2004, TRTCCTOT is the sum of all values of TRTCCTOT_LN for each TUCASEID	

Name	Description		File
TRTCCTOT_LN	Total time spent during activity providing secondary childcare for all children < 13 (in minutes)		Activity File
	**Edited Universe:**	All activities	
	**Valid Entries:**	0 Min Value<br>1440 Max Value	
	***Note**	TRTCCTOT_LN is the maximum for the activity of the following variables: TRTOHH_LN, TRTNOHH_LN, TRTONHH_LN, and TRTCOC_LN.	

Name	Description		File
TRTCHILD	Total nonwork-related time respondent spent with household or nonhousehold children < 18 (in minutes)		Respondent File
	**Edited Universe:**	All respondents	
	**Valid Entries:**	0 Min Value<br>1440 Max Value	
	***Note**	This variable is computed using TUWHO_CODE information; time spent working and all activities for which who information is not collected, such as sleeping, are omitted from the calculation	

2003-2023 ATUS Data Dictionary: Public ATUS Interview Data

Name	Description		File
TRTCOC	Total time spent during diary day providing secondary childcare for nonown, nonhousehold children < 13 (in minutes)		Respondent File
	**Edited Universe:**	All respondents	
	**Valid Entries:**	0<br>1440	Min Value<br>Max Value
	***Note**	TRTCOC is the sum of all TRTCOC_LN for each TUCASEID.	

Name	Description		File
TRTCOC_LN	Total time spent during activity providing secondary child care for nonown, nonhousehold children <13 (in minutes)		Activity File
	**Edited Universe:**	All activities	
	**Valid Entries:**	0<br>1440	Min Value<br>Max Value
	***Note**	TRTCOC_LN is calculated using TUCC8. It does not include activities with TRTIER2P=0101, 0301, 0302, 0303, 0401, 0402, 0403 or activities with TRCODEP= 180381, 180481.	

Name	Description		File
TRTEC	Total time spent providing eldercare (in minutes)		Respondent File
	**Edited Universe:**	TUECYTD=1	
	**Valid Entries:**	0<br>1440	Min Value<br>Max Value
	***Note**	TRTEC is the sum of all values of TRTEC_LN. Excludes time spent in activities with codes = 01xxxx or 0805xx.<br><br>Questions about eldercare and TRTEC were introduced in January 2011. Therefore, cases with [TUYEAR < 2011] will have missing values.	

Name	Description		File
TRTEC_LN	Time (in minutes) spent providing eldercare by activity		Activity File
	**Edited Universe:**	TUEC24 = 1 or 96	
	**Valid Entries:**	0<br>1440	Min Value<br>Max Value
	***Note**	Excludes time spent in activities with codes = 01xxxx or 0805xx.<br><br>Questions about eldercare and TRTEC_LN were introduced in January 2011. Therefore, cases with [TUYEAR < 2011] will have missing values.	

2003-2023 ATUS Data Dictionary: Public ATUS Interview Data

Name	Description		File
TRTFAMILY	Total nonwork-related time respondent spent with family members (in minutes)		Respondent File
	**Edited Universe:**	All respondents	
	**Valid Entries:**	0<br>1440	Min Value<br>Max Value
	***Note**	This variable is computed using TUWHO_CODE information; time spent working and all activities for which who information is not collected, such as sleeping, are omitted from the calculation	
**Name**	**Description**		**File**
TRTFRIEND	Total nonwork-related time respondent spent with friends (in minutes)		Respondent File
	**Edited Universe:**	All respondents	
	**Valid Entries:**	0<br>1440	Min Value<br>Max Value
	***Note**	This variable is computed using TUWHO_CODE information; time spent working and all activities for which who information is not collected, such as sleeping, are omitted from the calculation	
**Name**	**Description**		**File**
TRTHH	Total time spent during diary day providing secondary childcare for household children < 13 (in minutes)		Respondent File
	**Edited Universe:**	All respondents	
	**Valid Entries:**	0<br>1440	Min Value<br>Max Value
	***Note**	TRTHH is the sum of all values of TRTHH_LN for each TUCASEID.	
**Name**	**Description**		**File**
TRTHH_LN	Total time spent during activity providing secondary childcare for household children < 13 (in minutes)		Activity File
	**Edited Universe:**	All activities for respondents with at least one household child < 13	
	**Valid Entries:**	0<br>1440	Min Value<br>Max Value
	***Note**	TRTHH_LN is the maximum for the activity of the following variables: TRTOHH_LN and TRTNOHH_LN.	
**Name**	**Description**		**File**
TRTHHFAMILY	Total nonwork-related time respondent spent with household family members (in minutes)		Respondent File
	**Edited Universe:**	All respondents	
	**Valid Entries:**	0<br>1440	Min Value<br>Max Value
	***Note**	This variable is computed using TUWHO_CODE information; time spent working and all activities for which who information is not collected, such as sleeping, are omitted from the calculation	

2003-2023 ATUS Data Dictionary: Public ATUS Interview Data

Name	Description		File
TRTIER1P	Pooled lexicon tier 1: 1st and 2nd digits of 6-digit activity code		Activity File
	**Valid Entries:**	01 Min Value<br>50 Max Value	
	***Note**	Valid activity codes are listed in the 2003-23 Coding Lexicon. TRCODEP provides the full six-digit activity code for each activity occurring on the Activity file.	
**Name**	**Description**		**File**
TRTIER2P	Pooled lexicon tiers 1 and 2: 1st four digits of 6-digit activity code		Activity File
	***Note**	Valid activity codes are listed in the 2003-23 Coding Lexicon. TRCODEP provides the full six-digit activity code for each activity occurring on the Activity file.	
**Name**	**Description**		**File**
TRTNOCHILD	Total nonwork-related time respondent spent with nonown children < 18 (in minutes)		Respondent File
	**Edited Universe:**	All respondents	
	**Valid Entries:**	0 Min Value<br>1440 Max Value	
	***Note**	This variable is computed using TUWHO_CODE information; time spent working and all activities for which who information is not collected, such as sleeping, are omitted from the calculation	
**Name**	**Description**		**File**
TRTNOHH	Total time spent during diary day providing secondary childcare for nonown household children < 13 (in minutes)		Respondent File
	**Edited Universe:**	All respondents	
	**Valid Entries:**	0 Min Value<br>1440 Max Value	
	***Note**	TRTNOHH is the sum of all values of TRTNOHH_LN for each TUCASEID.	
**Name**	**Description**		**File**
TRTNOHH_LN	Total time spent during activity providing secondary childcare for nonown household children < 13 (in minutes)		Activity File
	**Edited Universe:**	All activities for respondents with at least one nonown household child < 13	
	**Valid Entries:**	0 Min Value<br>1440 Max Value	
	***Note**	TRTNOHH_LN is calculated using TUCC5B. It does not include activities where TRTIER2P= 0101, 0301, 0302, 0303, and where TRCODEP= 180381. It does not include any activity or part of any activity in which no household child was awake (determined by TUCC2 and TUCC4).	
**Name**	**Description**		**File**
TRTO	Total time spent during diary day providing secondary childcare for own children < 13 (in minutes)		Respondent File
	**Edited Universe:**	All respondents	
	**Valid Entries:**	0 Min Value<br>1440 Max Value	
	***Note**	TRTO is the sum of all values of TRTO_LN for each TUCASEID.	

2003-2023 ATUS Data Dictionary: Public ATUS Interview Data

Name	Description			File
TRTO_LN	Total time spent during activity providing secondary childcare for own children < 13 (in minutes)			Activity File
	**Edited Universe:**	All activities for respondents with at least one own child < 13		
	**Valid Entries:**	0<br>1440	Min Value<br>Max Value	
	***Note**	TRTO_LN is the maximum for the activity of the following variables: TRTOHH_LN and TRTONHH_LN.		
**Name**	**Description**			**File**
TRTOHH	Total time spent during diary day providing secondary childcare for own household children < 13 (in minutes)			Respondent File
	**Edited Universe:**	All respondents		
	**Valid Entries:**	0<br>1440	Min Value<br>Max Value	
	***Note**	TRTOHH is the sum of all values of TRTOHH_LN for each TUCASEID.		
**Name**	**Description**			**File**
TRTOHH_LN	Total time spent during activity providing secondary childcare for own household children < 13 (in minutes)			Activity File
	**Edited Universe:**	All activities for respondents with at least one own household child < 13		
	**Valid Entries:**	0<br>1440	Min Value<br>Max Value	
	***Note**	TRTOHH_LN is calculated using TUCC5. It does not include activities with TRTIER2P=0101, 0301, 0302, 0303, or with TRCODEP=180381. It does not include any activity or part of any activity in which no household child was awake (determined by TUCC2 and TUCC4).		
**Name**	**Description**			**File**
TRTOHHCHILD	Total nonwork-related time respondent spent with own household children < 18 (in minutes)			Respondent File
	**Edited Universe:**	All respondents		
	**Valid Entries:**	0<br>1440	Min Value<br>Max Value	
	***Note**	This variable is computed using TUWHO_CODE information; time spent working and all activities for which who information is not collected, such as sleeping, are omitted from the calculation		
**Name**	**Description**			**File**
TRTONHH	Total time spent during diary day providing secondary childcare for own nonhousehold children < 13 (in minutes)			Respondent File
	**Edited Universe:**	All respondents		
	**Valid Entries:**	0<br>1440	Min Value<br>Max Value	
	***Note**	TRTONHH is the sum of all values of TRTONHH_LN for each TUCASEID.		

2003-2023 ATUS Data Dictionary: Public ATUS Interview Data

Name	Description		File
TRTONHH_LN	Total time spent during activity providing secondary childcare for own nonhousehold children < 13 (in minutes)		Activity File
	**Edited Universe:**	All activities for respondents with at least one own nonhousehold child < 13	
	**Valid Entries:**	0<br>1440	Min Value<br>Max Value
	***Note**	TRTONHH_LN is calculated using TUCC7. It does not include activities with TRTIER2P of 0101, 0301, 0302, 0303, 0401, 0402, 0403, and activities with TRCODEP of 180381 or 180481.	
**Name**	**Description**		**File**
TRTONHHCHILD	Total nonwork-related time respondent spent with own nonhousehold children < 18 (in minutes)		Respondent File
	**Edited Universe:**	All respondents	
	**Valid Entries:**	0<br>1440	Min Value<br>Max Value
	***Note**	This variable is computed using TUWHO_CODE information; time spent working and all activities for which who information is not collected, such as sleeping, are omitted from the calculation	
**Name**	**Description**		**File**
TRTSPONLY	Total nonwork-related time respondent spent with spouse only (in minutes)		Respondent File
	**Edited Universe:**	All respondents	
	**Valid Entries:**	0<br>1440	Min Value<br>Max Value
	***Note**	This variable is computed using TUWHO_CODE information; time spent working and all activities for which who information is not collected, such as sleeping, are omitted from the calculation	
**Name**	**Description**		**File**
TRTSPOUSE	Total nonwork-related time respondent spent with spouse (others may be present) (in minutes)		Respondent File
	**Edited Universe:**	All respondents	
	**Valid Entries:**	0<br>1440	Min Value<br>Max Value
	***Note**	This variable is computed using TUWHO_CODE information; time spent working and all activities for which who information is not collected, such as sleeping, are omitted from the calculation	
**Name**	**Description**		**File**
TRTUNMPART	Total nonwork-related time respondent spent with unmarried partner (others may be present) (in minutes)		Respondent File
	**Edited Universe:**	All respondents	
	**Valid Entries:**	0<br>1440	Min Value<br>Max Value
	***Note**	This variable is computed using TUWHO_CODE information; time spent working and all activities for which who information is not collected, such as sleeping, are omitted from the calculation	

2003-2023 ATUS Data Dictionary: Public ATUS Interview Data

Name	Description			File
TRWERNAL	TRERNWA: allocation flag			Respondent File
	**Edited Universe:**	TELFS = 1 or 2 and TEIO1COW = 1 - 5		
	**Valid Entries:**	0	TRERNWA does not contain allocated information	
		1	TRERNWA contains allocated information	
**Name**	**Description**			**File**
TRWHONA	Who information not asked for activity			Who File
	**Edited Universe:**	All activities		
	**Valid Entries:**	0	TUWHO_CODE asked	
		1	TUWHO_CODE not asked	
**Name**	**Description**			**File**
TRYHHCHILD	Age of youngest household child < 18			Respondent File, Activity Summary File
	**Edited Universe:**	TRHHCHILD = 1		
	**Valid Entries:**	0<br>17	Min Value<br>Max Value	
**Name**	**Description**			**File**
TTHR	Hourly pay topcode flag			Respondent File
	**Valid Entries:**	0	Not topcoded	
		1	Topcoded	
	***Note**	Indicates topcoding of hourly pay in earnings variables		
**Name**	**Description**			**File**
TTOT	Overtime amount topcode flag			Respondent File
	**Valid Entries:**	0	Not topcoded	
		1	Topcoded	
	***Note**	Indicates topcoding of overtime pay in earnings variables		
**Name**	**Description**			**File**
TTWK	Weekly earnings topcode flag			Respondent File
	**Valid Entries:**	0	Not topcoded	
		1	Topcoded	
	***Note**	Indicates topcoding of weekly pay in earnings variables		

2003-2023 ATUS Data Dictionary: Public ATUS Interview Data

Name	Description			File
TU20FWGT	ATUS final weight for 2020 data			Respondent File,<br>Activity Summary File
	**Valid Entries:**	1<br>999999999	Min Value<br>Max Value	
	***Note**	When using the 2020 ATUS data, researchers need to use the weighting variable TU20FWGT instead of TUFNWGTP.<br><br>The usual final weight [TUFNWGTP] on the multiyear files will have missing values for cases with [TUYEAR = 2020] on the ATUS Respondent or Activity Summary file.<br><br>Cases with [TUYEAR = 2019 or 2020] will have positive values for diary dates corresponding to January 1 - March 17 or May 10 - December 31.<br><br>For more information about TU20FWGT, see chapter 7 of the ATUS User's Guide.		
**Name**	**Description**			**File**
TUABSOT	In the last seven days, did you have a job either full or part time?			Respondent File
	**Valid Entries:**	1	Yes	
		2	No	
		3	Retired	
		4	Disabled	
		5	Unable to work	
**Name**	**Description**			**File**
TUACTDUR	Duration of activity in minutes (last activity not truncated at 4:00 a.m.)			Activity File
	**Valid Entries:**	1<br>9999	Min Value<br>Max Value	
**Name**	**Description**			**File**
TUACTDUR24	Duration of activity in minutes (last activity truncated at 4:00 a.m.)			Activity File
	**Valid Entries:**	1<br>1440	Min Value<br>Max Value	
**Name**	**Description**			**File**
TUACTIVITY_N	Activity line number			Activity File,<br>Who File,<br>EH Activity File
	**Valid Entries:**	1<br>91	Min Value<br>Max Value	

2003-2023 ATUS Data Dictionary: Public ATUS Interview Data

Name	Description			File
TUBUS	Does anyone in the household own a business or a farm?			Respondent File
	**Valid Entries:**	1	Yes	
		2	No	
**Name**	**Description**			**File**
TUBUS1	In the last seven days, did you do any unpaid work in the family business or farm?			Respondent File
	**Valid Entries:**	1	Yes	
		2	No	
**Name**	**Description**			**File**
TUBUS2OT	Do you receive payments or profits from the business?			Respondent File
	**Valid Entries:**	1	Yes	
		2	No	
**Name**	**Description**			**File**
TUBUSL1	TULINENO of farm or business owner (first owner)			Respondent File
	**Valid Entries:**	0<br>30	Min Value<br>Max Value	
**Name**	**Description**			**File**
TUBUSL2	TULINENO of farm or business owner (second owner)			Respondent File
	**Valid Entries:**	0<br>30	Min Value<br>Max Value	
**Name**	**Description**			**File**
TUBUSL3	TULINENO of farm or business owner (third owner)			Respondent File
	**Valid Entries:**	0<br>30	Min Value<br>Max Value	
**Name**	**Description**			**File**
TUBUSL4	TULINENO of farm or business owner (fourth owner)			Respondent File
	**Valid Entries:**	0<br>30	Min Value<br>Max Value	
**Name**	**Description**			**File**
TUCASEID	ATUS Case ID (14-digit identifier)			All Files
**Name**	**Description**			**File**
TUCC2	Time first household child < 13 woke up			Respondent File
	**Valid Entries:**	00:00:00<br>24:00:00	Min Value<br>Max Value	
**Name**	**Description**			**File**
TUCC4	Time last household child < 13 went to bed			Respondent File
	**Valid Entries:**	00:00:00<br>24:00:00	Min Value<br>Max Value	

2003-2023 ATUS Data Dictionary: Public ATUS Interview Data

Name	Description			File
TUCC5	Was at least one of your own household children < 13 in your care during this activity?			Activity File
	**Valid Entries:**	0	No	
		1	Yes	
		97	No additional activities involved childcare	
**Name**	**Description**			**File**
TUCC5B	Was at least one of your non-own household children < 13 in your care during this activity?			Activity File
	**Valid Entries:**	0	No	
		1	Yes	
		97	No additional activities involved childcare	
**Name**	**Description**			**File**
TUCC7	Was at least one of your own non-household children < 13 in your care during this activity?			Activity File
	**Valid Entries:**	0	No	
		1	Yes	
		97	No additional activities involved childcare	
**Name**	**Description**			**File**
TUCC8	Other than household or own non-household children < 13, was there a child 0-12 in your care during this activity?			Activity File
	**Valid Entries:**	0	No	
		1	Yes	
		97	No additional activities involved childcare	
**Name**	**Description**			**File**
TUCC9	Are the non-own, non-household children you cared for in TUCC8 related to you?			Respondent File
	**Valid Entries:**	1	Yes	
		2	No	
		3	Some are, some are not	
**Name**	**Description**			**File**
TUCUMDUR	Cumulative duration of activity lengths in minutes; last activity not truncated at 4:00am or 1440 minutes (cumulative total of TUACTDUR for each TUCASEID)			Activity File
	**Valid Entries:**	1<br>9999	Min Value<br>Max Value	

2003-2023 ATUS Data Dictionary: Public ATUS Interview Data

Name	Description			File
TUCUMDUR24	Cumulative duration of activity lengths in minutes; last activity truncated at 4:00am or 1440 minutes (cumulative total of TUACTDUR24 for each TUCASEID)			Activity File
	**Valid Entries:**	1<br>1440	Min Value<br>Max Value	
**Name**	**Description**			**File**
TUDIARYDATE	Date of diary day (date about which the respondent was interviewed)			Respondent File
	**Valid Entries:**	20030101<br>20231230	Min Value<br>Max Value	
	***Note**	TUDIARYDATE is in YYYYMMDD format		
**Name**	**Description**			**File**
TUDIARYDAY	Day of the week of diary day (day of the week about which the respondent was interviewed)			Respondent File,<br>Activity Summary File
	**Valid Entries:**	1	Sunday	
		2	Monday	
		3	Tuesday	
		4	Wednesday	
		5	Thursday	
		6	Friday	
		7	Saturday	
**Name**	**Description**			**File**
TUDIS	Last time we spoke to someone in this household you were reported to have a disability. Does your disability prevent you from doing any kind of work for the next six months?			Respondent File
	**Valid Entries:**	1	Yes	
		2	No	
		3	Did not have a disability last time	
**Name**	**Description**			**File**
TUDIS1	Does your disability prevent you from accepting any kind of work during the next six months?			Respondent File
	**Valid Entries:**	1	Yes	
		2	No	
**Name**	**Description**			**File**
TUDIS2	Do you have a disability that prevents you from accepting any kind of work during the next six months?			Respondent File
	**Valid Entries:**	1	Yes	
		2	No	

2003-2023 ATUS Data Dictionary: Public ATUS Interview Data

Name	Description			File
TUDURSTOP	Method for reporting activity duration			Activity File
	**Valid Entries:**	1	Activity duration was entered	
		2	Activity stop time was entered	
	***Note**	Starting in 2012, this variable was added to the public use data files. Cases with [TUYEAR < 2012] will have missing values for TUDURSTOP.<br><br>In 2014, TUDURSTOP was not included on the activity file. Cases with [TUYEAR=2014] will have missing values.		
**Name**	**Description**			**File**
TUEC24	At which times or during which activities did you provide that care or assistance yesterday?			Activity File
	**Valid Entries:**	1	Activity identified as eldercare	
		96	All day	
		97	No more activities	
	***Note**	Questions about eldercare were introduced in January 2011. Therefore, cases with [TUYEAR < 2011] will have missing values for TUEC24		
**Name**	**Description**			**File**
TUECLNO	Line number of eldercare recipient			EC Roster File
	**Valid Entries:**	2<br>35	Min Value<br>Max Value	
	***Note**	If recipient is a household member, TUECLNO = TULINENO; if not a household member, TUECLNO = new line numbers (last tulineno+1)		
**Name**	**Description**			**File**
TUECYTD	Did you provide any eldercare or assistance yesterday?			Respondent File
	**Valid Entries:**	1	Yes	
		2	No	
	***Note**	Questions about eldercare were introduced in January 2011. Therefore, cases with [TUYEAR < 2011] will have missing values for TUECYTD		
**Name**	**Description**			**File**
TUELDER	Not including financial assistance or help you provided as part of your paid job, since the first of [REF_MONTH], have you provided any care of assistance for an adult who needed help because of a condition related to aging?			Respondent File
	**Valid Entries:**	1	Yes	
		2	No	
	***Note**	The reference month is 3 months prior to the interview. For example, if the interview took place on March 15, the reference month would be December.<br><br>Questions about eldercare were introduced in January 2011. Therefore, cases with [TUYEAR < 2011] will have missing values for TUELDER.		

2003-2023 ATUS Data Dictionary: Public ATUS Interview Data

Name	Description		File
TUELFREQ	How often did you provide this care?		Respondent File
	**Valid Entries:**	1	Daily
		2	Several times a week
		3	About once a week
		4	Several times a month
		5	Once a month
		6	One time
		7	Other
	***Note**	Questions about eldercare were introduced in January 2011. Therefore, cases with [TUYEAR < 2011] will have missing values for TUELFREQ	
**Name**	**Description**		**File**
TUELNUM	Since the first of [REF_MONTH], how many people have you provided this care to?		Respondent File
	**Valid Entries:**	0<br>5	Min Value<br>Max Value
	***Note**	The reference month is 3 months prior to the interview. For example, if the interview took place March 15, the reference month is December.<br><br>TUELNUM is topcoded at 5 recipients.<br><br>Questions about eldercare were introduced in January 2011. Therefore, cases with [TUYEAR < 2011] will have missing values for TUELNUM.	
**Name**	**Description**		**File**
TUFNWGTP	ATUS final weight		Respondent File,<br>Activity Summary File
	**Valid Entries:**	0<br>999999999999	Min Value<br>Max Value
	***Note**	This weight uses the 2006 weighting methodology and is comparable for all years. For more information see the ATUS User's Guide.<br><br>Cases with [TUYEAR = 2020] will have missing values for TUFNWGTP. Researchers should use the TU20FWGT weight to construct estimates when using the 2020 data.	
**Name**	**Description**		**File**
TUFWK	In the last seven days did you do any work for pay or profit?		Respondent File
	**Valid Entries:**	1	Yes
		2	No
		3	Retired
		4	Disabled
		5	Unable to work

2003-2023 ATUS Data Dictionary: Public ATUS Interview Data

Name	Description			File
TUIO1MFG	Is this business or organization mainly manufacturing, retail trade, wholesale trade, or something else? (main job)			Respondent File
	**Valid Entries:**	1	Manufacturing	
		2	Retail trade	
		3	Wholesale trade	
		4	Something else	
	***Note**	For TUYEAR < 2005, TUIO1MFG is defined only for respondents whose industry information was updated in the ATUS interview.<br><br>For TUYEAR > = 2005, TUIO1MFG is defined for respondents whose industry information was updated in the ATUS interview; it is populated with CPS values of PUIO1MFG for respondents whose industry information was not updated in the ATUS interview.		
**Name**	**Description**			**File**
TUIODP1	Last time we spoke to someone in this household, you were reported to work for (employer's name). Do you still work for (employer's name)? (main job)			Respondent File
	**Valid Entries:**	1	Yes	
		2	No	
**Name**	**Description**			**File**
TUIODP2	Have the usual activities and duties of your job changed since (month of CPS interview)? (main job)			Respondent File
	**Valid Entries:**	1	Yes	
		2	No	
**Name**	**Description**			**File**
TUIODP3	Last time we spoke to someone in this household, you were reported as (occupation) and your usual duties were (activities). Is this an accurate description of your current job? (main job)			Respondent File
	**Valid Entries:**	1	Yes	
		2	No	
**Name**	**Description**			**File**
TULAY	During the last seven days were you on layoff from your job?			Respondent File
	**Valid Entries:**	1	Yes	
		2	No	
		3	Retired	
		4	Disabled	
		5	Unable to work	

2003-2023 ATUS Data Dictionary: Public ATUS Interview Data

Name	Description			File
TULAY6M	Have you been given any indication that you will be recalled to work within the next 6 months?			Respondent File
	**Valid Entries:**	1	Yes	
		2	No	
**Name**	**Description**			**File**
TULAYAVR	Why could you not have started a job in the last week?			Respondent File
	**Valid Entries:**	1	Own temporary illness	
		2	Going to school	
		3	Other	
**Name**	**Description**			**File**
TULAYDT	Has your employer given you a date to return to work? (to layoff job)			Respondent File
	**Valid Entries:**	1	Yes	
		2	No	
**Name**	**Description**			**File**
TULINENO	ATUS person line number			ATUS-CPS File,<br>Respondent File,<br>Roster File,<br>Who File,<br>EC Roster File
	**Valid Entries:**	1<br>30	Min Value<br>Max Value	
	***Note**	The person selected to be interviewed for ATUS is always TULINENO = 1		
**Name**	**Description**			**File**
TULK	Have you been doing anything to find work during the last four weeks?			Respondent File
	**Valid Entries:**	1	Yes	
		2	No	
		3	Retired	
		4	Disabled	
		5	Unable to work	
**Name**	**Description**			**File**
TULKAVR	Why could you not have started a job last week?			Respondent File
	**Valid Entries:**	1	Waiting for new job to begin	
		2	Own temporary illness	
		3	Going to school	
		4	Other	

2003-2023 ATUS Data Dictionary: Public ATUS Interview Data

Name	Description			File
TULKDK1	You said you have been trying to find work. How did you go about looking? (first method)			Respondent File
	**Valid Entries:**	1	Contacted employer directly/interview	
		2	Contacted public employment agency	
		3	Contacted private employment agency	
		4	Contacted friends or relatives	
		5	Contacted school/university employment center	
		6	Sent out resumes/filled out applications	
		7	Checked union/professional registers	
		8	Placed or answered ads	
		9	Other active	
		10	Looked at ads	
		11	Attended job training programs/courses	
		12	Nothing	
		13	Other passive	
	***Note**	In order to research job search methods, users must combine all fields TELKM1, TULKM2 - TULKM6, TULKDK1 - TULKDK4, and TULKPS1 - TULKPS4		
**Name**	**Description**			**File**
TULKDK2	TULKDK1 text: (second method)			Respondent File
	**Valid Entries:**	1	Contacted employer directly/interview	
		2	Contacted public employment agency	
		3	Contacted private employment agency	
		4	Contacted friends or relatives	
		5	Contacted school/university employment center	
		6	Sent out resumes/filled out applications	
		7	Checked union/professional registers	
		8	Placed or answered ads	
		9	Other active	
		10	Looked at ads	
		11	Attended job training programs/courses	
		13	Other passive	
		97	No additional job search activities	
	***Note**	In order to research job search methods, users must combine all fields TELKM1, TULKM2 - TULKM6, TULKDK1 - TULKDK4, and TULKPS1 - TULKPS4.		
**Name**	**Description**			**File**
TULKDK3	TULKDK1 text: (third method)			Respondent File
	**Valid Entries:**	1<br>97	Min Value<br>Max Value	
	***Note**	See valid values for TULKDK2		

2003-2023 ATUS Data Dictionary: Public ATUS Interview Data

Name	Description		File
TULKDK4	TULKDK1 text: (fourth method)		Respondent File
	**Valid Entries:**	1<br>97	Min Value<br>Max Value
	***Note**	See valid values for TULKDK2	

Name	Description		File
TULKM2	What are all of the things you have done to find work during the last 4 weeks? (second method)		Respondent File
	**Valid Entries:**	1	Contacted employer directly/interview
		2	Contacted public employment agency
		3	Contacted private employment agency
		4	Contacted friends or relatives
		5	Contacted school/university employment center
		6	Sent out resumes/filled out applications
		7	Checked union/professional registers
		8	Placed or answered ads
		9	Other active
		10	Looked at ads
		11	Attended job training programs/courses
		13	Other passive
		97	No additional job search activities
	***Note**	In order to research job search methods, users must combine all fields TELKM1, TULKM2 - TULKM6, TULKDK1 - TULKDK4, and TULKPS1 - TULKPS4.	

Name	Description		File
TULKM3	TULKM2 text: (third method)		Respondent File
	**Valid Entries:**	1<br>97	Min Value<br>Max Value
	***Note**	See valid values for TULKM2	

Name	Description		File
TULKM4	TULKM2 text: (fourth method)		Respondent File
	**Valid Entries:**	1<br>97	Min Value<br>Max Value
	***Note**	See valid values for TULKM2	

Name	Description		File
TULKM5	TULKM2 text: (fifth method)		Respondent File
	**Valid Entries:**	1<br>97	Min Value<br>Max Value
	***Note**	See valid values for TULKM2	

2003-2023 ATUS Data Dictionary: Public ATUS Interview Data

Name	Description			File
TULKM6	TULKM2 text: (sixth method)			Respondent File
	**Valid Entries:**	1<br>97	Min Value<br>Max Value	
	***Note**	See valid values for TULKM2		
**Name**	**Description**			**File**
TULKPS1	Can you tell me more about what you did to search for work? (first method)			Respondent File
	**Valid Entries:**	1	Contacted employer directly/interview	
		2	Contacted public employment agency	
		3	Contacted private employment agency	
		4	Contacted friends or relatives	
		5	Contacted school/university employment center	
		6	Sent out resumes/filled out applications	
		7	Checked union/professional registers	
		8	Placed or answered ads	
		9	Other active	
		10	Looked at ads	
		11	Attended job training programs/courses	
		12	Nothing	
		13	Other passive	
		97	No more job search activities	
	***Note**	In order to research job search methods, users must combine all fields TELKM1, TULKM2 - TULKM6, TULKDK1 - TULKDK4, and TULKPS1 - TULKPS4.		

2003-2023 ATUS Data Dictionary: Public ATUS Interview Data

Name	Description			File
TULKPS2	TULKPS1 text: (second method)			Respondent File
	**Valid Entries:**	1	Contacted employer directly/interview	
		2	Contacted public employment agency	
		3	Contacted private employment agency	
		4	Contacted friends or relatives	
		5	Contacted school/university employment center	
		6	Sent out resumes/filled out applications	
		7	Checked union/professional registers	
		8	Placed or answered ads	
		9	Other active	
		10	Looked at ads	
		11	Attended job training programs/courses	
		13	Other passive	
		97	No additional job search activities	
	***Note**	In order to research job search methods, users must combine all fields TELKM1, TULKM2 - TULKM6, TULKDK1 - TULKDK4, and TULKPS1 - TULKPS4.		
**Name**	**Description**			**File**
TULKPS3	TULKPS1 text: (third method)			Respondent File
	**Valid Entries:**	1<br>97	Min Value<br>Max Value	
	***Note**	See valid values for TULKPS2		
**Name**	**Description**			**File**
TULKPS4	TULKPS1 text: (fourth method)			Respondent File
	**Valid Entries:**	1<br>97	Min Value<br>Max Value	
	***Note**	See valid values for TULKPS2		
**Name**	**Description**			**File**
TUMONTH	Month of diary day (month of day about which ATUS respondent was interviewed)			Respondent File
	**Valid Entries:**	1<br>12	Min Value<br>Max Value	
**Name**	**Description**			**File**
TURETOT	The last time we spoke to someone in this household you were reported to be retired. Are you still retired?			Respondent File
	**Valid Entries:**	1	Yes	
		2	No	
		3	Was not retired last time	

2003-2023 ATUS Data Dictionary: Public ATUS Interview Data

Name	Description			File
TUSPABS	In the last seven days, did your spouse or unmarried partner have a job either full or part time?			Respondent File
	**Valid Entries:**	1	Yes	
		2	No	
		3	Retired	
		4	Disabled	
		5	Unable to work	
**Name**	**Description**			**File**
TUSPUSFT	Does your spouse or unmarried partner usually work 35 hours or more per week?			Respondent File
	**Valid Entries:**	1	Yes	
		2	No	
		3	Hours vary	
		4	No longer has a job	
**Name**	**Description**			**File**
TUSPWK	In the last seven days, did your spouse or unmarried partner do any work for pay or profit?			Respondent File
	**Valid Entries:**	1	Yes	
		2	No	
		3	Retired	
		4	Disabled	
		5	Unable to work	
**Name**	**Description**			**File**
TUSTARTTIM	Activity start time			Activity File
	**Valid Entries:**	00:00:00<br>24:00:00	Min Value<br>Max Value	
**Name**	**Description**			**File**
TUSTOPTIME	Activity stop time			Activity File
	**Valid Entries:**	00:00:00<br>24:00:00	Min Value<br>Max Value	

2003-2023 ATUS Data Dictionary: Public ATUS Interview Data

Name	Description			File
TUWHO_CODE	Who was in the room with you / Who accompanied you?			Who File
	**Valid Entries:**	18	Alone	
		19	Alone	
		20	Spouse	
		21	Unmarried partner	
		22	Own household child	
		23	Grandchild	
		24	Parent	
		25	Brother/sister	
		26	Other related person	
		27	Foster child	
		28	Housemate/roommate	
		29	Roomer/boarder	
		30	Other nonrelative	
		40	Own nonhousehold child < 18	
		51	Parents (not living in household)	
		52	Other nonhousehold family members < 18	
		53	Other nonhousehold family members 18 and older (including parents-in-law)	
		54	Friends	
		55	Co-workers/colleagues/clients	
		56	Neighbors/acquaintances	
		57	Other nonhousehold children < 18	
		58	Other nonhousehold adults 18 and older	
		59	Boss or manager	
		60	People whom I supervise	
		61	Co-workers	
		62	Customers	
	***Note**	Not collected for activities with codes of 0101xx, 0102xx, 0104xx, 500105, or 500106. Also, starting in 2006, not collected for 060101 if respondent is enrolled in high school (TESCHLVL = 1). There is no distinction between 18 and 19. All codes of 40 or greater refer to people living outside of the respondent's household.<br><br>Beginning in January 2010, information was collected for activities with the code 0501xx. For cases with [TUYEAR < 2010] and activity code 0501xx, TUWHO_CODE was not collected.<br><br>Values of 59, 60, 61, and 62 were introduced and the value 55 was dropped in January 2010 ATUS. Therefore, for cases with [TUYEAR < 2010] values 59, 60, 61, and 62 will not appear. For cases with [TUYEAR > 2009], the value 55 will not appear.		

2003-2023 ATUS Data Dictionary: Public ATUS Interview Data

Name	Description			File
TUYEAR	Year of diary day (year of day about which respondent was interviewed)			Respondent File,<br>Activity Summary File
	**Valid Entries:**	2003<br>2023	Min Value<br>Max Value	

APPENDIX A

ATUS industry codes use the U.S. Census Bureau's Industry Classification System, which is based on the North American Industry Classification System. Beginning with the 2020 ATUS, industry data were classified using the **2017 Census Industry Classification System**. This system replaced the 2012 Census Industry Classification System.

The **2012 Census Industry Classification System** was incorporated beginning in the 2014 ATUS; it replaced the 2007 Census Industry Classification System, which was used by ATUS from 2010 until the 2012 classification scheme was implemented.

The **2007 Census Industry Classification System** was incorporated beginning in the 2010 ATUS; it replaced the **2002 Census Industry Classification System**, which was used by ATUS from 2003 until the 2007 classification scheme was implemented.

There were only minor differences between the 2002, 2007, 2012, and 2017 Census Industry Classification Systems. For more information, please see www.bls.gov/cps/cpsoccind.htm.

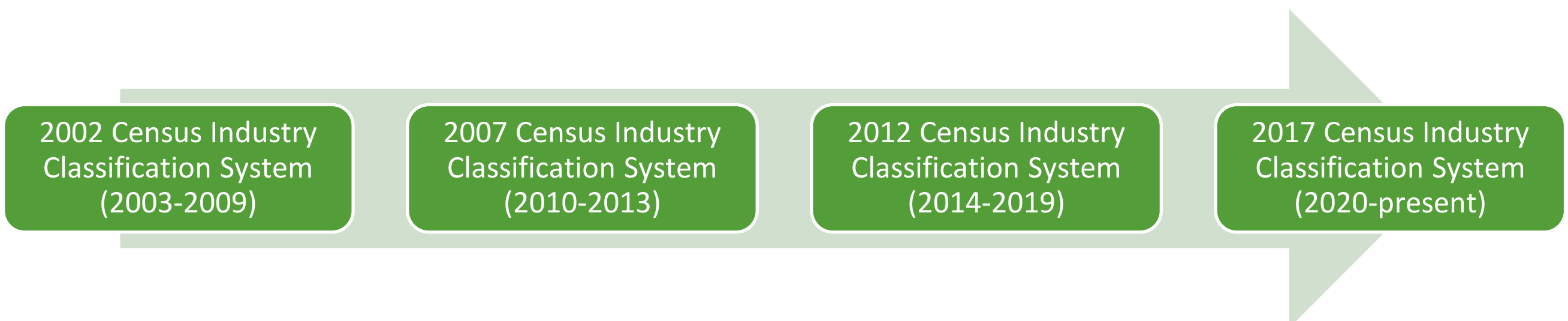

Detailed Industry Code using the 2002 Census Industry Classification system (TRDTIND1)

TRDTIND1	2002 Industry Description	TEIO1ICD
1	Agriculture	0170-0180, 0290
2	Forestry, logging, fishing, hunting, and trapping	0190-0280
3	Mining	0370-0490
4	Construction	770
5	Nonmetallic mineral product manufacturing	2470-2590
6	Primary metals and fabricated metal products	2670-2990
7	Machinery manufacturing	3070-3290
8	Computer and electronic product manufacturing	3360-3390
9	Electrical equipment, appliance manufacturing	3470, 3490
10	Transportation equipment manufacturing	3570-3690
11	Wood product manufacturing	3770-3870
12	Furniture and fixtures manufacturing	3890
13	Miscellaneous and not specified manufacturing	3960-3990
14	Food manufacturing	1070-1290
15	Beverage and tobacco product manufacturing	1370, 1390
16	Textile, apparel, and leather manufacturing	1470-1790
17	Paper manufacturing and printing	1870-1990
18	Petroleum and coal products manufacturing	2070, 2090
19	Chemical manufacturing	2170-2290
20	Plastics and rubber products manufacturing	2370-2390

TRDTIND1	2002 Industry Description	TEIO1ICD
21	Wholesale trade	4070-4590
22	Retail trade	4670-5790
23	Transportation and warehousing	6070-6390
24	Utilities	0570-0690
25	Publishing industries (except internet)	6470-6490
26	Motion picture and sound recording industries	6570, 6590
27	Broadcasting (except internet)	6670
28	Internet publishing and broadcasting	6675
29	Telecommunications	6680, 6690
30	Internet service providers and data processing services	6692, 6695
31	Other information services	6770, 6780
32	Finance	6870-6970
33	Insurance	6990
34	Real estate	7070
35	Rental and leasing services	7080-7190
36	Professional, scientific, and technical services	7270-7490
37	Management of companies and enterprises	7570
38	Administrative and support services	7580-7780
39	Waste management and remediation services	7790
40	Educational services	7860-7890
41	Hospitals	8190
42	Health care services, except hospitals	7970-8180, 8270, 8290
43	Social assistance	8370-8470
44	Arts, entertainment, and recreation	8560-8590
45	Traveler accommodation	8660, 8670
46	Food services and drinking places	8680, 8690
47	Repair and maintenance	8770-8890
48	Personal and laundry services	8970-9090
49	Membership associations and organizations	9160-9190
50	Private households	9290
51	Public administration	9370-9590

Detailed Industry Code using the 2007 Census Industry Classification System (TRDTIND1)

TRDTIND1	2007 Industry Description	TEIO1ICD
1	Agriculture	0170-0180, 0290
2	Forestry, logging, fishing, hunting, and trapping	0190-0280
3	Mining	0370-0490
4	Construction	770
5	Nonmetallic mineral product manufacturing	2470-2590
6	Primary metals and fabricated metal products	2670-2990
7	Machinery manufacturing	3070-3290
8	Computer and electronic product manufacturing	3360-3390
9	Electrical equipment, appliance manufacturing	3470, 3490
10	Transportation equipment manufacturing	3570-3690
11	Wood product manufacturing	3770-3870
12	Furniture and fixtures manufacturing	3890
13	Miscellaneous and not specified manufacturing	3960-3990
14	Food manufacturing	1070-1290
15	Beverage and tobacco product manufacturing	1370, 1390
16	Textile, apparel, and leather manufacturing	1470-1790
17	Paper manufacturing and printing	1870-1990
18	Petroleum and coal products manufacturing	2070, 2090
19	Chemical manufacturing	2170-2290
20	Plastics and rubber products manufacturing	2370-2390
21	Wholesale trade	4070-4590
22	Retail trade	4670-5790
23	Transportation and warehousing	6070-6390
24	Utilities	0570-0690
25	Publishing industries (except internet)	6470-6490
26	Motion picture and sound recording industries	6570, 6590
27	Broadcasting (except internet)	6670
28	Internet publishing and broadcasting	6672
29	Telecommunications	6680, 6690
30	Internet service providers and data processing services	6695
31	Other information services	6770, 6780
32	Finance	6870-6970
33	Insurance	6990
34	Real estate	7070
35	Rental and leasing services	7080-7190
36	Professional, scientific, and technical services	7270-7490
37	Management of companies and enterprises	7570
38	Administrative and support services	7580-7780
39	Waste management and remediation services	7790
40	Educational services	7860-7890
41	Hospitals	8190
42	Health care services, except hospitals	7970-8180, 8270, 8290
43	Social assistance	8370-8470
44	Arts, entertainment, and recreation	8560-8590
45	Traveler accommodation	8660, 8670

TRDTIND1	2007 Industry Description	TEIO1ICD
46	Food services and drinking places	8680, 8690
47	Repair and maintenance	8770-8890
48	Personal and laundry services	8970-9090
49	Membership associations and organizations	9160-9190
50	Private households	9290
51	Public administration	9370-9590

Detailed Industry Code using the 2012 Census Industry Classification System (TRDTIND1)

TRDTIND1	2012 Industry Description	TEIO1ICD
1	Agriculture	0170-0180, 0290
2	Forestry, logging, fishing, hunting, and trapping	0190-0280
3	Mining	0370-0490
4	Construction	770
5	Nonmetallic mineral product manufacturing	2470-2590
6	Primary metals and fabricated metal products	2670-2990
7	Machinery manufacturing	3070-3290
8	Computer and electronic product manufacturing	3365-3390
9	Electrical equipment, appliance manufacturing	3470, 3490
10	Transportation equipment manufacturing	3570-3690
11	Wood products	3770-3875
12	Furniture and fixtures manufacturing	3895
13	Miscellaneous and not specified manufacturing	3960-3990
14	Food manufacturing	1070-1290
15	Beverage and tobacco products	1370, 1390
16	Textile, apparel, and leather manufacturing	1470-1790
17	Paper and printing	1870-1990
18	Petroleum and coal products	2070, 2090
19	Chemical manufacturing	2170-2290
20	Plastics and rubber products	2370-2390
21	Wholesale trade	4070-4590
22	Retail trade	4670-5790
23	Transportation and warehousing	6070-6390
24	Utilities	0570-0690
25	Publishing industries (except internet)	6470-6490
26	Motion picture and sound recording industries	6570, 6590
27	Broadcasting (except internet)	6670
28	Internet publishing and broadcasting	6672
29	Telecommunications	6680, 6690
30	Internet service providers and data processing services	6695
31	Other information services	6770, 6780
32	Finance	6870-6970
33	Insurance	6990
34	Real estate	7070
35	Rental and leasing services	7080-7190
36	Professional and technical services	7270-7490

TRDTIND1	2012 Industry Description	TEIO1ICD
37	Management of companies and enterprises	7570
38	Administrative and support services	7580-7780
39	Waste management and remediation services	7790
40	Educational services	7860-7890
41	Hospitals	8190
42	Health care services, except hospitals	7970-8180, 8270, 8290
43	Social assistance	8370-8470
44	Arts, entertainment, and recreation	8560-8590
45	Accommodation	8660, 8670
46	Food services and drinking places	8680, 8690
47	Repair and maintenance	8770-8890
48	Personal and laundry services	8970-9090
49	Membership associations and organizations	9160-9190
50	Private households	9290
51	Public administration	9370-9590

Detailed Industry Codes using the 2017 Census Industry Classification system (TRDTIND1)

TRDTIND1	2017 Industry Description	TEIO1ICD
1	Agriculture	0170-0180, 0290
2	Forestry, logging, fishing, hunting, and trapping	0190-0280
3	Mining	0370-0490
4	Construction	770
5	Nonmetallic mineral product manufacturing	2470-2590
6	Primary metals and fabricated metal products	2670-2990
7	Machinery manufacturing	3070-3291
8	Computer and electronic product manufacturing	3365-3390
9	Electrical equipment, appliance manufacturing	3470, 3490
10	Transportation equipment manufacturing	3570-3690
11	Wood products	3770-3875
12	Furniture and fixtures manufacturing	3895
13	Miscellaneous and not specified manufacturing	3960-3990
14	Food manufacturing	1070-1290
15	Beverage and tobacco products	1370, 1390
16	Textile, apparel, and leather manufacturing	1470-1790
17	Paper and printing	1870-1990
18	Petroleum and coal products	2070, 2090
19	Chemical manufacturing	2170-2290
20	Plastics and rubber products	2370-2390
21	Wholesale trade	4070-4590
22	Retail trade	4670-5790
23	Transportation and warehousing	6070-6390
24	Utilities	0570-0690
25	Publishing industries (except internet)	6470-6490
26	Motion picture and sound recording industries	6570, 6590
27	Broadcasting (except internet)	6670
28	Internet publishing and broadcasting	6672

TRDTIND1	2017 Industry Description	TEIO1ICD
29	Telecommunications	6680, 6690
30	Internet service providers and data processing services	6695
31	Other information services	6770, 6780
32	Finance	6870-6970
33	Insurance	6991, 6992
34	Real estate	7071, 7072
35	Rental and leasing services	7080-7190
36	Professional and technical services	7270-7490
37	Management of companies and enterprises	7570
38	Administrative and support services	7580-7780
39	Waste management and remediation services	7790
40	Educational services	7860-7890
41	Hospitals	8191, 8192
42	Health care services, except hospitals	7970-8180, 8270, 8290
43	Social assistance	8370-8470
44	Arts, entertainment, and recreation	8560-8590
45	Accommodation	8660, 8670
46	Food services and drinking places	8680, 8690
47	Repair and maintenance	8770-8891
48	Personal and laundry services	8970-9090
49	Membership associations and organizations	9160-9190
50	Private households	9290
51	Public administration	9370-9590

Beginning with the 2020 ATUS, occupation data were classified using the **2018 Census Occupation Classification System**. This system replaced the 2010 Census Occupation Classification System.

The **2010 Census Occupation Classification System** was incorporated beginning in the 2011 ATUS; it replaced the **2002 Census Occupation Classification System**, which was used by ATUS from 2003 until the 2010 classification scheme was implemented.

The ATUS occupation data are not strictly comparable across the years. For more information, please see www.bls.gov/cps/cpsoccind.htm .

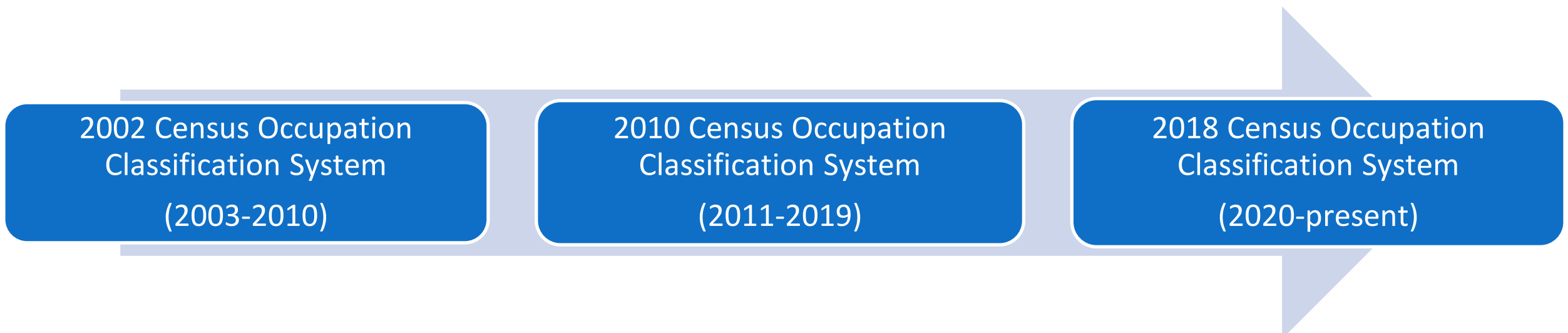

Detailed Occupation Codes using the 2002 Census Occupation Classification system (TRDTOCC1)

Detailed Occupation Code	2002 Occupation Description	Census Occupation Code (TEIO1OCD)
1	Management Occupations	0010–0430
2	Business and financial operations occupations	0500–0950
3	Computer and mathematical science occupations	1000–1240
4	Architecture and engineering occupations	1300–1560
5	Life, Physical, and social science occupations	1600–1960
6	Community and social service occupations	2000–2060
7	Legal occupations	2100–2150
8	Education, training, and library occupations	2200–2550
9	Arts, design, entertainment, sports, and media occupations	2600–2960
10	Healthcare practitioner and technical occupations	3000–3540
11	Healthcare support occupations	3600–3650
12	Protective service occupations	3700–3950
13	Food preparation and serving related occupations	4000–4160
14	Building and grounds cleaning and maintenance occupations	4200–4250
15	Personal care and service occupations	4300–4650
16	Sales and related occupations	4700–4960
17	Office and administrative support occupations	5000–5930
18	Farming, fishing, and forestry occupations	6000–6130
19	Construction and extraction occupations	6200–6940
20	Installation, maintenance, and repair occupations	7000–7620
21	Production occupations	7700–8960
22	Transportation and material moving occupations	9000–9750

Detailed Occupation Codes using the 2010 Census Occupation Classification system (TRDTOCC1)

Detailed Occupation Code	2010 Occupation Description	Census Occupation Code (TEIO1OCD)
1	Management Occupations	0010–0430
2	Business and financial operations occupations	0500–0950
3	Computer and mathematical science occupations	1000–1240
4	Architecture and engineering occupations	1300–1560
5	Life, Physical, and social science occupations	1600–1965
6	Community and social service occupations	2000–2060
7	Legal occupations	2100–2160
8	Education, training, and library occupations	2200–2550
9	Arts, design, entertainment, sports, and media occupations	2600–2960
10	Healthcare practitioner and technical occupations	3000–3540
11	Healthcare support occupations	3600–3655
12	Protective service occupations	3700–3955
13	Food preparation and serving related occupations	4000–4160
14	Building and grounds cleaning and maintenance occupations	4200–4250
15	Personal care and service occupations	4300–4650
16	Sales and related occupations	4700–4965
17	Office and administrative support occupations	5000–5940
18	Farming, fishing, and forestry occupations	6000–6130
19	Construction and extraction occupations	6200–6940
20	Installation, maintenance, and repair occupations	7000–7630
21	Production occupations	7700–8965
22	Transportation and material moving occupations	9000–9750

Detailed Occupation Codes using the 2018 Census Occupation Classification system (PRDTOCC1 and PRDTOCC2)

Detailed Occupation Code	2018 Occupation Description	Census Occupation Code (PEIO1OCD and PEIO2OCD)
1	Management occupations	0010–0440
2	Business and financial operations occupations	0500–0960
3	Computer and mathematical science occupations	1005–1240
4	Architecture and engineering occupations	1305–1560
5	Life, physical, and social science occupations	1600–1980
6	Community and social service occupations	2001–2060
7	Legal occupations	2100–2180
8	Education, training, and library occupations	2205–2555
9	Arts, design, entertainment, sports, and media occupations	2600–2970
10	Healthcare practitioner and technical occupations	3000–3550
11	Healthcare support occupations	3601–3655
12	Protective service occupations	3700–3960
13	Food preparation and serving related occupations	4000–4160
14	Building and grounds cleaning and maintenance occupations	4200–4255
15	Personal care and service occupations	4330–4655
16	Sales and related occupations	4700–4965
17	Office and administrative support occupations	5000–5940
18	Farming, fishing, and forestry occupations	6005–6130
19	Construction and extraction occupations	6200–6950
20	Installation, maintenance, and repair occupations	7000–7640
21	Production occupations	7700–8990
22	Transportation and material moving occupations	9005–9760

Industry Codes (TEIO1ICD)

2002 Census Industry Classification Codes available at https://www.bls.gov/tus/iocodes/census02iocodes.pdf

2007 Census Industry Classification Codes available at https://www.bls.gov/tus/iocodes/census07icodes.pdf

2012 Census Industry Classification Codes available at https://www.bls.gov/tus/iocodes/census12icodes.pdf

2017 Census Industry Classification Codes available at https://www.bls.gov/tus/iocodes/census17icodes.pdf

Occupation Codes (TEIO1OCD)

2002 Census Occupation Classification Codes available at https://www.bls.gov/tus/iocodes/census02iocodes.pdf

2010 Census Occupation Classification Codes available at https://www.bls.gov/tus/iocodes/census10ocodes.pdf

2018 Census Occupation Classification Codes available at https://www.bls.gov/tus/iocodes/census18ocodes.pdf

Appendix B:
Variables on the 2003-23 ATUS Interview files that do ***not*** appear on the single-year ATUS Interview files

The 2003-23 ATUS files include additional variables that are not found on the single-year ATUS files. Appendix B provides a list of variables appearing on the multi-year data files, and *not* on the single-year ATUS files. It also indicates on which multi-year file you can find these variables.

Table B
Variables appearing on the 2003-23 ATUS files, but *not* on single-year ATUS files

Variable name	Description of variable	2003-23 Activity File	2003-23 Activity Summary File	2003-23 Roster File	2003-23 Respondent File	2003-23 Who File
		An "X" indicates the multi-year file on which the variable appears.				
TRCODEP	Multi-year six digit activity code	X				
TRTIER2P	Pooled lexicon tiers 1 and 2: First four digits of multi-year six-digit activity code.	X				
TRTIER1P	Pooled lexicon tier 1: First and second digits of multi-year six-digit activity code.	X				
TUFNWGTP*	ATUS weight corresponding to 2006 weighting method		X		X	

* While TUFNWGTP does appear on the 2003-23 respondent file, this variable does not have values for 2020 cases

Appendix C: Variables on the single-year ATUS Activity files that do ***not*** appear on the 2003-23 ATUS Activity file

The 2003-23 ATUS Activity file includes most, but not all, of the variables available on the single-year ATUS Activity data files. Appendix C provides a list of variables that appear on single-year Activity files, but do *not* appear on the 2003-23 ATUS Activity file. It also indicates where you can find variables not included on the 2003-23 ATUS Activity data file. For example, an "X" in the column titled "2003 ATUS Activity file" indicates that, while the variable does not appear on the 2003-23 ATUS Activity file, it does appear on the 2003 ATUS Activity file. For more information on the variables listed below, please see the single-year data dictionaries, available online at www.bls.gov/tus/dictionaries.htm.

Table C
Variables *not* appearing on the 2003-23 ATUS Activity file, but appearing on single-year ATUS Activity files

Variable name	Description of variable	2003 ATUS Activity file	2004 ATUS Activity file	2005 ATUS Activity file	2006 ATUS Activity file	2007 – 2023 ATUS Activity files
		An "X" indicates the single-year Activity file on which the variable appears.				
TUTIER1CODE	Lexicon Tier 1: 1st and 2nd digits of 6-digit activity code	X	X	X	X	X
TUTIER2CODE	Lexicon Tier 2: 3rd and 4th digits of 6-digit activity code	X	X	X	X	X
TUTIER3CODE	Lexicon Tier 3: 5th and 6th digits of 6-digit activity code	X	X	X	X	X
TXWHERE	TEWHERE: allocation flag	X	X	X	X	X
TRCODE	Six-digit activity code					X
TRTIER2	First and second activity tiers					X

Appendix D:
Variables on the single-year ATUS Respondent files that do *not* appear on the 2003-23 ATUS Respondent File

The 2003-23 ATUS Respondent file includes most, but not all, of the variables available on the single-year ATUS Respondent data files. Appendix D provides a list of variables that appear on the single-year files, but *not* on the 2003-23 ATUS Respondent file. It also indicates where you can find variables not included on the 2003-23 ATUS Respondent data file. For example, an "X" in the column titled "2003 ATUS Respondent file" indicates that, while the variable does not appear on the 2003-23 ATUS Respondent file, it does appear on the 2003 ATUS Respondent file. For more information on the variables listed below, please see the single-year data dictionaries, available online at www.bls.gov/tus/dictionaries.htm.

Table D
Variables *not* appearing on the 2003-23 ATUS Respondent file, but appearing on single-year ATUS Respondent files

Variable name	Description of variable	ATUS 2003 Respondent file	ATUS 2004 Respondent file	ATUS 2005 Respondent file	ATUS 2006 Respondent file	ATUS 2007-2023 Respondent files
		An "X" indicates the single-year Respondent file on which the variable appears				
TU04FWGT	ATUS final weight based on 2004 weighting method	X				
TU06FWGT	ATUS final weight based on 2006 weighting method	X	X	X		
TUBNCHWGT	ATUS benchmark weight	X				
TUCC5B_CK	Reason respondent did not report secondary childcare activities for nonown household children			X	X	X
TUCC5_CK	Reason respondent did not report secondary childcare activities for own household children			X	X	X
TUERN2	Weekly overtime earnings	X	X	X	X	X
TUERNH1C	What is your hourly rate of pay on this job, excluding overtime pay, tips, or commissions?	X	X	X	X	X
TULKDK5	TULKDK1 text: (fifth method)	X	X	X	X	X

Table D
Variables *not* appearing on the 2003-23 ATUS Respondent file, but appearing on single-year ATUS Respondent files, cont

Variable name	Description of variable	ATUS 2003 Respondent file	ATUS 2004 Respondent file	ATUS 2005 Respondent file	ATUS 2006 Respondent file	ATUS 2007-2023 Respondent files
		An “X” indicates the single-year Respondent file on which the variable appears				
TULKDK6	TULKDK1 text: (sixth method)	X	X	X	X	X
TULKPS5	TULKPS1 text: (fifth method)	X	X	X	X	X
TULKPS6	TULKPS1 text: (sixth method)	X	X	X	X	X
TXABSRSN	TEABSRSN: allocation flag	X	X	X	X	X
TXERN	TEERN: allocation flag	X	X	X	X	X
TXERNH1O	TEERNH1O: allocation flag	X	X	X	X	X
TXERNH2	TEERNH2: allocation flag	X	X	X	X	X
TXERNHRO	TEERNHRO: allocation flag	X	X	X	X	X
TXERNHRY	TEERNHRY: allocation flag	X	X	X	X	X
TXERNPER	TEERNPER: allocation flag	X	X	X	X	X
TXERNRT	TEERNRT: allocation flag	X	X	X	X	X
TXERNUOT	TEERNUOT: allocation flag	X	X	X	X	X
TXERNWKP	TEERNWKP: allocation flag	X	X	X	X	X
TXHRFTPT	TEHRFTPT: allocation flag	X	X	X	X	X
TXHRUSL1	TEHRUSL1: allocation flag	X	X	X	X	X
TXHRUSL2	TEHRUSL2: allocation flag	X	X	X	X	X
TXHRUSLT	TEHRUSLT: allocation flag	X	X	X	X	X
TXIO1COW	TEIO1COW: allocation flag	X	X	X	X	X
TXIO1ICD	TEIO1ICD: allocation flag	X	X	X	X	X
TXIO1OCD	TEIO1OCD: allocation flag	X	X	X	X	X
TXLAYAVL	TELAYAVL: allocation flag	X	X	X	X	X
TXLAYLK	TELAYLK: allocation flag	X	X	X	X	X

Table D
Variables *not* appearing on the 2003-23 ATUS Respondent file, but appearing on single-year ATUS Respondent files, cont

Variable name	Description of variable	ATUS 2003 Respondent file	ATUS 2004 Respondent file	ATUS 2005 Respondent file	ATUS 2006 Respondent file	ATUS 2007-2023 Respondent files
		An "X" indicates the single-year Respondent file on which the variable appears.				
TXLFS	TELFS: allocation flag	X	X	X	X	X
TXLKAVL	TELKAVL: allocation flag	X	X	X	X	X
TXLKM1	TELKM1: allocation flag	X	X	X	X	X
TXMJOT	TEMJOT: allocation flag	X	X	X	X	X
TXRET1	TERET1: allocation flag	X	X	X	X	X
TXSCHENR	TESCHENR: allocation flag	X	X	X	X	X
TXSCHFT	TESCHFT: allocation flag	X	X	X	X	X
TXSCHLVL	TESCHLVL: allocation flag	X	X	X	X	X
TXSPEMPNOT	TESPEMPNOT: allocation flag	X	X	X	X	X
TXSPUHRS	TESPUHRS: allocation flag	X	X	X	X	X
TXTCC	TRTCC_LN and TRTCC: allocation flag	X	X	X	X	X
TXTCCTOT	TRTCCTOT_LN and TRTCCTOT: allocation flag		X	X	X	X
TXTCOC	TRTCOC_LN and TRTCOC: allocation flag	X	X	X	X	X
TXTHH	TRTHH_LN and TRTHH: allocation flag		X	X	X	X
TXTNOHH	TRTNOHH_LN and TRTNOHH: allocation flag		X	X	X	X
TXTO	TRTO_LN and TRTO: allocation flag		X	X	X	X
TXTOHH	TRTOHH_LN and TRTOHH: allocation flag		X	X	X	X
TXTONHH	TRTONHH_LN and TRTONHH: allocation flag		X	X	X	X

Appendix E: Variables on the single-year ATUS Roster files that do *not* appear on the 2003-23 ATUS Roster File

The 2003-23 ATUS Roster file includes most, but not all, of the variables available on the single-year ATUS Roster data files. Appendix E provides a list of variables that appear on the single-year ATUS Roster files, but do *not* appear on the 2003-23 ATUS Roster file. It also indicates where you can find variables not included on the 2003-23 ATUS Roster data file. For example, an "X" in the column titled "2003 ATUS Roster file" indicates that, while the variable does *not* appear on the 2003-23 ATUS Roster file, it does appear on the 2003 ATUS Roster file. For more information on the variables listed below, please see the single-year data dictionaries, available online at www.bls.gov/tus/dictionaries.htm.

Table E
Variables *not* appearing on the 2003-23 ATUS Roster file, but appearing on single-year ATUS Roster files

Variable name	Description of variable	2003 ATUS Roster file	2004 ATUS Roster file	2005 ATUS Roster file	2006 ATUS Roster file	2007-2023 ATUS Roster files
		An "X" indicates the single-year Roster file on which the variable appears.				
TURRP	Relationship to ATUS respondent	X	X			
TXAGE	TEAGE: allocation flag	X	X	X	X	X
TXRRP	TERRP: allocation flag	X	X	X	X	X
TXSEX	TESEX: allocation flag	X	X	X	X	X

Appendix F:
Variables on the single-year ATUS Eldercare Roster files that do ***not*** appear on the 2011-23 ATUS Eldercare Roster File

The 2011-23 ATUS Eldercare Roster file includes most, but not all, of the variables available on the single-year ATUS Eldercare Roster data files. Because questions about eldercare were introduced in 2011, there are no single-year Eldercare Roster files from 2003-2010. Appendix F provides a list of variables that appear on the single-year ATUS Eldercare Roster files, but do *not* appear on the 2011-23 ATUS Eldercare Roster file. It also indicates where you can find variables not included on the 2011-23 ATUS Eldercare Roster data file. For example, an "X" in the column titled "2011 ATUS Eldercare Roster file" indicates that, while the variable does *not* appear on the 2011-23 ATUS Eldercare Roster file, it does appear on the 2011 ATUS Eldercare Roster file. For more information on the variables listed below, please see the single-year data dictionaries, available online at www.bls.gov/tus/dictionaries.htm.

Table F:
Variables on the single-year ATUS Eldercare Roster files that do ***not*** appear on the 2011-23 ATUS Eldercare Roster File

Variable name	Description of variable	2011-2023 ATUS Eldercare Roster files
	An "X" indicates the single-year Roster file on which the variable appears.	
TXAGE_EC	TEAGE_EC: allocation flag	X
TXELDUR	TEELDUR: allocation flag	X
TXELWHO	TEELWHO: allocation flag	X
TXELYRS	TEELYRS: allocation flag	X

American Time Use Survey

Activity Lexicon

2003-2023

This document is the activity classification scheme used to code activities collected during the American Time Use Survey into time-use categories.

U.S. Bureau of Labor Statistics

ATUS 2003-2023 Activity coding lexicon				
Major category	First and second-tier categories	6-digit activity code	Activity	Notes
**01 Personal Care Activities**				
	**0101 Sleeping**			
		010101	Sleeping	
		010102	Sleeplessness	
		010199	Sleeping, n.e.c.*	
	**0102 Grooming**			
		010201	Washing, dressing and grooming oneself	
		010299	Grooming, n.e.c.*	
	**0103 Health-related self care**			
		010301	Health-related self care	
		010399	Self care, n.e.c.*	
	**0104 Personal Activities**			
		010401	Personal/Private activities	
		010499	Personal activities, n.e.c.*	
	**0105 Personal Care Emergencies**			
		010501	Personal emergencies	
		010599	Personal care emergencies, n.e.c.*	
	**0199 Personal care, n.e.c.***			
		019999	Personal care, n.e.c.*	
02 Household Activities				
	**0201 Housework**			
		020101	Interior cleaning	
		020102	Laundry	
		020103	Sewing, repairing, & maintaining textiles	
		020104	Storing interior hh items, inc. food	
		020199	Housework, n.e.c.*	
	**0202 Food & Drink Preparation, Presentation, & Clean-up**			
		020201	Food and drink preparation	
		020202	Food presentation	
		020203	Kitchen and food clean-up	
		020299	Food & drink prep, presentation, & clean-up, n.e.c.*	
	**0203 Interior Maintenance, Repair & Decoration**			
		020301	Interior arrangement, decoration, & repairs	
		020302	Building and repairing furniture	
		020303	Heating and cooling	
		020399	Interior maintenance, repair, & decoration, n.e.c.*	
	**0204 Exterior Maintenance, Repair & Decoration**			
		020401	Exterior cleaning	
		020402	Exterior repair, improvements, & decoration	
		020499	Exterior maintenance, repair & decoration, n.e.c.*	
	**0205 Lawn, Garden, and Houseplants**			
		020501	Lawn, garden, and houseplant care	
		020502	Ponds, pools, and hot tubs	
		020599	Lawn and garden, n.e.c.*	
	**0206 Animals and Pets**			
		020681	Care for animals and pets (not veterinary care)	includes 020601 (all years), 020602 (2008 and later years)
		020699	Pet and animal care, n.e.c.*	
	**0207 Vehicles**			
		020701	Vehicle repair and maintenance (by self)	
		020799	Vehicles, n.e.c.*	
	**0208 Appliances, Tools, and Toys**			
		020801	Appliance, tool, and toy set-up, repair, & maintenance (by self)	
		020899	Appliances and tools, n.e.c.*	
	**0209 Household Management**			
		020901	Financial management	
		020902	Household & personal organization and planning	
		020903	HH & personal mail & messages (except e-mail)	
		020904	HH & personal e-mail and messages	
		020905	Home security	
		020999	Household management, n.e.c.*	

ATUS 2003-2023 Activity coding lexicon				
Major category	First and second-tier categories	6-digit activity code	Activity	Notes
	0299 Household activities, n.e.c.*			
		029999	Household activities, n.e.c.*	
03 Caring For & Helping Household (HH) Members				
	0301 Caring For & Helping HH Children			
		030101	Physical care for hh children	
		030102	Reading to/with hh children	
		030103	Playing with hh children, not sports	
		030104	Arts and crafts with hh children	
		030105	Playing sports with hh children	
		030186	Talking with/listening to hh children	includes 030106 (all years), 030107 (2003)
		030108	Organization & planning for hh children	
		030109	Looking after hh children (as a primary activity)	
		030110	Attending hh children's events	
		030111	Waiting for/with hh children	
		030112	Picking up/dropping off hh children	
		030199	Caring for & helping hh children, n.e.c.*	
	0302 Activities Related to HH Children's Education			
		030201	Homework (hh children)	
		030202	Meetings and school conferences (hh children)	
		030203	Home schooling of hh children	
		030204	Waiting associated with hh children's education	
		030299	Activities related to hh child's education, n.e.c.*	
	0303 Activities Related to HH Children's Health			
		030301	Providing medical care to hh children	
		030302	Obtaining medical care for hh children	
		030303	Waiting associated with hh children's health	
		030399	Activities related to hh child's health, n.e.c.*	
	0304 Caring for Household Adults			
		030401	Physical care for hh adults	
		030402	Looking after hh adult (as a primary activity)	
		030403	Providing medical care to hh adult	
		030404	Obtaining medical and care services for hh adult	
		030405	Waiting associated with caring for household adults	
		030499	Caring for household adults, n.e.c.*	
	0305 Helping Household Adults			
		030501	Helping hh adults	
		030502	Organization & planning for hh adults	
		030503	Picking up/dropping off hh adult	
		030504	Waiting associated with helping hh adults	
		030599	Helping household adults, n.e.c.*	
	0399 Caring For & Helping NonHH Members			
		039999	Caring for & helping hh members, n.e.c.*	
04 Caring For & Helping Nonhousehold (NonHH) Members				
	0401 Caring for & Helping Nonhousehold Children			
		040101	Physical care for nonhh children	
		040102	Reading to/with nonhh children	
		040103	Playing with nonhh children, not sports	
		040104	Arts and crafts with nonhh children	
		040105	Playing sports with nonhh children	
		040186	Talking with/listening to nonhh children	Includes 040106 (all years), 040107 (2003)
		040108	Organization & planning for nonhh children	
		040109	Looking after nonhh children (as primary activity)	
		040110	Attending nonhh children's events	
		040111	Waiting for/with nonhh children	
		040112	Dropping off/picking up nonhh children	
		040199	Caring for and helping nonhh children, n.e.c.*	
	0402 Activities Related to Nonhh Children's Education			
		040201	Homework (nonhh children)	
		040202	Meetings and school conferences (nonhh children)	
		040203	Home schooling of nonhh children	
		040204	Waiting associated with nonhh children's education	
		040299	Activities related to nonhh child's educ., n.e.c.*	

ATUS 2003-2023 Activity coding lexicon				
Major category	First and second-tier categories	6-digit activity code	Activity	Notes
	**0403 Activities Related to Nonhh Children's Health**			
		040301	Providing medical care to nonhh children	
		040302	Obtaining medical care for nonhh children	
		040303	Waiting associated with nonhh children's health	
		040399	Activities related to nonhh child's health, n.e.c.*	
	**0404 Caring For Nonhousehold Adults**			
		040401	Physical care for nonhh adults	
		040402	Looking after nonhh adult (as a primary activity)	
		040403	Providing medical care to nonhh adult	
		040404	Obtaining medical and care services for nonhh adult	
		040405	Waiting associated with caring for nonhh adults	
		040499	Caring for nonhh adults, n.e.c.*	
	**0405 Helping Nonhousehold Adults**			
		040501	Housework, cooking, & shopping assistance for nonhh adults	
		040502	House & lawn maintenance & repair assistance for nonhh adults	
		040503	Animal & pet care assistance for nonhh adults	
		040504	Vehicle & appliance maintenance/repair assistance for nonhh adults	
		040505	Financial management assistance for nonhh adults	
		040506	Household management & paperwork assistance for nonhh adults	
		040507	Picking up/dropping off nonhh adult	
		040508	Waiting associated with helping nonhh adults	
		040599	Helping nonhh adults, n.e.c.*	
	**0499 Caring For & Helping Nonhousehold Adults, n.e.c.***			
		049999	Caring for & helping nonhh members, n.e.c.*	
**05 Work & Work-Related Activities**				
	**0501 Working**			
		050101	Work, main job	
		050102	Work, other job(s)	
		050103	Security procedures related to work	
		050189	Working, n.e.c.*	Includes 050104 (2004 and later years); 050199 (all years)
	**0502 Work-Related Activities**			
		050201	Socializing, relaxing, and leisure as part of job	
		050202	Eating and drinking as part of job	
		050203	Sports and exercise as part of job	
		050204	Security procedures as part of job	
		050289	Work-related activities, n.e.c.*	Includes 050205 (2004 and later years); 050299 (all years)
	**0503 Other Income-Generating Activities**			
		050301	Income-generating hobbies, crafts, and food	
		050302	Income-generating performances	
		050303	Income-generating services	
		050304	Income-generating rental property activities	
		050389	Other income-generating activities, n.e.c.*	Includes 050305 (2004 and later years); 050399 (All years)
	**0504 Job Search and Interviewing**			
		050481	Job search activities	Includes 050401 (All years); 050402 (2003-2004)
		050403	Job interviewing	
		050404	Waiting associated with job search or interview	
		050405	Security procedures rel. to job search/interviewing	
		050499	Job search and Interviewing, n.e.c.*	
	**0599 Work and Work-Related Activities, n.e.c.***			
		059999	Work and work-related activities, n.e.c.*	
**06 Education**				
	**0601 Taking Class**			
		060101	Taking class for degree, certification, or licensure	
		060102	Taking class for personal interest	
		060103	Waiting associated with taking classes	
		060104	Security procedures rel. to taking classes	
		060199	Taking class, n.e.c.*	
	**0602 Extracurricular School Activities (Except Sports)**			
		060201	Extracurricular club activities	
		060202	Extracurricular music & performance activities	
		060203	Extracurricular student government activities	

ATUS 2003-2023 Activity coding lexicon				
Major category	First and second-tier categories	6-digit activity code	Activity	Notes
		060289	Education-related extracurricular activities, n.e.c.*	Includes 060204 (2004 and later years); 060299 (all years)
	**0603 Research/Homework**			
		060301	Research/homework for class for degree, certification, or licensure	
		060302	Research/homework for class for pers. interest	
		060303	Waiting associated with research/homework	
		060399	Research/homework n.e.c.*	
	**0604 Registration/Administrative activities**			
		060401	Administrative activities: class for degree, certification, or licensure	
		060402	Administrative activities: class for personal interest	
		060403	Waiting associated w/admin. activities (education)	
		060499	Administrative for education, n.e.c.*	
	**0699 Education, n.e.c.***			
		069999	Education, n.e.c.*	
**07 Consumer Purchases**				
	**0701 Shopping (Store, Telephone, Internet)**			
		070101	Grocery shopping	
		070102	Purchasing gas	
		070103	Purchasing food (not groceries)	
		070104	Shopping, except groceries, food and gas	
		070105	Waiting associated with shopping	
		070199	Shopping, n.e.c.*	
	**0702 Researching Purchases**			
		070201	Comparison shopping	
		070299	Researching purchases, n.e.c.*	
	**0703 Security Procedures Related to Consumer Purchases**			
		070301	Security procedures rel. to consumer purchases	
		070399	Security procedures rel. to consumer purchases, n.e.c.*	
	**0799 Consumer Purchases, n.e.c.***			
		079999	Consumer purchases, n.e.c.*	
**08 Professional & Personal Care Services**				
	**0801 Professional & Personal Care Services**			
		080101	Using paid childcare services	
		080102	Waiting associated w/purchasing childcare svcs	
		080199	Using paid childcare services, n.e.c.*	
	**0802 Financial Services and Banking**			
		080201	Banking	
		080202	Using other financial services	
		080203	Waiting associated w/banking/financial services	
		080299	Using financial services and banking, n.e.c.*	
	**0803 Legal Services**			
		080301	Using legal services	
		080302	Waiting associated with legal services	
		080399	Using legal services, n.e.c.*	
	**0804 Medical and Care Services**			
		080401	Using health and care services outside the home	
		080402	Using in-home health and care services	
		080403	Waiting associated with medical services	
		080499	Using medical services, n.e.c.*	
	**0805 Personal Care Services**			
		080501	Using personal care services	
		080502	Waiting associated w/personal care services	
		080599	Using personal care services, n.e.c.*	
	**0806 Real Estate**			
		080601	Activities rel. to purchasing/selling real estate	
		080602	Waiting associated w/purchasing/selling real estate	
		080699	Using real estate services, n.e.c.*	
	**0807 Veterinary Services (excluding grooming)**			
		080701	Using veterinary services	
		080702	Waiting associated with veterinary services	
		080799	Using veterinary services, n.e.c.*	

ATUS 2003-2023 Activity coding lexicon				
**Major category**	**First and second-tier categories**	**6-digit activity code**	**Activity**	**Notes**
	**0808 Security Procedures Related to Professional/Personal Services**			
		080801	Security procedures rel. to professional/personal svcs.	
		080899	Security procedures rel. to professional/personal svcs n.e.c.*	
	**0899 Professional and Personal Services, n.e.c.***			
		089999	Professional and personal services, n.e.c.*	
**09 Household Services**				
	**0901 Household Services (not done by self)**			
		090101	Using interior cleaning services	
		090102	Using meal preparation services	
		090103	Using clothing repair and cleaning services	
		090104	Waiting associated with using household services	
		090199	Using household services, n.e.c.*	
	**0902 Home Maintenance/Repair/Décor/Construction (not done by self)**			
		090201	Using home maint/repair/décor/construction svcs	
		090202	Waiting associated w/ home main/repair/décor/constr	
		090299	Using home maint/repair/décor/constr services, n.e.c.*	
	**0903 Pet Services (not done by self, not vet)**			
		090301	Using pet services	
		090302	Waiting associated with pet services	
		090399	Using pet services, n.e.c.*	
	**0904 Lawn & Garden Services (not done by self)**			
		090401	Using lawn and garden services	
		090402	Waiting associated with using lawn & garden services	
		090499	Using lawn and garden services, n.e.c.*	
	**0905 Vehicle Maintenance & Repair Services (not done by self)**			
		090501	Using vehicle maintenance or repair services	
		090502	Waiting associated with vehicle main. or repair svcs	
		090599	Using vehicle maint. & repair svcs, n.e.c.*	
	**0999 Household Services, n.e.c.***			
		099999	Using household services, n.e.c.*	
**10 Government Services & Civic Obligations**				
	**1001 Using Government Services**			
		100101	Using police and fire services	
		100102	Using social services	
		100103	Obtaining licenses & paying fines, fees, taxes	
		100199	Using government services, n.e.c.*	
	**1002 Civic Obligations & Participation**			
		100201	Civic obligations & participation	
		100299	Civic obligations & participation, n.e.c.*	
	**1003 Waiting Associated w/ Government Services or Civic Obligations**			
		100381	Waiting associated with using government services	Includes 100301 (2003-2007), 100302 (2003-2007), 100304 (2008 and later years).
		100383	Waiting associated w/civic obligations & participation	Includes 100303 (2003-2007), 100305 (2008 and later years)
		100399	Waiting assoc. w/govt svcs or civic obligations, n.e.c.*	
	**1004 Security Procedures Related to Government Services or Civic Obligations**			
		100401	Security procedures rel. to govt svcs/civic obligations	
		100499	Security procedures rel. to govt svcs/civic obligations, n.e.c.*	
	**1099 Government services, n.e.c.***			
		109999	Government services, n.e.c.*	
**11 Eating and Drinking**				
	**1101 Eating and Drinking**			
		110101	Eating and drinking	
		110199	Eating and drinking, n.e.c.*	
	**1102 Waiting associated with Eating & Drinking**			
		110281	Waiting associated w/eating & drinking	Includes 110201 (All years); 110202, 110203 (2003)
		110289	Waiting associated with eating & drinking, n.e.c.*	Includes 110204 (2003); 110299 (All years)
	**1199 Eating and Drinking, n.e.c.***			
		119999	Eating and drinking, n.e.c.*	
**12 Socializing, Relaxing, and Leisure**				
	**1201 Socializing and Communicating**			
		120101	Socializing and communicating with others	

Major category	First and second-tier categories	6-digit activity code	Activity	Notes
		120199	Socializing and communicating, n.e.c.*	
	**1202 Attending or Hosting Social Events**			
		120201	Attending or hosting parties/receptions/ceremonies	
		120202	Attending meetings for personal interest (not volunteering)	
		120299	Attending/hosting social events, n.e.c.*	
	**1203 Relaxing and Leisure**			
		120301	Relaxing, thinking	
		120302	Tobacco and drug use	
		120303	Television and movies (not religious)	
		120304	Television (religious)	
		120305	Listening to the radio	
		120306	Listening to/playing music (not radio)	
		120307	Playing games	
		120308	Computer use for leisure (exc. Games)	
		120309	Arts and crafts as a hobby	
		120310	Collecting as a hobby	
		120311	Hobbies, except arts & crafts and collecting	
		120312	Reading for personal interest	
		120313	Writing for personal interest	
		120399	Relaxing and leisure, n.e.c.*	
	**1204 Arts and Entertainment (other than sports)**			
		120401	Attending performing arts	
		120402	Attending museums	
		120403	Attending movies/film	
		120404	Attending gambling establishments	
		120405	Security procedures rel. to arts & entertainment	
		120499	Arts and entertainment, n.e.c.*	
	**1205 Waiting associated with Socializing, Relaxing, and Leisure**			
		120501	Waiting assoc. w/socializing & communicating	
		120502	Waiting assoc. w/attending/hosting social events	
		120503	Waiting associated with relaxing/leisure	
		120504	Waiting associated with arts & entertainment	
		120599	Waiting associated with socializing, n.e.c.*	
	**1299 Socializing, Relaxing, and Leisure, n.e.c.***			
		129999	Socializing, relaxing, and leisure, n.e.c.*	
**13 Sports, Exercise, & Recreation**				
	**1301 Participating in Sports, Exercise, and Recreation**			
		130101	Doing aerobics	
		130102	Playing baseball	
		130103	Playing basketball	
		130104	Biking	
		130105	Playing billiards	
		130106	Boating	
		130107	Bowling	
		130108	Climbing, spelunking, caving	
		130109	Dancing	
		130110	Participating in equestrian sports	
		130111	Fencing	
		130112	Fishing	
		130113	Playing football	
		130114	Golfing	
		130115	Doing gymnastics	
		130116	Hiking	
		130117	Playing hockey	
		130118	Hunting	
		130119	Participating in martial arts	
		130120	Playing racquet sports	
		130121	Participating in rodeo competitions	
		130122	Rollerblading	
		130123	Playing rugby	
		130124	Running	
		130125	Skiing, ice skating, snowboarding	

ATUS 2003-2023 Activity coding lexicon				
Major category	First and second-tier categories	6-digit activity code	Activity	Notes
		130126	Playing soccer	
		130127	Softball	
		130128	Using cardiovascular equipment	
		130129	Vehicle touring/racing	
		130130	Playing volleyball	
		130131	Walking	
		130132	Participating in water sports	
		130133	Weightlifting/strength training	
		130134	Working out, unspecified	
		130135	Wrestling	
		130136	Doing yoga	
		130199	Playing sports n.e.c.*	
	**1302 Attending Sports/Recreational Events**			
		130201	Watching aerobics	
		130202	Watching baseball	
		130203	Watching basketball	
		130204	Watching biking	
		130205	Watching billiards	
		130206	Watching boating	
		130207	Watching bowling	
		130208	Watching climbing, spelunking, caving	
		130209	Watching dancing	
		130210	Watching equestrian sports	
		130211	Watching fencing	
		130212	Watching fishing	
		130213	Watching football	
		130214	Watching golfing	
		130215	Watching gymnastics	
		130216	Watching hockey	
		130217	Watching martial arts	
		130218	Watching racquet sports	
		130219	Watching rodeo competitions	
		130220	Watching rollerblading	
		130221	Watching rugby	
		130222	Watching running	
		130223	Watching skiing, ice skating, snowboarding	
		130224	Watching soccer	
		130225	Watching softball	
		130226	Watching vehicle touring/racing	
		130227	Watching volleyball	
		130228	Watching walking	
		130229	Watching water sports	
		130230	Watching weightlifting/strength training	
		130231	Watching people working out, unspecified	
		130232	Watching wrestling	
		130299	Attending sporting events, n.e.c.*	
	**1303 Waiting Associated with Sports, Exercise, & Recreation**			
		130301	Waiting related to playing sports or exercising	
		130302	Waiting related to attending sporting events	
		130399	Waiting associated with sports, exercise, & recreation, n.e.c.*	
	**1304 Security Procedures Related to Sports, Exercise, & Recreation**			
		130401	Security related to playing sports or exercising	
		130402	Security related to attending sporting events	
		130499	Security related to sports, exercise, & recreation, n.e.c.*	
	**1399 Sports, Exercise, and Recreation, n.e.c.***			
		139999	Sports, exercise, & recreation, n.e.c.*	
**14 Religious and Spiritual Activities**				
	**1401 Religious/Spiritual Practices**			
		140101	Attending religious services	
		140102	Participation in religious practices	
		140103	Waiting associated w/religious & spiritual activities	
		140104	Security procedures rel. to religious & spiritual activities	

Major category	First and second-tier categories	6-digit activity code	Activity	Notes
		140105	Religious education activities	The activity category "Religious education activities" (140105) was added to the lexicon in 2007. Examples previously classified as "Taking class for personal interest" (060102) were moved to 140105. This re-classification had a small impact on the ATUS estimates for these categories.
	**1499 Religious and Spiritual Practices, n.e.c.***			
		149999	Religious and spiritual activities, n.e.c.*	
**15 Volunteer Activities**				
	**1501 Administrative & Support Activities**			
		150101	Computer use	
		150102	Organizing and preparing	
		150103	Reading	
		150104	Telephone calls (except hotline counseling)	
		150105	Writing	
		150106	Fundraising	
		150199	Administrative & support activities, n.e.c.*	
	**1502 Social Service & Care Activities (Except Medical)**			
		150201	Food preparation, presentation, clean-up	
		150202	Collecting & delivering clothing & other goods	
		150203	Providing care	
		150204	Teaching, leading, counseling, mentoring	
		150299	Social service & care activities, n.e.c.*	
	**1503 Indoor & Outdoor Maintenance, Building, & Clean-up Activities**			
		150301	Building houses, wildlife sites, & other structures	
		150302	Indoor & outdoor maintenance, repair, & clean-up	
		150399	Indoor & outdoor maintenance, building & clean-up activities, n.e.c.*	
	**1504 Participating in Performance & Cultural Activities**			
		150401	Performing	
		150402	Serving at volunteer events & cultural activities	
		150499	Participating in performance & cultural activities, n.e.c.*	
	**1505 Attending Meetings, Conferences, & Training**			
		150501	Attending meetings, conferences, & training	
		150599	Attending meetings, conferences, & training, n.e.c.*	
	**1506 Public Health & Safety Activities**			
		150601	Public health activities	
		150602	Public safety activities	
		150699	Public health & safety activities, n.e.c.*	
	**1599 Volunteer Activities, n.e.c.***			Includes Waiting Associated with Volunteer Activities;<br>Security Procedures Associated with Volunteer Activities;<br>Volunteer activities, n.e.c.*
		159989	Volunteer activities, n.e.c.*	Includes 150701, 150799 (2004 and later years);<br>150801, 150899 (2007 and later years); 159999 (All years)
**16 Telephone Calls**				
	**1601 Telephone Calls (to or from)**			
		160101	Telephone calls to/from family members	
		160102	Telephone calls to/from friends, neighbors, or acquaintances	
		160103	Telephone calls to/from education services providers	
		160104	Telephone calls to/from salespeople	
		160105	Telephone calls to/from professional or personal care svcs providers	
		160106	Telephone calls to/from household services providers	
		160107	Telephone calls to/from paid child or adult care providers	
		160108	Telephone calls to/from government officials	
	**1699 Telephone Calls, n.e.c.***			Includes Waiting Associated with telephone calls;<br>Telephone calls (to or from), n.e.c.*;<br>Telephone calls, n.e.c.*
		169989	Telephone calls, n.e.c.*	Includes 160199, 160201, 160299 (2004 and later years); 169999 (All years)
**18 Traveling**				
	**1801 Travel Related to Personal Care**			
		180101	Travel related to personal care	includes 170101 (2003-2004), 180101 (2005 and later years)
		180199	Travel related to personal care, n.e.c.*	includes 170199 (2003-2004), 180199 (2005 and later years)
	**1802 Travel Related to Household Activities**			

ATUS 2003-2023 Activity coding lexicon				
Major category	First and second-tier categories	6-digit activity code	Activity	Notes
		180280	Travel related to household activities	Includes<br>170201, 170202, 170299 (2003-2004);<br>170203, 170204, 170205, 170206, 170207, 170208, 170209 (2004);<br>180201, 180202, 180203, 180204, 180205, 180206, 180207, 180208, 180209, 180299 (2005 and later years)
	**1803 Travel Related to Caring For & Helping Household Members**			
		180381	Travel related to caring for and helping hh children	Includes 170301 (2003, 2004); 180301, 180302, 180303 (2005 and later years)
		180382	Travel related to caring for and helping hh adults	Includes 170302 (2003, 2004); 180304, 180305 (2005 and later years)
		180399	Travel rel. to caring for & helping hh members, n.e.c.*	Includes 170399 (2003-2004); 180399 (2005 and later years)
	**1804 Travel Related to Caring For & Helping Nonhousehold Members**			
		180481	Travel related to caring for and helping nonhh children	Includes 170401 (2003-2004); 180401, 180402, 180403 (2005 and later years)
		180482	Travel related to caring for and helping nonhh adults	Includes 170402 (2003-2004); 180404, 180405 (2005 and later years)
		180499	Travel rel. to caring for & helping nonhh members, n.e.c.*	includes 170499 (2003-2004); 180499 (2005 and later years)
	**1805 Travel Related to Work**			
		180501	Travel related to working	includes 170501 (2003-2004); 180501 (2005 and later years)
		180502	Travel related to work-related activities	includes 170502 (2003-2004); 180502 (2005 and later years)
		180589	Travel related to work, n.e.c.*	Includes 170503, 170504 (2004); 170599 (2003-2004);<br>180503, 180504, 180599 (2005 and later years)
	**1806 Travel Related to Education**			
		180601	Travel related to taking class	includes 170601 (2003-2004); 180601 (2005 and later years)
		180682	Travel related to education (except taking class)	Includes 170602 (2003, 2004); 180602, 180603, 180604 (2005 and later years)
		180699	Travel related to education, n.e.c.*	includes 170699 (2003-2004); 180699 (2005 and later years)
	**1807 Travel Related to Consumer Purchases**			
		180701	Travel related to grocery shopping	includes 170701 (2003-2004); 180701 (2005 and later years)
		180782	Travel related to shopping (except grocery shopping)	Includes 170702, 170799 (2003-2004); 170703 (2004);<br>180702, 180703, 180704, 180799 (2005 and later years)
	**1808 Travel Related to Using Professional and Personal Care Services**			
		180801	Travel related to using childcare services	includes 170801 (2003-2004); 180801 (2005 and later years)
		180802	Travel related to using financial services and banking	includes 170802 (2003-2004); 180802 (2005 and later years)
		180803	Travel related to using legal services	includes 170803 (2003-2004); 180803 (2005 and later years)
		180804	Travel related to using medical services	includes 170804 (2003-2004); 180804 (2005 and later years)
		180805	Travel related to using personal care services	includes 170805 (2003-2004); 180805 (2005 and later years)
		180806	Travel related to using real estate services	includes 170806 (2003-2004); 180806 (2005 and later years)
		180807	Travel related to using veterinary services	includes 170807 (2003-2004); 180807 (2005 and later years)
		180899	Travel rel. to using prof. & personal care services, n.e.c.*	includes 170899 (2003-2004); 180899 (2005 and later years)
	**1809 Travel Related to Using Household Services**			
		180901	Travel related to using household services	includes 170901 (2003-2004); 180901 (2005 and later years)
		180902	Travel related to using home main./repair/décor./construction svcs	includes 170902 (2003-2004); 180902 (2005 and later years)
		180903	Travel related to using pet services (not vet)	includes 170903 (2003-2004); 180903 (2005 and later years)
		180904	Travel related to using lawn and garden services	includes 170904 (2003-2004); 180904 (2005 and later years)
		180905	Travel related to using vehicle maintenance & repair services	includes 170905 (2003-2004); 180905 (2005 and later years)
		180999	Travel related to using household services, n.e.c.*	includes 170999 (2003-2004); 180999 (2005 and later years)
	**1810 Travel Related to Using Government Services & Civic Obligations**			
		181081	Travel related to using government services	includes 171001, 171002, 171003 (2003-2004); 181001 (2005 and later years)
		181002	Travel related to civic obligations & participation	includes 171004 (2003-2004); 181002 (2005 and later years)
		181099	Travel rel. to govt svcs & civic obligations, n.e.c.*	includes 171099 (2003-2004); 181099 (2005 and later years)
	**1811 Travel Related to Eating and Drinking**			
		181101	Travel related to eating and drinking	includes 171101 (2003-2004); 181101 (2005 and later years)
		181199	Travel related to eating and drinking, n.e.c.*	includes 171199 (2003-2004); 181199 (2005 and later years)
	**1812 Travel Related to Socializing, Relaxing, and Leisure**			
		181201	Travel related to socializing and communicating	includes 171201 (2003-2004); 181201 (2005 and later years)
		181202	Travel related to attending or hosting social events	includes 171202 (2003-2004); 181202 (2005 and later years)
		181283	Travel related to relaxing and leisure	Includes 171203 (2003-2004); 181203, 181205 (2005 and later years)
		181204	Travel related to arts and entertainment	includes 171204 (2003-2004); 181204 (2005 and later years)
		181299	Travel rel. to socializing, relaxing, & leisure, n.e.c.*	includes 171299 (2003-2004); 181299 (2005 and later years)
	**1813 Travel Related to Sports, Exercise, and Recreation**			
		181301	Travel related to participating in sports/exercise/recreation	includes 171301 (2003-2004); 181301 (2005 and later years)
		181302	Travel related to attending sporting/recreational events	includes 171302 (2003-2004); 181302 (2005 and later years)
		181399	Travel related to sports, exercise, & recreation, n.e.c.*	includes 171399 (2003-2004); 181399 (2005 and later years)
	**1814 Travel Related to Religious/Spiritual Activities**			
		181401	Travel related to religious/spiritual practices	includes 171401 (2003-2004); 181401 (2005 and later years)
		181499	Travel rel. to religious/spiritual activities, n.e.c.*	includes 171499 (2003-2004); 181499 (2005 and later years)

ATUS 2003-2023 Activity coding lexicon				
Major category	First and second-tier categories	6-digit activity code	Activity	Notes
	**1815 Travel Related to Volunteer Activities**			
		181501	Travel related to volunteering	includes 171501 (2003-2004); 181501 (2005 and later years)
		181599	Travel related to volunteer activities, n.e.c.*	includes 171599 (2003-2004); 181599 (2005 and later years)
	**1816 Travel Related to Telephone Calls**			
		181601	Travel related to phone calls	includes 171601 (2003-2004); 181601 (2005 and later years)
		181699	Travel rel. to phone calls, n.e.c.*	includes 171699 (2003-2004); 181699 (2005 and later years)
	**1818 Security Procedures Related to Traveling**			
		181801	Security procedures related to traveling	includes 171701 (2003-2004); 181801 (2005 and later years)
		181899	Security procedures related to traveling, n.e.c.*	includes 171799 (2003-2004); 181899 (2005 and later years)
	**1899 Traveling, n.e.c.***			
		189999	Traveling, n.e.c.*	includes 179999 (2003-2004); 189999 (2005 and later years)
**50 Data Codes**				
	**5001 Unable to Code**			
		500101	Insufficient detail in verbatim	
		500103	Missing travel or destination	
		500104	Recorded simultaneous activities incorrectly	code was used in 2003-2012 only
		500105	Respondent refused to provide information/"none of your business"	
		500106	Gap/can't remember	
		500107	Unable to code activity at 1st tier	
	**5099 Data codes, n.e.c.***			
		509989	Data codes, n.e.c.*	includes 500102 (2003); 509999 (All years)
